# Design, synthesis, and bioactivity of ferulic acid derivatives containing an *β*-amino alcohol

**DOI:** 10.1186/s13065-022-00828-8

**Published:** 2022-05-17

**Authors:** Ali Dai, Yuanqin Huang, Lijiao Yu, Zhiguo Zheng, Jian Wu

**Affiliations:** State Key Laboratory Breeding Base of Green Pesticide and Agricultural Bioengineering, Key Laboratory of Green Pesticide and Agricultural Bioengineering, Ministry of Education, Guizhou University, Huaxi District, Guiyang, 550025 China

**Keywords:** Ferulic acid derivatives, *β*-amino alcohol, Synthesized, Antiviral activity, Antibacterial activity

## Abstract

**Background:**

Plant diseases caused by viruses and bacteria cause huge economic losses due to the lack of effective control agents. New potential pesticides can be discovered through biomimetic synthesis and structural modification of natural products. A series of ferulic acid derivatives containing an *β*-amino alcohol were designed and synthesized, and their biological activities were evaluated.

**Result:**

Bioassays results showed that the EC_50_ values of compound **D24** against *Xanthomonas oryzae* pv. *oryzae* (*Xoo*) was 14.5 μg/mL, which was better than that of bismerthiazol (BT, EC_50_ = 16.2 μg/mL) and thiodiazole copper (TC, EC_50_ = 44.5 μg/mL). The in vivo curative and protective activities of compound **D24** against *Xoo* were 50.5% and 50.1%, respectively. The inactivation activities of compounds **D2, D3** and **D4** against tobacco mosaic virus (TMV) at 500 μg/mL were 89.1, 93.7 and 89.5%, respectively, superior to ningnanmycin (93.2%) and ribavirin (73.5%). In particular, the EC_50_ value of compound **D3** was 38.1 μg/mL, and its molecular docking results showed that compound **D3** had a strong affinity for TMV-CP with a binding energy of − 7.54 kcal/mol, which was superior to that of ningnanmycin (− 6.88 kcal /mol).

**Conclusions:**

The preliminary mechanism research results indicated that compound **D3** may disrupt the three-dimensional structure of the TMV coat protein, making TMV particles unable to self-assemble, which may provide potential lead compounds for the discovery of novel plant antiviral agents.

**Graphical Abstract:**

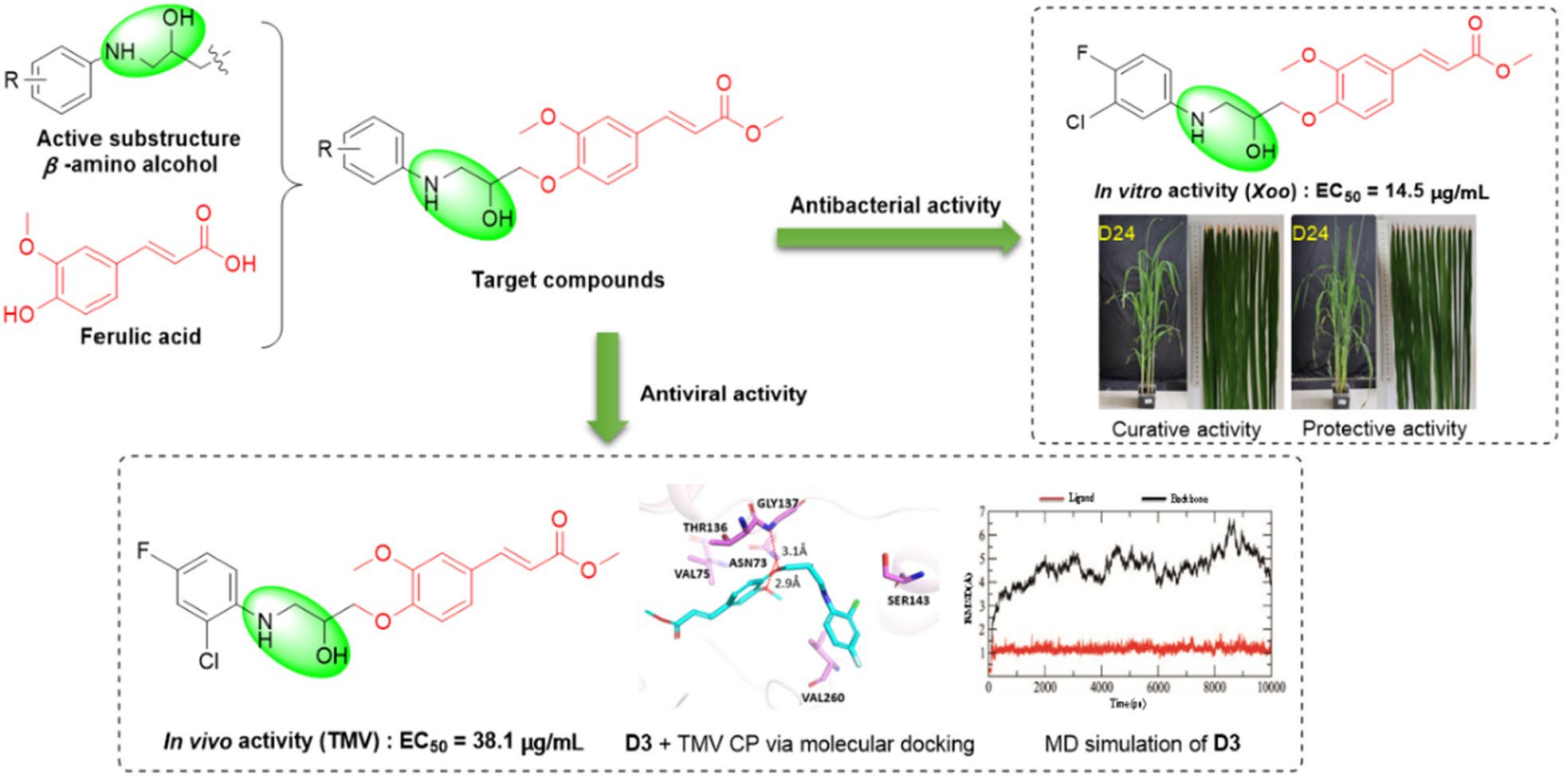

**Supplementary Information:**

The online version contains supplementary material available at 10.1186/s13065-022-00828-8.

## Introduction

Plant pathogens, pests and various abiotic stresses cause serious losses to agricultural production, and are significant problems in achieving agricultural sustainability [1]. So far, more than 1000 plant viruses have been reported [2]. Plant viruses cause huge economic losses to agriculture all over the world every year, amounting to a loss of about $60 billion (USD) in global annual crop yield [2, 3]. Cucumber mosaic virus (CMV) and tobacco mosaic virus (TMV) are the most common plant viruses. TMV infects crops easily and can overwinter on a variety of plants, and few antiviral drugs can effectively control TMV infection [3, 4]. Plant pathogenic bacteria include *Xanthomonas oryzae* pv. *oryzae (Xoo)*, which causes rice bacterial blight with crop yield loss of up to 50% [5, 6], and *Xanthomonas axonopodis* pv*.* *citri (Xac)*, which causes citrus canker [7]. Extant pesticides for these diseases are ineffective, so new pesticides need to be discovered.

**Fig. 1:**
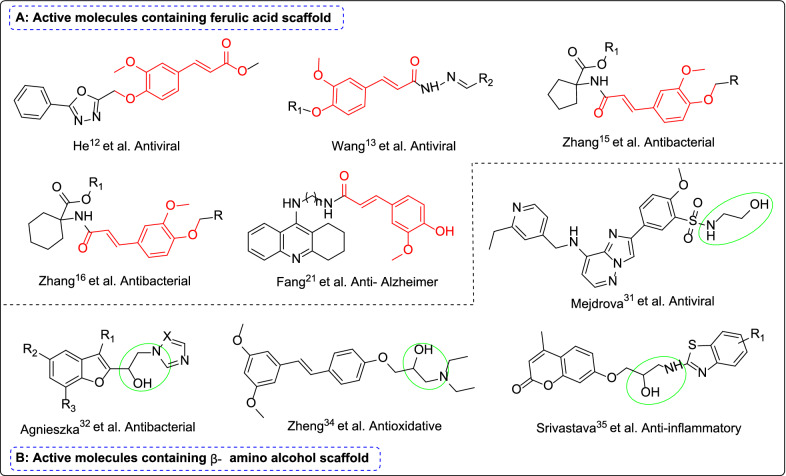
Active molecules containing ferulic acid and *β*-amino alcohol scaffolds

Natural products are a potential alternative for developing new pesticides thanks to their low toxicity to mammals, easy decomposition, environmental friendliness, and unique mode of action [8, 9]. One such product is ferulic acid (FA), the most abundant phenolic acid in the plant. It is cross-linked with polysaccharides and lignin in the structure of the cell wall. It is abundant in *Angelica sinensis, Cimicifuga *spp. and *Ligusticum chuanxiong*, and can be isolated from fruits, vegetables, grains, and coffee beans [2, 4, 10]. Ferulic acid, an *α, β*-unsaturated carboxylic acid structure, has antiviral properties [11–14]. Some phenolic plant extracts containing ferulic acid can inhibit pathogenic bacteria like *Shigella sonnei, Bacillus pneumoniae, Escherichia coli, Citrobacter*, and *Pseudomonas aeruginosa* [15–19]. In addition, the antioxidant effect of ferulic acid has been verified in several acute and chronic pathologies, such as intestinal ischemia, cardiovascular disease, skin disease, and diabetes [20, 21]. Ferulic acid also has anti-inflammatory [22], anti-cancer activity [23, 24], and is a free radical scavenger and an inhibitor or depolymerizer of amyloid structure [20]. Some of the active compounds containing ferulic acid scaffold are shown in Fig. 1A.

The *β*-amino alcohol fragment is a common substructure used as a chiral ligand or an auxiliary in asymmetric synthesis, and plays an important role in pharmaceutical chemistry, medicine and organic synthesis [25–28]. A large part of the literature on asymmetric amino hydroxylation focuses on its application in the synthesis of bioactive compounds, many *β*-amino alcohol derivatives have widely been concerned for their good biological activity [29, 30]. Including antiviral [31], antibacterial [32, 33], antioxidative [34], anti-inflammatory [35], anti-proliferative [36], and anti-cancer [37] properties. Some of the active compounds containing *β*-amino alcohol scaffold are exemplified in Fig. 1B.

**Fig. 2:**
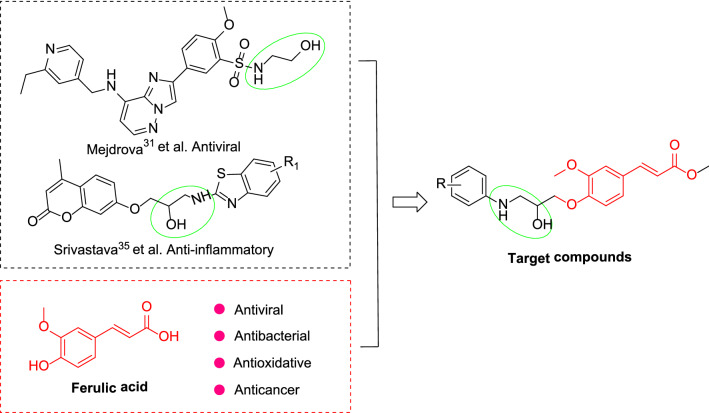
Design strategy of the target compounds

In conclusion, biomimetic synthesis and structural modification of lead compounds of natural products are used to find new pesticides with strong biological activity. In the work described in this paper, ferulic acid derivatives were designed and synthesized in search of highly active compounds, providing potential lead compounds for the discovery of novel plant bactericides and antivirals. Ferulic acid was used as the lead compound, and an *β*-amino alcohol structure was created by etherifying the phenolic hydroxyl site with an appropriate pesticide molecule. This process synthesized a series of ferulic acid derivatives containing an *β*-amino alcohol (Fig. 2), and evaluated the antibacterial and antiviral activities of the target compounds.

## Experimental

### Chemistry

**Scheme 1:**
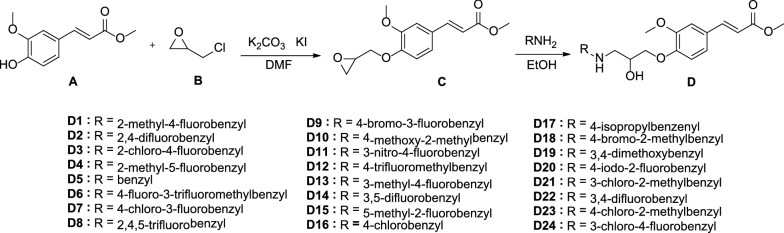
The synthetic route of the target compounds **D1–D24**

Compounds **D1–D24** can be easily obtained by reported methods [34, 38]. The synthetic route for preparation of the target compounds is depicted in Scheme 1. Under alkaline conditions, methyl ferulate **A** is substituted with epichlorohydrin **B** to obtain intermediate **C**. Then, intermediate **C** undergoes a ring-opening reaction with different substituted amines to obtain target compounds **D1–D24**.

### Materials and methods

All reagents and solvents were purchased from commercial companies without further purification and drying. Melting points of synthetic compounds were determined using an XT-4 micro melting point instrument (Beijing Tech Instrument Co., China). All reactions were monitored by thin-layer chromatography (TLC) and identified by UV. The data for ^1^H, ^13^C and ^19^F NMR of title compounds were obtained with AVANCE III HD 400 MHz (Bruker Corporation, Switzerland) or JEOL-ECX 500 MHz (Japan Electronics Corporation), and used TMS as an internal standard at room temperature. High-resolution mass spectrometer (HR-MS) data was conducted using an Orbitrap LC–MS instrument (Q-Exative, Thermo Scientific™, USA).

### General procedure for the preparation of intermediate C

Methyl (*E*)-3-(4-hydroxy-3-methoxyphenyl)acrylate (**A**) (2.00 g, 1 mmol), anhydrous K_2_CO_3_ (1.59 g, 1.2 mmol) and KI (0.79 g, 0.5 mmol) were dissolved in DMF and stirred at room temperature for 2-3 h. Then to this solution was added epichlorohydrin (**B**) (1.07 g, 1.2 mmol) and refluxed for 5–6 h. After completion of the reaction, the resulted mixture was diluted with water and extracted with ethyl acetate, the organic layer was dried over by NaSO_4_ and concentrated under vacuum. The residue was purified by silica gel chromatography with petroleum ether/ethyl acetate (8:1), concentrated eluent to give solid intermediate **C**.

### General procedure for the preparation of target compounds D1–D24

Methyl (*E*)-3-(3-methoxy-4-(oxiran-2-ylmethoxy)phenyl)acrylate (**C**) (150.00 mg, 1 mmol) and various substituted aniline (284.13 mg, 4 mmol) were dissolved in ethanol (6 mL) and refluxed for 6–8 h. Upon completion of the reaction, and an appropriate amount of water was added to the system to get white solid, the precipitate was collected by filtration. Then crude compound was subjected to column chromatography with petroleum ether/ethyl acetate (3:1) to afford target compounds **D1–D24**. Their structures were identified by ^1^H NMR, ^13^C NMR, ^19^F NMR, and HR-MS.

#### Methyl(*E*)-3-(4-(3-((4-fluoro-2-methylphenyl)amino)-2-hydroxypro poxy)-3-methoxyphenyl)acrylate (D1)

Yield 83%; Purple solid; m.p. 66–68 °C. ^1^H NMR (400 MHz, CDCl_3_) δ 7.63 (d, *J* = 15.9 Hz, 1H), 7.13–7.04 (m, 2H), 6.89 (d, *J* = 8.3 Hz, 1H), 6.85–6.76 (m, 2H), 6.59–6.54 (m, 1H), 6.33 (d, *J* = 15.9 Hz, 1H), 4.37–4.27 (m, 1H), 4.14 (ddd, *J* = 16.2, 9.7, 5.1 Hz, 2H), 3.89 (s, 3H), 3.80 (s, 3H), 3.35 (ddd, *J* = 19.3, 12.5, 5.6 Hz, 2H,), 2.16 (s, 3H). ^13^C NMR (100 MHz, CDCl_3_) δ 167.6, 155.7 (d, *J* = 235.1 Hz), 149.9, 149.6, 144.5, 142.2, 128.3, 124.7 (d, *J* = 7.2 Hz), 122.4, 117.0 (d, *J* = 22.4 Hz), 116.0, 113.7, 112.7 (d, *J* = 21.6 Hz), 110.9 (d, *J* = 7.8 Hz), 110.1, 72.2, 68.4, 55.8, 51.7, 47.0, 17.5. ^19^F NMR (376 MHz, CDCl_3_) δ − 127.92. HRMS (ESI+) m/z Calcd for C_21_H_25_FNO_5_ [M+H]^+^ 390.17113; Found 390.17090.

### Methyl(*E*)-3-(4-(3-((2,4-difluorophenyl)amino)-2-hydroxypropoxy)-3-methoxyphenyl)acrylate (D2)

Yield 80%; White solid; m.p. 69–71 °C. ^1^H NMR (400 MHz, CDCl_3_) δ 7.63 (d, *J* = 15.9 Hz, 1H), 7.14–7.01 (m, 2H), 6.88 (d, *J* = 8.2 Hz, 1H), 6.83–6.64 (m, 3H), 6.32 (d, *J* = 15.9 Hz, 1H), 4.33–4.26 (m, 1H), 4.13 (ddd, *J* = 16.0, 9.7, 5.0 Hz, 2H), 3.89 (s, 3H), 3.80 (s, 3H), 3.37 (ddd, *J* = 19.2, 12.8, 5.5 Hz, 2H). ^13^C NMR (100 MHz, CDCl_3_) δ 167.6, 154.5 (dd, *J* = 238.1, 11.1 Hz), 151.2 (dd, *J* = 242.3, 11.7 Hz), 149.9, 149.6, 144.5, 133.1 (dd, *J* = 11.7, 2.9 Hz), 128.3, 122.3, 116.0, 113.6, 112.5 (dd, *J* = 8.8, 4.4 Hz), 110.6 (dd, *J* = 21.6, 3.8 Hz), 110.1, 103.5 (dd, *J* = 26.6, 22.8 Hz), 72.0, 68.4, 55.7, 51.7, 46.6. ^19^F NMR (376 MHz, CDCl_3_) δ − 125.26, − 131.24. HRMS (ESI+) m/z Calcd for C_20_H_22_F_2_NO_5_ [M+H]^+^ 394.14606; Found 394.14606.

#### Methyl(*E*)-3-(4-(3-((2-chloro-4-fluorophenyl)amino)-2-hydroxypro poxy)-3-methoxyphenyl)acrylate (D3)

Yield 83%; White solid; m.p. 71–73 °C. ^1^H NMR (400 MHz, CDCl_3_) δ 7.63 (d, *J* = 16.0 Hz, 1H), 7.14–7.03 (m, 3H), 6.93–6.84 (m, 2H), 6.66 (dd, *J* = 9.0, 5.0 Hz, 1H), 6.33 (d, *J* = 15.9 Hz, 1H), 4.30 (dq, *J* = 6.6, 4.4 Hz, 1H), 4.13 (qd, *J* = 9.7, 5.1 Hz, 2H), 3.89 (s, 3H), 3.80 (s, 3H), 3.38 (ddd, *J* = 19.6, 12.8, 5.7 Hz, 2H). ^13^C NMR (100 MHz, CDCl_3_) δ 167.6, 154.6 (d, *J* = 238.4 Hz), 149.9, 149.6, 144.5, 140.7 (d, *J* = 2.3 Hz), 128.4, 122.4, 119.5 (d, *J* = 10.2 Hz), 116.6, 116.4, 116.0, 114.4, 114.2, 113.8, 111.7 (d, *J* = 8.0 Hz), 71.8, 68.4, 55.8, 51.7, 46.5. ^19^F NMR (376 MHz, CDCl_3_) δ − 126.52. HRMS (ESI+) m/z Calcd for C_20_H_22_FClNO_5_ [M+H]^+^ 410.11651; Found 410.11646.

#### Methyl(*E*)-3-(4-(3-((5-fluoro-2-methylphenyl)amino)-2-hydroxypropoxy)-3-methoxyphenyl)acrylate (D4)

Yield 60%; White solid; m.p. 104–105 °C. ^1^H NMR (400 MHz, CDCl_3_) δ 7.63 (d, *J* = 16.0 Hz, 1H,), 7.11–7.04 (m, 2H), 6.96 (t, *J* = 7.5 Hz, 1H), 6.89 (d, *J* = 8.2 Hz, 1H), 6.39–6.30 (m, 3H), 4.32 (ddd, *J* = 10.3, 6.5, 4.0 Hz, 1H), 4.13 (ddd, *J* = 16.1, 9.7, 5.1 Hz, 2H), 3.89 (s, 3H), 3.80 (s, 3H), 3.36 (ddd, *J* = 19.6, 12.8, 5.6 Hz, 2H), 2.10 (s, 3H). ^13^C NMR (100 MHz, CDCl_3_) δ 167.5, 162.7 (d, *J* = 240.3 Hz), 149.9, 149.7, 147.3 (d, *J* = 10.4 Hz), 144.5, 130.6 (d, *J* = 9.8 Hz), 128.4, 122.3, 117.9 (d, *J* = 2.7 Hz), 116.0, 113.8, 110.1, 103.1 (d, *J* = 21.1 Hz), 97.5 (d, *J* = 26.2 Hz), 72.0, 68.3, 55.7, 51.7, 46.2, 16.8. ^19^F NMR (376 MHz, CDCl_3_) δ − 115.66. HRMS (ESI+) m/z Calcd for C_21_H_25_FNO_5_ [M+H]^+^ 390.17113; Found 390.17099.

#### Methyl(*E*)-3-(4-(2-hydroxy-3-(phenylamino)propoxy)-3-methoxyphenyl)acrylate (D5)

Yield 70%; White solid; m.p. 94–95 °C. ^1^H NMR (400 MHz, CDCl_3_) δ 7.63 (d, *J* = 15.9 Hz, 1H), 7.19 (dd, *J* = 8.4, 7.4 Hz, 2H), 7.09–7.04 (m, 2H), 6.87 (d, *J* = 8.2 Hz, 1H), 6.74 (t, *J* = 7.3 Hz, 1H), 6.69 (s, 1H), 6.67 (s, 1H), 6.32 (d, *J* = 15.9 Hz, 1H), 4.31–4.26 (m, 1H), 4.13 (ddd, *J* = 16.0, 9.7, 5.0 Hz, 2H), 3.89 (s, 3H), 3.80 (s, 3H), 3.38 (ddd, *J* = 19.2, 12.9, 5.5 Hz, 2H). ^13^C NMR (100 MHz, CDCl_3_) δ 167.6, 150.0, 149.6, 148.0, 144.6, 129.3, 128.3, 122.4, 118.1, 115.9, 113.6, 113.3, 110.1, 72.1, 68.4, 55.8, 51.7, 46.6. HRMS (ESI+) m/z Calcd for C_20_H_24_NO_5_ [M+H]^+^ 358.16490; Found 358.16492.

#### Methyl(*E*)-3-(4-(3-((4-fluoro-3-(trifluoromethyl)phenyl)amino)-2-hydroxypropoxy)-3-methoxyphenyl)acrylate (D6)

Yield 88%; White solid; m.p. 112–114 °C. ^1^H NMR (400 MHz, CDCl_3_) δ 7.63 (d, *J* = 16.0 Hz, 1H), 7.13–6.97 (m, 3H), 6.91–6.72 (m, 3H), 6.33 (d, *J* = 15.9 Hz, 1H), 4.32–4.24 (m, 1H), 4.20–4.07 (m, 2H), 3.90 (s, 3H), 3.80 (s, 3H), 3.45–3.23 (m, 2H). ^13^C NMR (100 MHz, CDCl_3_) δ 167.6, 152.3 (d, *J* = 245.3 Hz), 149.7, 149.5, 144.4, 144.4 (d, *J* = 2.1 Hz), 128.4, 122.7 (q, *J* = 273.5 Hz), 122.4, 117.6, 117.4, 117.4, 116.1, 113.5, 110.5 (d, *J* = 4.5 Hz), 110.1, 72.1, 68.2, 55.8, 51.7, 46.9. ^19^F NMR (376 MHz, CDCl_3_) δ − 61.47, − 130.02. HRMS (ESI+) m/z Calcd for C_21_H_22_F_4_NO_5_ [M+H]^+^ 444.14286; Found 444.14114.

#### Methyl(*E*)-3-(4-(3-((4-chloro-3-fluorophenyl)amino)-2-hydroxypropoxy)-3-methoxyphenyl)acrylate (D7)

Yield 89%; White solid; m.p. 124–126 °C. ^1^H NMR (400 MHz, CDCl_3_) δ 7.63 (d, *J* = 16.0 Hz, 1H), 7.15–7.03 (m, 3H), 6.87 (d, *J* = 8.2 Hz, 1H), 6.46–6.35 (m, 2H), 6.33 (d, *J* = 15.9 Hz, 1H), 4.29–4.22 (m, 1H), 4.11 (ddd, *J* = 15.8, 9.6, 5.0 Hz, 2H), 3.90 (s, 3H), 3.80 (s, 3H), 3.32 (ddd, *J* = 19.3, 12.8, 5.4 Hz, 2H). ^13^C NMR (100 MHz, CDCl_3_) δ 167.6, 158.8 (d, *J* = 245.5 Hz), 149.7, 149.6, 148.5 (d, *J* = 9.7 Hz), 144.5, 130.6 (d, *J* = 1.3 Hz), 128.4, 122.4, 116.1, 113.6, 110.1, 109.8 (d, *J* = 2.8 Hz), 108.3 (d, *J* = 18.1 Hz), 101.0, 100.7, 72.0, 68.2, 55.8, 51.7, 46.4. ^19^F NMR (376 MHz, CDCl_3_) δ − 115.00. HRMS (ESI+) m/z Calcd for C_20_H_20_FClNO_5_ [M−H]^−^ 408.10086; Found 408.10153.

#### Methyl(*E*)-3-(4-(2-hydroxy-3-((2,4,5-trifluorophenyl)amino)propoxy)-3-methoxyphenyl)acrylate (D8)

Yield 87%; White solid; m.p. 112–114 °C. ^1^H NMR (400 MHz, CDCl_3_) δ 7.63 (d, *J* = 15.9 Hz, 1H), 7.11–7.03 (m, 2H), 6.92–6.82 (m, 2H), 6.58 (dt, *J* = 12.2, 7.9 Hz, 1H), 6.33 (d, *J* = 15.9 Hz, 1H), 4.37 (s, 1H, OH), 4.27 (d, *J* = 4.1 Hz, 1H), 4.12 (ddd, *J* = 15.7, 9.6, 5.0 Hz, 2H), 3.90 (s, 3H), 3.80 (s, 3H), 3.45–3.23 (m, 2H). ^13^C NMR (100 MHz, CDCl_3_) δ 167.7, 149.8, 149.6, 147.0 (ddd, *J* = 15.4, 13.0, 3.2 Hz), 146.3 (ddd, *J* = 12.6, 10.0, 7.56 Hz), 144.6, 140.8 (ddd, *J* = 26.0, 13.9, 11.4 Hz), 133.5, 128.5, 122.4, 116.1, 113.6, 110.2, 104.8 (t, *J* = 23.3 Hz), 100.8 (dd, *J* = 23.6, 4.0 Hz), 71.7, 68.4, 55.8, 51.7, 46.5. ^19^F NMR (376 MHz, CDCl_3_) δ − 137.98, − 142.42, − 150.59. HRMS (ESI+) m/z Calcd for C_20_H_20_F_3_NO_5_K [M+K]^+^ 450.09252; Found 450.09174.

#### Methyl(*E*)-3-(4-(3-((4-bromo-3-fluorophenyl)amino)-2-hydroxypropoxy)-3-methoxyphenyl)acrylate (D9)

Yield 75%; Yellow solid; m.p. 125–127 °C. ^1^H NMR (500 MHz, CDCl_3_) δ 7.61 (d, *J* = 15.9 Hz, 1H), 7.22 (d, *J* = 8.3 Hz, 1H), 7.10–7.01 (m, 2H), 6.85 (d, *J* = 8.3 Hz, 1H), 6.41 (dd, *J* = 11.1, 2.6 Hz, 1H), 6.34–6.27 (m, 2H), 4.24 (ddd, *J* = 10.3, 6.2, 4.3 Hz, 1H), 4.15–4.03 (m, 2H), 3.88 (s, 3H), 3.79 (s, 3H), 3.41–3.19 (m, 2H). ^13^C NMR (125 MHz, CDCl_3_) δ 167.6, 159.9 (d, *J* = 244.1 Hz), 149.8, 149.7, 149.3 (d, *J* = 9.8 Hz), 144.5, 133.4, 128.5, 122.5, 116.2, 113.7, 110.5 (d, *J* = 1.8 Hz), 110.2, 100.9 (d, *J* = 26.2 Hz), 95.2 (d, *J* = 21.1 Hz), 72.1, 68.3, 55.9, 51.8, 46.4. ^19^F NMR (376 MHz, CDCl_3_) δ − 107.03. HRMS (ESI+) m/z Calcd for C_20_H_22_FBrNO_5_ [M+H]^+^ 454.06599; Found 454.06580.

#### Methyl(*E*)-3-(4-(2-hydroxy-3-((4-methoxy-2-methylphenyl)amino) propoxy)-3-methoxyphenyl)acrylate (D10)

Yield 95%; Purple solid; m.p. 64–66 °C. ^1^H NMR (400 MHz, CDCl_3_) δ 7.63 (d, *J* = 15.9 Hz, 1H), 7.11–7.04 (m, 2H), 6.89 (d, *J* = 8.3 Hz, 1H), 6.72–6.67 (m, 2H), 6.61 (d, *J* = 8.5 Hz, 1H), 6.32 (d, *J* = 15.9 Hz, 1H), 4.32 (ddd, *J* = 10.6, 6.8, 4.1 Hz, 1H), 4.13 (ddd, *J* = 16.2, 9.7, 5.1 Hz, 2H), 3.88 (s, 3H), 3.80 (s, 3H), 3.74 (s, 3H), 3.34 (ddd, *J* = 19.3, 12.6, 5.6 Hz, 2H), 2.17 (s, 3H). ^13^C NMR (100 MHz, CDCl_3_) δ 167.6, 152.1, 150.0, 149.6, 144.6, 140.1, 128.2, 124.9, 122.4, 116.9, 115.9, 113.5, 111.6, 111.5, 110.1, 72.2, 68.4, 55.8, 55.7, 51.6, 47.3, 17.7. HRMS (ESI+) m/z Calcd for C_22_H_28_NO_6_ [M+H]^+^ 402.19111; Found 402.18994.

#### Methyl(*E*)-3-(4-(3-((4-fluoro-3-nitrophenyl)amino)-2-hydroxypropoxy)-3-methoxyphenyl)acrylate (D11)

Yield 75%; Yellow solid; m.p. 136–138 °C. ^1^H NMR (400 MHz, CDCl_3_) δ 7.63 (d, *J* = 15.9 Hz, 1H), 7.26–7.23 (m, 1H), 7.11 (d, *J* = 2.2 Hz, 1H), 7.09 (d, *J* = 1.4 Hz, 1H), 7.07 (d, *J* = 1.7 Hz, 1H), 6.90 (d, *J* = 5.1 Hz, 1H), 6.89–6.85 (m, 1H), 6.34 (d, *J* = 15.9 Hz, 1H), 4.33–4.27 (m, 1H), 4.22–4.08 (m, 2H), 3.93 (s, 3H), 3.81 (s, 3H), 3.46 (dd, *J* = 12.5, 4.3 Hz, 1H), 3.31 (dd, *J* = 12.5, 6.2 Hz, 1H). ^13^C NMR (100 MHz, CDCl_3_) δ 167.5, 149.7, 149.6, 148.2 (d, *J* = 254.9 Hz), 14.82 (d, *J* = 2.3 Hz), 144.4, 137.4, 128.7, 122.3, 119.9 (d, *J* = 7.1 Hz), 118.9 (d, *J* = 22.3 Hz), 116.2, 113.8, 110.2, 108.0 (d, *J* = 3.1 Hz), 72.2, 68.1, 55.8, 51.7, 46.7. ^19^F NMR (376 MHz, CDCl_3_) δ − 132.70. HRMS (ESI+) m/z Calcd for C_20_H_22_FN_2_O_7_ [M+H]^+^ 421.14056; Found 421.14011.

#### Methyl(*E*)-3-(4-(2-hydroxy-3-((4-(trifluoromethyl)phenyl)amino)propoxy)-3-methoxyphenyl)acrylate (D12)

Yield 75%; Yellow solid; m.p. 98–100 °C. ^1^H NMR (500 MHz, CDCl_3_) δ 7.61 (d, *J* = 15.9 Hz, 1H), 7.38 (d, *J* = 8.5 Hz, 2H), 7.09–7.01 (m, 2H), 6.85 (d, *J* = 8.2 Hz, 1H), 6.64 (d, *J* = 8.5 Hz, 2H), 6.31 (d, *J* = 16.0 Hz, 1H), 4.26 (dq, *J* = 6.3, 4.4 Hz, 1H), 4.10 (ddd, *J* = 15.8, 9.6, 5.0 Hz, 2H), 3.87 (s, 3H), 3.78 (s, 3H), 3.49–3.27 (m, 2H). ^13^C NMR (125 MHz, CDCl_3_) δ 167.6, 150.7, 149.9, 149.6, 144.5, 128.5, 126.7, 126.7, 124.9 (q, *J* = 270.2 Hz), 122.5, 119.4, 119.1, 116.2, 113.7, 112.3, 110.2, 72.1, 68.4, 55.9, 51.8, 46.0. ^19^F NMR (376 MHz, CDCl_3_) δ − 60.91. HRMS (ESI+) m/z Calcd for C_21_H_23_F_3_NO_5_ [M+H]^+^ 426.15228; Found 426.15213.

#### Methyl(*E*)-3-(4-(3-((4-fluoro-3-methylphenyl)amino)-2-hydroxypropoxy)-3-methoxyphenyl)acrylate (D13)

Yield 92%; White solid; m.p. 121–123 °C. ^1^H NMR (400 MHz, CDCl_3_) δ 7.63 (d, *J* = 15.8 Hz, 1H), 7.15–7.02 (m, 2H), 6.93–6.79 (m, 2H), 6.47 (d, *J* = 14.9 Hz, 2H), 6.32 (d, *J* = 16.0 Hz, 1H), 4.27 (s, 1H), 4.19–4.05 (m, 2H), 3.90 (s, 3H), 3.80 (s, 3H), 3.43–3.21 (m, 2H), 2.20 (s, 3H). ^13^C NMR (100 MHz, CDCl_3_) δ 167.6, 154.8 (d, *J* = 234.8 Hz), 149.9, 149.6, 144.5, 144.1, 128.3, 125.3 (d, *J* = 18.2 Hz), 122.4, δ 116.0 (d, *J* = 7.7 Hz), 115.4, 115.2, 113.6, 111.7 (d, *J* = 7.5 Hz), 110.1, 72.1, 68.4, 55.8, 51.7, 47.3, 14.8. ^19^F NMR (376 MHz, CDCl_3_) δ − 131.50. HRMS (ESI+) m/z Calcd for C_21_H_25_FNO_5_ [M+H]^+^ 390.17113; Found 390.16953.

#### Methyl(*E*)-3-(4-(3-((3,5-difluorophenyl)amino)-2-hydroxypropoxy)-3-methoxyphenyl)acrylate (D14)

Yield 93%; White solid; m.p. 107–109 °C. ^1^H NMR (400 MHz, CDCl_3_) δ 7.63 (d, *J* = 16.0 Hz, 1H), 7.15–7.02 (m, 2H), 6.87 (d, *J* = 8.2 Hz, 1H), 6.33 (d, *J* = 15.9 Hz, 1H), 6.20–6.10 (m, 3H), 4.59 (s, 1H, OH), 4.33–4.22 (m, 1H), 4.10 (ddd, *J* = 15.8, 9.6, 4.9 Hz, 2H), 3.90 (s, 3H), 3.80 (s, 3H), 3.33 (ddd, *J* = 19.3, 12.8, 5.1 Hz, 2H). ^13^C NMR (100 MHz, CDCl_3_) δ 167.6, 164.2 (d, *J* = 244.1 Hz), 164.0 (d, *J* = 244.1 Hz), 150.4 (d, *J* = 26.5 Hz), 149.7, 149.5, 144.5, 128.4, 122.4, 116.1, 113.5, 110.1, 95.7 (d, *J* = 28.6 Hz), 92.8 (d, *J* = 26.2 Hz), 92.4, 71.9, 68.2, 55.8, 51.7, 46.2. ^19^F NMR (376 MHz, CDCl_3_) δ − 110.28. HRMS (ESI+) m/z Calcd for C_20_H_20_F_2_NO_5_ [M−H]^−^ 392.13041; Found 392.13141.

#### Methyl(*E*)-3-(4-(3-((2-fluoro-5-methylphenyl)amino)-2-hydroxypropoxy)-3-meoxyphenyl)acrylate (D15)

Yield 92%; White solid; m.p. 95–97 °C. ^1^H NMR (400 MHz, CDCl_3_) δ 7.62 (d, *J* = 15.9 Hz, 1H), 7.09–7.01 (m, 2H), 6.90–6.80 (m, 2H), 6.55 (dd, *J* = 8.4, 1.6 Hz, 1H), 6.47–6.40 (m, 1H), 6.31 (d, *J* = 15.9 Hz, 1H), 4.35–4.24 (m, 1H), 4.11 (ddd, *J* = 15.8, 9.7, 5.0 Hz, 2H), 3.88 (s, 3H), 3.80 (s, 3H), 3.39 (ddd, *J* = 19.4, 13.0, 5.6 Hz, 2H), 2.23 (s, 3H). ^13^C NMR (100 MHz, CDCl_3_) δ 167.6, 150.1 (d, *J* = 236.2 Hz), 149.9, 149.6, 144.6, 136.0 (d, *J* = 11.7 Hz), 134.0 (d, *J* = 3.4 Hz), 128.2, 122.4, 117.4 (d, *J* = 6.8 Hz), 115.9, 114.1 (d, *J* = 18.5 Hz), 113.4, 113.1 (d, *J* = 2.9 Hz), 110.1, 71.7, 68.4, 55.7, 51.6, 46.1, 21.1. ^19^F NMR (376 MHz, CDCl_3_) δ − 140.64. HRMS (ESI+) m/z Calcd for C_21_H_24_FNO_5_K [M+K]^+^ 428.12701; Found 428.12668.

#### Methyl(*E*)-3-(4-(3-((4-chlorophenyl)amino)-2-hydroxypropoxy)-3-methoxyphenyl)acrylate (D16)

Yield 90%; White solid; m.p. 98–100 °C. ^1^H NMR (500 MHz, CDCl_3_) δ 7.61 (d, *J* = 15.9 Hz, 1H), 7.04 (m, *J* = 4.2, 1.7 Hz, 3H), 6.84 (d, *J* = 8.3 Hz, 1H), 6.64 (ddd, *J* = 20.5, 5.7, 1.6 Hz, 2H), 6.55–6.47 (m, 1H), 6.30 (d, *J* = 15.9 Hz, 1H), 4.25 (dq, *J* = 6.2, 4.4 Hz, 1H), 4.09 (ddd, *J* = 15.8, 9.6, 5.0 Hz, 2H), 3.87 (s, 3H), 3.78 (s, 3H), 3.33 (ddd, *J* = 19.3, 12.8, 5.5 Hz, 2H). ^13^C NMR (125 MHz, CDCl_3_) δ 167.7, 149.9, 149.6, 149.4, 144.6, 135.1, 130.3, 128.4, 122.5, 117.7, 116.1, 113.6, 112.8, 111.6, 110.2, 72.1, 68.3, 55.9, 51.8, 46.4. HRMS (ESI+) m/z Calcd for C_20_H_23_ClNO_5_ [M+H]^+^ 392.12593; Found 392.12589.

#### Methyl(*E*)-3-(4-(2-hydroxy-3-((4-isopropylphenyl)amino)propoxy)-3-methoxyphenyl)acrylate (D17)

Yield 82%; White solid; m.p. 84–86 °C. ^1^H NMR (400 MHz, CDCl_3_) δ 7.62 (d, *J* = 15.9 Hz, 1H), 7.15–6.98 (m, 3H), 6.87 (d, *J* = 8.2 Hz, 1H), 6.63 (d, *J* = 7.6 Hz, 1H), 6.55 (s, 1H), 6.50 (dd, *J* = 7.9, 1.7 Hz, 1H), 6.32 (d, *J* = 15.9 Hz, 1H), 4.28 (dt, *J* = 6.2, 5.4 Hz, 1H), 4.12 (ddd, *J* = 15.9, 9.7, 5.0 Hz, 2H), 3.88 (s, 3H), 3.80 (s, 3H), 3.38 (ddd, *J* = 19.2, 12.8, 5.5 Hz, 2H), 2.80 (dt, *J* = 13.8, 6.9 Hz, 1H), 1.22 (s, 3H), 1.21 (s, 3H). ^13^C NMR (100 MHz, CDCl_3_) δ 167.6, 150.2, 149.5, 148.1, 144.61, 129.2, 128.1, 122.4, 116.3, 115.9, 113.4, 111.7, 110.7, 110.1, 72.0, 68.4, 55.8, 51.6, 46.6, 34.2, 23.9. HRMS (ESI+) m/z Calcd for C_23_H_30_NO_5_ [M+H]^+^ 400.21185; Found 400.21164.

#### Methyl(*E*)-3-(4-(3-((4-bromo-2-methylphenyl)amino)-2-hydroxypropoxy)-3-methoxyphenyl)acrylate (D18)

Yield 83%; White solid; m.p. 100–101 °C. ^1^H NMR (400 MHz, CDCl_3_) δ 7.62 (d, *J* = 15.9 Hz, 1H), 7.22–7.14 (m, 2H), 7.11–7.02 (m, 2H), 6.87 (d, *J* = 8.2 Hz, 1H), 6.51 (d, *J* = 8.5 Hz, 1H), 6.32 (d, *J* = 15.9 Hz, 1H), 4.37–4.26 (m, 1H), 4.11 (ddd, *J* = 16.1, 9.7, 5.1 Hz, 2H), 3.87 (s, 3H), 3.80 (s, 3H), 3.35 (ddd, *J* = 19.5, 12.7, 5.6 Hz, 2H), 2.12 (s, 3H). ^13^C NMR (100 MHz, CDCl_3_) δ 167.6, 149.9, 149.6, 145.1, 144.5, 132.6, 129.6, 128.4, 124.9, 122.4, 116.0, 113.7, 111.5, 110.2, 109.2, 72.1, 68.4, 55.8, 51.7, 46.4, 17.2. HRMS (ESI+) m/z Calcd for C_21_H_25_BrNO_5_ [M+H]^+^ 450.09106; Found 450.09048.

#### Methyl(*E*)-3-(4-(3-((3,4-dimethoxyphenyl)amino)-2-hydroxypropoxy)-3-methoxyphenyl)acrylate (D19)

Yield 90%; Gray solid; m.p. 101–103 °C. ^1^H NMR (400 MHz, CDCl_3_) δ 7.63 (d, *J* = 15.9 Hz, 1H), 7.11–7.04 (m, 2H), 6.88 (d, *J* = 8.3 Hz, 1H), 6.75 (d, *J* = 8.6 Hz, 1H), 6.32 (dd, *J* = 9.2, 6.7 Hz, 2H), 6.22 (dd, *J* = 8.5, 2.6 Hz, 1H), 4.31–4.26 (m, 1H), 4.13 (ddd, *J* = 16.0, 9.7, 5.0 Hz, 2H), 3.89 (s, 3H), 3.83 (s, 3H), 3.81 (s, 3H), 3.80 (s, 3H), 3.34 (ddd, *J* = 19.1, 12.6, 5.4 Hz, 2H). ^13^C NMR (100 MHz, CDCl_3_) δ 167.6, 150.0, 149.9, 149.6, 144.5, 142.8, 142.0, 128.2, 122.4, 116.0, 113.5, 112.9, 110.1, 104.1, 99.5, 72.1, 68.4, 56.6, 55.8, 55.7, 51.7, 47.5. HRMS (ESI+) m/z Calcd for C_22_H_27_NO_7_ [M+H]^+^ 418.18603; Found 418.18582.

#### Methyl(*E*)-3-(4-(3-((2-fluoro-4-iodophenyl)amino)-2-hydroxypropoxy)-3-methoxy phenyl)acrylate (D20)

Yield 84%; White solid; m.p. 105–107 °C. ^1^H NMR (400 MHz, CDCl_3_) δ 7.62 (d, *J* = 15.9 Hz, 1H), 7.30–7.21 (m, 2H), 7.10–7.01 (m, 2H), 6.86 (d, *J* = 8.2 Hz, 1H), 6.50 (t, *J* = 8.8 Hz, 1H), 6.32 (d, *J* = 15.9 Hz, 1H), 4.34–4.04 (m, 1H), δ 4.10 (ddd, *J* = 15.7, 9.6, 4.9 Hz, 2H)0.3.88 (s, 3H), 3.80 (s, 3H), 3.36 (ddd, *J* = 19.3, 13.0, 5.4 Hz, 2H). ^13^C NMR (100 MHz, CDCl_3_) δ 167.6, 151.5 (d, *J* = 244.5 Hz), 149.8, 149.6, 144.5, 136.6 (d, *J* = 11.4 Hz), 133.4 (d, *J* = 3.6 Hz), 128.4, 123.4, 123.2, 122.4, 116.0, 113.9 (d, *J* = 3.5 Hz), 113.6, 110.1, 71.8, 68.3, 55.8, 51.7, 45.9. ^19^F NMR (376 MHz, CDCl_3_) δ − 133.02. HRMS (ESI+) m/z Calcd for C_20_H_22_FINO_5_ [M+H]^+^ 502.05212; Found 502.05151.

#### Methyl(*E*)-3-(4-(3-((3-chloro-2-methylphenyl)amino)-2-hydroxypropoxy)-3-methoxyphenyl)acrylate (D21)

Yield 92%; Pink solid; m.p. 77–78 °C. ^1^H NMR (400 MHz, CDCl_3_) δ 7.63 (d, *J* = 16.0 Hz, 1H), 7.12–6.98 (m, 3H), 6.88 (d, *J* = 8.3 Hz, 1H), 6.79 (d, *J* = 7.5 Hz, 1H), 6.55 (d, *J* = 8.0 Hz, 1H), 6.32 (d, *J* = 15.9 Hz, 1H), 4.33 (s, 1H, OH), 4.13 (ddd, *J* = 16.2, 9.7, 5.1 Hz, 3H, CH_2_, CH), 3.89 (s, 3H), 3.80 (s, 3H), 3.38 (ddd, *J* = 19.4, 12.7, 5.6 Hz, 2H), 2.23 (s, 3H). ^13^C NMR (100 MHz, CDCl_3_) δ 167.6, 149.9, 149.6, 147.2, 144.5, 134.6, 128.4, 127.1, 122.4, 120.4, 118.5, 116.0, 113.7, 110.1, 108.5, 72.1, 68.3, 55.8, 51.7, 46.5, 13.5. HRMS (ESI+) m/z Calcd for C_21_H_24_ClNO_5_Na [M+Na]^+^ 428.12352; Found 428.12292.

#### Methyl(*E*)-3-(4-(3-((3,4-difluorophenyl)amino)-2-hydroxypropoxy)-3-methoxyphenyl)acrylate (D22)

Yield 71%; White solid; m.p. 87–89 °C. ^1^H NMR (400 MHz, CDCl_3_) δ 7.63 (d, *J* = 16.0 Hz, 1H), 7.12–7.04 (m, 2H), 6.93 (ddd, *J* = 30.9, 16.0, 8.6 Hz, 2H), 6.46 (ddd, *J* = 12.7, 6.7, 2.8 Hz, 1H), 6.38–6.27 (m, 2H), 4.30–4.22 (m, 1H), 4.11 (ddd, *J* = 15.9, 9.6, 5.0 Hz, 2H), 3.90 (s, 3H), 3.80 (s, 3H), 3.30 (ddd, *J* = 19.1, 12.7, 5.4 Hz, 2H). ^13^C NMR (100 MHz, CDCl_3_) δ 167.6, 150.9 (d, *J* = 245.0 Hz), 149.8, 149.5, 145.2 (dd, *J* = 8.5, 2.0 Hz), 144.5, 143.2 (d, *J* = 249.9 Hz), 128.4, 122.4, 117.4 (dd, *J* = 18.0, 1.7 Hz), 116.1, 113.5, 110.1, 108.4 (dd, *J* = 5.5, 3.1 Hz), 101.8 (d, *J* = 20.8 Hz), 72.0, 68.3, 55.8, 51.7, 46.9. ^19^F NMR (376 MHz, CDCl_3_) δ − 137.12, − 152.50. HRMS (ESI+) m/z Calcd for C_20_H_21_F_2_NO_5_Na [M+Na]^+^ 416.12800; Found 416.12744.

#### Methyl(*E*)-3-(4-(3-((4-chloro-2-methylphenyl)amino)-2-hydroxypropoxy)-3-methoxyphenyl)acrylate (D23)

Yield 81%; White solid; m.p. 99–101 °C. ^1^H NMR (400 MHz, CDCl_3_) δ 7.62 (d, *J* = 15.9 Hz, 1H), 7.13–6.98 (m, 4H), 6.87 (d, *J* = 8.3 Hz, 1H), 6.55 (d, *J* = 8.5 Hz, 1H), 6.32 (d, *J* = 15.9 Hz, 1H), 4.31 (ddd, *J* = 10.7, 6.7, 4.2 Hz, 1H), 4.11 (ddd, *J* = 16.1, 9.7, 5.1 Hz, 2H), 3.87 (s, 3H), 3.80 (s, 3H), 3.35 (ddd, *J* = 19.5, 12.7, 5.6 Hz, 2H), 2.12 (s, 3H). ^13^C NMR (100 MHz, CDCl_3_) δ 167.6, 149.9, 149.6, 144.6, 144.5, 129.8, 128.3, 126.6, 124.4, 122.4, 122.0, 116.0, 113.6, 111.1, 110.2, 72.09, 68.4, 55.8, 51.7, 46.5, 17.3. HRMS (ESI+) m/z Calcd for C_21_H_24_ClNO_5_K [M+K]^+^ 444.09746; Found 444.09735.

#### Methyl(*E*)-3-(4-(3-((3-chloro-4-fluorophenyl)amino)-2-hydroxypropoxy)-3-methoxyphenyl)acrylate (D24)

Yield 93%; White solid; m.p. 123–125 °C. ^1^H NMR (400 MHz, CDCl_3_) δ 7.63 (d, *J* = 15.9 Hz, 1H), 7.14–7.02 (m, 2H), 6.99–6.83 (m, 2H), 6.66 (dd, *J* = 6.1, 2.8 Hz, 1H), 6.56–6.42 (m, 1H), 6.32 (d, *J* = 15.9 Hz, 1H), 4.26 (dq, *J* = 6.3, 4.3 Hz, 1H), 4.11 (ddd, *J* = 15.9, 9.6, 5.0 Hz, 2H), 3.90 (s, 3H), 3.80 (s, 3H), 3.30 (ddd, *J* = 19.1, 12.6, 5.4 Hz, 2H). ^13^C NMR (100 MHz, CDCl_3_) δ 167.6, 151.1 (d, *J* = 238.2 Hz), 149.8, 149.5, 145.1 (d, *J* = 2.1 Hz), 144.5, 128.4, 122.4, 121.0 (d, *J* = 18.5 Hz), 116.8 (d, *J* = 22.0 Hz), 116.0, 114.2, 113.5, 112.6 (d, *J* = 6.3 Hz), 110.1, 72.0, 68.2, 55.8, 51.7, 46.9. ^19^F NMR (376 MHz, CDCl_3_) δ -130.63. HRMS (ESI+) m/z Calcd for C_20_H_21_FClNO_5_K [M+K]^+^ 448.07239; Found 448.07123.

### Biological activity test method

#### In vitro antibacterial activity test

The turbidity method was used to evaluate the in vitro antibacterial activity of all target compounds against *Xoo* and *Xac* [15, 39]. Dimethyl sulfoxide (DMSO) was used as a negative control, and the commercial bactericides thiodiazole copper (**TC**) and bismerthiozol (**BT**) were used as positive controls. Add 4 mL nutrient broth (NB) medium, 1 mL test compound or commercial bactericides (the final concentration of the solution is 100 and 50 μg/mL), and 40 μL *Xoo* or *Xac* bacterial solution into the 15 mL test tube. Test the EC_50_ value of the target compounds when the concentration was 100, 50, 25, 12.5, 6.25 µg/mL, respectively. Then incubated the above sample solution in a shaker (180 rpm, 28 ± 1 °C) for about 24–48 h, until the negative control grew to the logarithmic phase. Measure the optical density at 595 nm (OD_595_) with a microplate reader (turbidity correction value = OD_Value of medium containing bacteria_ − OD_Medium value without bacteria_), and the calculation formula for the inhibition rate I was: *I* = (*C − T*)*/C* × 100%. *C* represented the corrected absorbance value of the untreated NB medium, and *T* represented the corrected absorbance value of the treated NB medium. Each experiment was tested for three times.

#### In vivo antibacterial activity test

The curative and protective activities in potted plants of compound **D24** against rice bacterial leaf blight were determined by Schaad’s method [40, 41]. Dimethyl sulfoxide (DMSO) was used as a negative control, and the commercial agricultural antibacterial agents thiodiazole copper (**TC**) and bismerthiozol (**BT**) were used as positive controls. Under greenhouse control conditions, the curative activity of compound **D24** against rice bacterial leaf blight was determined. Inoculate rice leaves with *Xoo* that has grown to the logarithmic growth stage. Rice leaves were inoculated with *Xoo* which had reached logarithmic growth stage. One day after inoculation, 200 μg/mL of compound **D24** solution was evenly sprayed on the rice leaves, and distilled water containing DMSO was evenly sprayed on the plants. Then they were placed in a plant growth room (28 °C and 90% RH) for 14 days to determine the disease index of rice leaves. Similarly, compound **D24** had protective activity against rice bacterial leaf blight. 200 μg/mL compound **D24** solution was evenly sprayed on rice leaves, spray distilled water containing DMSO was evenly sprayed on the plants. One day after spraying, the rice leaves were inoculated with *Xoo* that had grown to the logarithmic growth stage. Then they were placed in a plant growth room (28 °C and 90% RH) for 14 days to determine the disease index of rice leaves. The control efficiency of compound **D24**’s curative and protective activities *I (%)* = (*C − T*)/*C* × *100*%, where *C* was the disease index of the negative control group, and *T* was the disease index of the treatment group.

#### In vivo anti-TMV activity test

The inhibitory effect of the target compounds on TMV was tested by literature method [42]. Before the test, tobacco leaves with the same size, shape and age of left and right leaves were selected. The curative activity test was to wash and dry the tobacco 30 min after inoculation with TMV virus, and then apply the prepared target compound solution on the left side of the tobacco and the blank control solvent on the right side. The protective activity test was to smear the prepared target compound solution on the left side of tobacco leaves and the blank control solution on the right side of tobacco leaves, inoculate TMV virus after 20–22 h, wash it with clean water after 30 min, and then dry it naturally. The inactivation activity was to evenly mix the same volume of virus solution with the drug solution of the target compound for 30 min and then apply it on the left side of the tobacco leaf, apply the mixed solution of equal volume of virus solution and blank solvent on the right side of the tobacco leaf as the control, and rinse it with clean water after 30 min. The activity of the compounds was calculated using the following formula. Inhibition rate *I (%)* = (*C*_*av*_* − T*_*av*_)/*C*_*av*_ × *100%*, where *C* is the number of lesions in leaves without compound treatment, *T* is the number of lesions in leaves treated with compound, and *av* is the average of the number of lesions.

## Results and discussion

### Antibacterial activity in vitro screening of target compounds

On the basis of previous work, the antibacterial activity of the target compounds was tested by turbidity method [15, 16, 39]. The preliminary results of the in vitro antibacterial activities of target compounds **D1–D24** against *Xoo* and *Xac* are shown in Table 1. Some compounds showed moderate antibacterial activity. Compound **D24** showed good inhibitory activity against *Xoo* (90.7% and 80.6% at concentrations of 100 and 50 μg/mL, respectively), similar to **BT** (90.1% and 80.2%, respectively). The antibacterial activity of compounds **D3, D5, D7, D22,** and **D23** against *Xoo* was higher than that of **TC** (65.7% and 46.9% at concentrations of 100 and 50 μg/mL, respectively). Compounds **D2, D3, D7, D11,** and **D15** had moderate antibacterial activity against *Xac* at 100 μg/mL, with the inhibitory activity of compounds **D3, D7** and **D11** slightly higher than that of **BT** (which was 64.6%). To further quantify the antibacterial activity, the concentration values for 50% of maximal effect (EC_50_) were determined for select compounds (Table 2). The EC_50_ values of compounds **D22** and **D24** on *Xoo* were 14.5 and 16.2 μg/mL, respectively, which were better than that of **TC** (44.5 μg/mL) and similar to that of **BT** (16.2 μg/mL). Compounds **D3, D7** and **D11** had inhibitory effects on *Xac*, and their EC_50_ values (37.3, 29.4 and 45.6 μg/mL, respectively) were slightly better than the EC_50_ of **BT** (46.8 μg/mL).

**Table 1:** In vitro antibacterial activity of the target compounds against *Xoo* and *Xac*

Compd	*Xoo*		*Xac*	
	Inhibition rate (%)		Inhibition rate (%)	
	100 μg/mL	50 μg/mL	100 μg/mL	50 μg/mL
**D1**	26.5 ± 1.9	21.8 ± 1.2	47.4 ± 1.9	32.1 ± 1.8
**D2**	35.6 ± 2.1	31.8 ± 3.8	55.3 ± 0.5	45.9 ± 3.3
**D3**	77.8 ± 2.8	66.4 ± 2.7	68.2 ± 2.7	48.7 ± 1.9
**D4**	28.2 ± 2.7	28.0 ± 1.1	25.2 ± 0.2	15.7 ± 2.6
**D5**	72.0 ± 1.3	55.0 ± 1.2	22.5 ± 3.7	21.4 ± 0.9
**D6**	35.2 ± 0.9	17.1 ± 3.0	29.7 ± 4.5	22.4 ± 4.7
**D7**	66.5 ± 1.8	47.9 ± 1.0	74.5 ± 2.3	60.0 ± 2.5
**D8**	25.3 ± 1.8	19.9 ± 2.2	31.9 ± 3.5	27.8 ± 2.6
**D9**	47.4 ± 1.3	32.8 ± 2.9	36.2 ± 2.2	12.0 ± 4.8
**D10**	26.8 ± 3.6	23.6 ± 2.7	33.1 ± 2.6	29.9 ± 0.1
**D11**	33.0 ± 1.2	32.4 ± 3.6	68.5 ± 3.5	45.3 ± 1.8
**D12**	22.7 ± 1.8	22.6 ± 3.7	33.5 ± 1.0	33.0 ± 0.7
**D13**	34.5 ± 2.4	26.8 ± 1.3	28.6 ± 1.9	15.4 ± 3.1
**D14**	36.7 ± 4.4	22.9 ± 1.1	26.7 ± 4.0	12.6 ± 3.3
**D15**	43.4 ± 2.9	26.4 ± 4.0	57.9 ± 3.2	45.9 ± 1.9
**D16**	42.2 ± 0.6	26.4 ± 3.1	31.1 ± 3.9	19.8 ± 4.2
**D17**	20.6 ± 1.8	8.7 ± 2.8	28.2 ± 3.8	12.8 ± 2.6
**D18**	32.9 ± 2.1	30.0 ± 0.8	27.7 ± 4.1	26.3 ± 2.4
**D19**	24.9 ± 3.5	19.8 ± 2.0	25.1 ± 1.3	14.2 ± 4.3
**D20**	34.2 ± 0.6	5.2 ± 2.2	34.4 ± 1.6	20.9 ± 0.1
**D21**	38.6 ± 2.6	37.7 ± 1.8	26.0 ± 3.0	21.6 ± 3.0
**D22**	89.1 ± 3.7	68.0 ± 3.1	35.7 ± 3.5	24.7 ± 2.5
**D23**	79.9 ± 1.0	63.6 ± 3.6	28.5 ± 2.2	23.1 ± 3.5
**D24**	90.7 ± 0.8	80.5 ± 2.7	25.9 ± 1.8	21.7 ± 3.7
**BT**^**a**^	90.1 ± 2.3	80.2 ± 1.3	64.6 ± 1.9	51.2 ± 1.4
**TC**^**a**^	65.7 ± 0.9	46.9 ± 2.6	76.8 ± 0.7	65.2 ± 2.0

Average of three replicates

^a^The commercial agricultural antibacterial agents bismerthiazol (BT) and thiodiazole copper (TC) were used as positive control

**Table 2:** Antibacterial activities of some target compounds against *Xoo* and *Xac *in vitro

Compd	*Xoo*			*Xac*		
	Regression equation	R^2^	EC_50_ (μg/mL)	Regression equation	R^2^	EC_50_ (μg/mL)
**D1**				y = 0.54x + 3.8	0.94	122.9 ± 4.9
**D2**				y = 0.78x + 3.5	0.97	74.2 ± 3.9
**D3**	y = 0.84x + 3.9	0.99	20.3 ± 0.9	y = 0.92x + 3.5	0.96	37.3 ± 1.4
**D5**	y = 0.87x + 3.7	0.93	27.1 ± 2.5			
**D7**	y = 1.16x + 3.0	0.98	45.4 ± 1.3	y = 1.13x + 3.3	0.99	29.4 ± 4.1
**D11**				y = 0.99x + 3.3	0.93	45.6 ± 0.5
**D15**				y = 0.99x + 3.3	0.96	66.1 ± 4.7
**D22**	y = 1.33x + 3.3	0.95	16.2 ± 1.5			
**D23**	y = 1.02x + 3.6	0.94	19.5 ± 0.6			
**D24**	y = 1.46x + 3.2	0.95	14.5 ± 0.8			
**BT**^**a**^	y = 1.62x + 3.0	0.98	16.2 ± 3.4	y = 0.83x + 3.6	0.95	46.8 ± 5.0
**TC**^**a**^	y = 0.93x + 3.4	0.97	44.5 ± 3.4	y = 1.04x + 3.5	0.95	23.8 ± 4.9

Average of three replicates

^a^The commercial agricultural antibacterial agents bismerthiazol (BT) and thiodiazole copper (TC) were used as positive control

#### Antibacterial activity in vivo

**Fig. 3:**
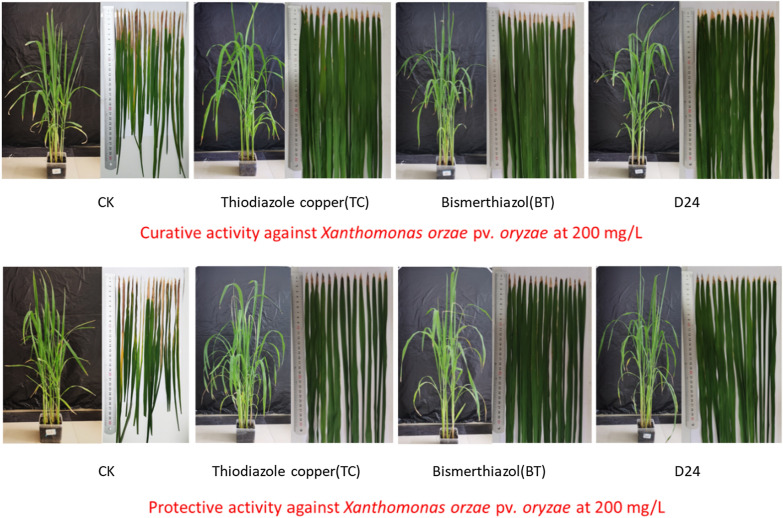
Curative and protective activities of compound D24 against rice bacterial leaf blight under greenhouse conditions at 200 μg/mL, with BT and TC as the positive control agents

Based on its promising antibacterial activity in vitro, the in vivo activity of compound **D24** against rice bacterial leaf blight at 200 μg/mL was determined, and the results are shown in Tables 3 and 4, and Fig. 3. The protective activity of **D24** was 50.1%, higher than that of **BT** (45.8%) and **TC** (43.7%). Compound **D24** also had good curative activity (50.5%), superior to that of **BT** (47.1%) and **TC** (46.1%).

**Table 3:** The protective activity of compound D24 against *Xanthomonas oryzae* pv*. oryzae *in vivo at 200 μg/mL

Treatment	14 days after spraying		
	Morbidity (%)	Disease Index (%)	Control efficiency (%)^a^
**D24**	100	42.2D	50.1A
**BT**^**b**^	100	45.8C	45.8B
**TC**^**b**^	100	47.6B	43.7C
**CK**^**c**^	100	84.6A	

^a^Statistical analysis was conducted by the analysis of variance method under the conditions of equal variances assumed (*P* > 0.05) and equal variances not assumed (*P* < 0.05). Different uppercase letters indicate the values of protection activity with significant difference among different treatment groups at *P* < 0.05

^b^Commercial bactericides bismerthiazol (BT) and thiodiazole copper (TC) were used as positive control agents

^c^Negative control

**Table 4:** The curative activity of compound D24 against *Xanthomonas oryzae* pv*. oryzae *in vivo at 200 μg/mL

Treatment	14 days after spraying		
	Morbidity (%)	Disease Index (%)	Control efficiency (%)^a^
**D24**	100	42.8C	50.5A
**BT**^**b**^	100	45.8B	47.1B
**TC**^**b**^	100	46.6B	46.1C
**CK**^**c**^	100	86.7A	

^a^Statistical analysis was conducted by the analysis of variance method under the conditions of equal variances assumed (*P* > 0.05) and equal variances not assumed (*P* < 0.05). Different uppercase letters indicate the values of protection activity with significant difference among different treatment groups at *P* < 0.05

^b^Commercial bactericides bismerthiazol (BT) and thiodiazole copper (TC) were used as positive control agents

^c^Negative control

### Anti-TMV activity in vivo screening of target compounds

The inhibitory effect of ferulic acid derivatives D1–D24 on TMV was further studied based on the method of literature and the previous work of antiviral activity test [1, 12, 42]. The bioassay results indicated that most of the compounds exhibited moderate to good anti-TMV activity at 500 μg/mL, as shown in Table 5. The curative activities of compounds **D1, D5, D12, D13, D18, D21,** and **D24** were 56.1, 59.3, 59.8, 53.9, 45.5, 74.0, and 74.1%, respectively, which were better than that of ribavirin (44.8%). Compounds **D21** and **D24** (74.0 and 74.1%, respectively) showed slightly higher curative activity than ningnanmycin (70.0%). Compounds **D3, D4, D5, D7, D9, D14, D18, D20,** and **D24** exhibited good protective activity (respectively 54.6, 52.6, 59.6, 53.1, 70.7, 74.3, 68.1, 51.9, and 54.9%), higher than ribavirin (50.0%). Compounds **D9, D14** and **D18** (70.7, 74.3 and 68.1%, respectively) showed better activity than ningnanmycin (65.3%). Most of the compounds showed excellent inactivation activity against TMV compared to ribavirin (73.5%). Notably, compound **D3** (93.7%) was slightly better than ningnanmycin (93.2%). The EC_50_ values of the inactivation activity of some compounds were tested, and the results are shown in Table 6. In particular, the EC_50_ value of compound **D3** was 38.1 μg/mL, which was higher than that of ningnanmycin (EC_50_ = 39.2 μg/mL).

**Table 5:** Antiviral activities of target compounds against TMV in vivo at 500 μg/mL^a^

Compd	Curative activity (%)	Protective activity (%)	Inactivation activity (%)
**D1**	56.1 ± 0.8	49.9 ± 3.9	76.9 ± 1.4
**D2**	31.8 ± 4.8	41.8 ± 3.5	89.1 ± 3.8
**D3**	37.5 ± 0.8	54.6 ± 2.5	93.7 ± 1.9
**D4**	38.8 ± 4.5	52.6 ± 4.5	89.5 ± 1.5
**D5**	59.3 ± 0.7	59.6 ± 4.9	72.9 ± 2.7
**D6**	25.1 ± 2.7	35.1 ± 0.1	73.0 ± 1.7
**D7**	31.8 ± 0.7	53.1 ± 0.5	74.2 ± 2.8
**D8**	40.9 ± 2.7	47.2 ± 0.8	85.2 ± 3.1
**D9**	31.5 ± 3.6	70.7 ± 5.0	84.9 ± 1.9
**D10**	38.4 ± 3.3	31.4 ± 1.1	84.3 ± 4.9
**D11**	35.4 ± 1.0	34.2 ± 0.1	74.1 ± 2.0
**D12**	59.8 ± 1.3	39.9 ± 1.1	61.2 ± 0.3
**D13**	53.9 ± 4.7	39.8 ± 4.9	71.4 ± 0.4
**D14**	21.8 ± 4.5	74.3 ± 3.7	81.2 ± 2.5
**D15**	32.3 ± 4.5	35.0 ± 2.4	82.7 ± 3.3
**D16**	22.5 ± 4.8	19.0 ± 3.7	84.9 ± 3.4
**D17**	33.6 ± 2.5	43.9 ± 2.2	82.5 ± 3.6
**D18**	45.5 ± 3.2	68.1 ± 3.3	84.1 ± 4.5
**D19**	34.6 ± 2.1	43.7 ± 3.6	85.6 ± 4.2
**D20**	42.8 ± 0.1	51.9 ± 1.6	81.8 ± 0.7
**D21**	74.0 ± 4.0	41.5 ± 2.1	60.1 ± 0.1
**D22**	20.2 ± 1.7	43.6 ± 2.5	66.8 ± 0.7
**D23**	40.2 ± 3.6	54.9 ± 2.7	74.5 ± 1.5
**D24**	74.1 ± 1.9	47.4 ± 2.6	74.2 ± 0.8
**Ribavirin**	44.8 ± 1.2	50.0 ± 1.8	73.5 ± 1.6
**Ningnanmycin**	70.0 ± 3.8	65.3 ± 2.5	93.2 ± 0.5

^a^ All active values are the average of three duplicates

**Table 6:** EC_50_ of some target compounds anti-TMV activity

Compd	Regression equation	R^2^	EC_50_ of inactivation activity^a^
**D2**	y = 1.33x + 2.6	0.99	56.8 ± 4.4
**D3**	y = 1.26x + 3.0	0.96	38.1 ± 1.4
**D4**	y = 1.29x + 2.7	0.98	52.5 ± 4.4
**D8**	y = 1.15x + 2.9	0.99	57.3 ± 2.9
**D19**	y = 1.12x + 3.0	0.99	57.9 ± 4.0
**Ningnanmycin**^**b**^	y = 1.37x + 2.8	0.99	39.2 ± 3.8

^a^Average of three replicates

^b^Ningnanmycin was used as the control

### Autodocking and MD simulation

**Fig. 4:**
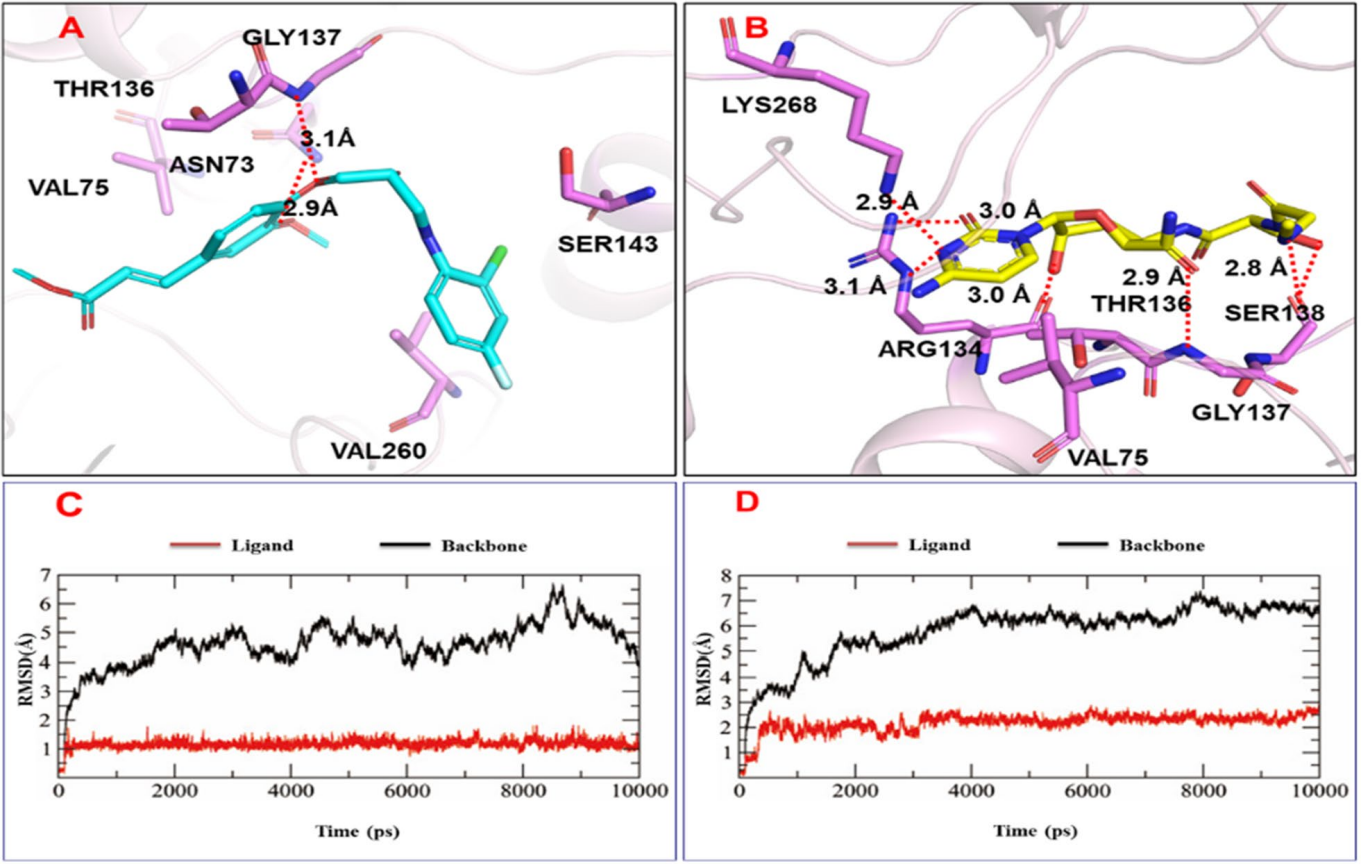
Autodocking and MD simulation studies: **A** Autodocking of compound **D3**, **B** Autodocking of ningnanmycin, **C** MD simulation of compound **D3**, **D** MD simulation of ningnanmycin

Based on previous work [43, 44], the interaction between the active target compounds and TMV coat protein (TMV-CP) (PDB 97 code: 1EI7) was investigated. The binding mode of compound **D3** and TMV-CPwas studied by molecular docking, and the results are shown in Fig. 4. Compound D3 has a strong affinity for TMV-CP with a binding energy of − 7.54 kcal/mol, which is better than that of ningnanmycin (− 6.88 kcal/mol). Binding to the active site of TMV-CP was achieved through amino acid residues that play a key role in the self-assembly of TMV-CP, including GLY137, ASN73, THR136, VAL75, SER143 and VAL260 (Fig. 4A and B). Among them, there is a strong hydrogen bond interaction between compound **D3** and key residues (GLY137 and ASN73), the bond lengths of which are 3.1 Å and 2.9 Å, respectively, and GLY137 also interacts with ningnanmycin. The carbon atoms of compound **D3** and ningnanmycin interact with THR136 and VAL75 residues through hydrophobic bonds, and a halogen bond is formed between **D3** and SER143. Therefore, compound **D3** may the same as ningnanmycin, disrupting the three-dimensional structure of TMV-CP, making TMV particles unable to self-assemble, thereby achieving antiviral effects.

Molecular dynamics (MD) simulations were used to evaluate the stability of compound **D3** and ningnanmycin. Under simulated conditions, the root-mean-square deviation (RMSD) of the atom from its initial position was measured and recorded (Fig. 4C and D). The interaction of other binding site residues affects the energy and geometric characteristics, so that the ligand obtains a stable conformation at the active site.

### Structure–activity relationship analysis

The preliminary structure–activity relationship (SAR) indicated that different substituents of ferulic acid compounds had a strong influence on their activity against *Xoo*, *Xac* and TMV. According to Table 1, the position and number of substituted fluorine atoms on the aromatic ring had a significant effect on the inhibition of *Xoo*. When the substituents are two fluorine atoms at the same time, the activity of the compounds towards *Xoo* is different: **D22** (R = 3,4-di-F-Ph) > **D14** (R = 3,5-di-F-Ph) > **D2** (R = 2,4-di-F-Ph). The introduction of chlorine atoms to the electron-withdrawing group improved the activity of the compound: **D24** (R = 3-Cl-4-F-Ph) and **D3** (R = 2-Cl-4-F-Ph) > **D13** (R = 3-CH_3_-4-F-Ph) and **D1** (R = 2-CH_3_-4-F-Ph). Compounds with weak electron-withdrawing effects at the same position had higher activity: **D23** (R = 4-Cl-2-CH_3_-Ph), **D18** (R = 4-Br-2-CH_3_-Ph) > **D1** (R = 4-F-2-CH_3_-Ph). The introduction of substituents on the benzene ring helps to improve the antibacterial activity against *Xac*: **D7** (R = 4-Cl-3-F-Ph) > **D11** (R = 3-NO_2_-4-F-Ph) > **D3** (R = 2-CH_3_-5-F-Ph) > **D15** (R = 5-CH_3_-2-F-Ph) > **D2** (R = 2,4-di-F-Ph) > **D1** (R = 2-CH_3_-4-F-Ph) > **D5** (R = Ph). Different halogens and positions influenced the activity of the compound: **D7** (R = 4-Cl-3-F-Ph) > **D9** (R = 4-Br-3-F-Ph), **D3** (R = 2-Cl-4-F-Ph) > **D24** (R = 3-Cl-4-F-Ph).

According to Table 5, increasing the number of fluorine atoms increased the curative activity against TMV, particularly in the case of 2,4 substituted difluoride: **D8** (R = 2,4,5-tri-F-Ph) > **D2** (R = 2,4-di-F-Ph) > **D14** (R = 3,5-di-F-Ph), **D22** (R = 3,4-di-F-Ph). The electron-withdrawing at the same position is more active than the electron-donating, and the compound with a strong electron-withdrawing effect were more active: **D1** (R = 4-F-2-CH_3_-Ph) > **D18** (R = 4-Br-2-CH_3_-Ph), **D23** (R = 4-Cl-2-CH_3_-Ph) > **D10** (R = 4-OCH_3_-2-CH_3_-Ph). The protective activity sequence of compounds with two electron-withdrawing substituents on the benzene ring was as follows: **D14** (R = 3,5-di-F-Ph) > **D9** (R = 4-Br-3-F-Ph) > **D3** (R = 2-Cl-4-F-Ph) > **D7** (R = 4-Cl-3-F-Ph) > **D20** (R = 4-I-2-F-Ph) > **D24** (R = 3-Cl-4-F-Ph) > **D22** (R = 3,4-di-F-Ph) > **D2** (R = 2,4-di-F-Ph) > **D6** (R = 4-F-3-CF_3_-Ph) > **D11** (R = 3-NO_2_-4-F-Ph). The electron-donating group on the benzene ring can improve the inactivation activity of the compound: **D19** (R = 3,4-di-OCH_3_-Ph) > **D10** (R = 4-OCH_3_-2-CH_3_-Ph) > **D17** (R = 4-CH(CH_3_)_2_-Ph) > **D5** (R = Ph). The same halogen introduced at different positions had different activities: **D4** (R = 5-F-2-CH_3_-Ph) > **D1** (R = 4-F-2-CH_3_-Ph), **D23** (R = 4-Cl-2-CH_3_-Ph) > **D21** (R = 3-Cl-2-CH_3_-Ph). In general, the R substituents of the compounds resulting in better anti-TMV activity or inhibition of *Xoo* and *Xac* frequently contained fluorine atoms. The introduction of fluorine atoms into compounds is known to effectively alter conformation, membrane permeability, lipophilicity, metabolic pathways, and pharmacokinetic properties, and can improve biological activity in many cases [45, 46]. However, it is also affected by other factors such as the position of the substituent and the influence of other substituents on the fluorine atom, which leads to changes in the activity of the compound.

## Conclusion

In summary, a series of ferulic acid derivatives containing an *β*-amino alcohol were designed and synthesized, and the biological activities of the target compounds were evaluated. Bioassays results showed that compound **D24** had a good inhibitory effect on *Xoo*, which was superior to the commercial bactericide **BT** and **TC**. The inhibitory effect of compound **D7** on *Xac* was also higher than **BT** and close to **TC**. As compound **D3** (EC_50_ = 39.2 μg/mL) had good passivating activity against TMV, the interaction of the ligand molecules with TMV-CP was explored by molecular docking and molecular dynamics simulations. The results of molecular docking indicated that compound **D3** was inserted into the active site of TMV-CP through amino acid residues, and had a strong affinity for TMV-CP with a binding energy of − 7.54 kcal/mol, which was superior to the commercial antiviral agent ningnanmycin (− 6.88 kcal/mol). Therefore, the three-dimensional structure of the TMV coat protein may be disrupted by the compounds D3 and ningnanmycin, preventing the TMV particles from self-assembling and thus producing a potent antiviral effect. The synthetic compounds in this work may provide potential lead compounds for the discovery of novel plant fungicides and antivirals.

## Supplementary Information

**Additional file 1.** ^1^H NMR, ^13^C NMR, ^19^F NMR, and HR-MS spectra of the title compounds D1–D24.

## Data Availability

All data generated or analyzed during this study are included in this published article (and its Additional file 1).

## Abbreviations

*Xoo*: *Xanthomonas oryzae* Pv*. oryzae*
*Xac*: *Xanthomonas axonopodis* Pv. *citri*
TMV: Tobacco mosaic virus
FA: Ferulic acid
TC: Thiodiazole copper
BT: Bismerthiazol
^1^H NMR: ^1^H nuclear magnetic resonance
^13^C NMR: ^13^C nuclear magnetic resonance
^19^F NMR: ^19^F nuclear magnetic resonance
HRMS: High-resolution mass spectrum
