# Supplementary material for: Design, synthesis, and bioactivity of ferulic acid derivatives containing an β-amino alcohol
Source: BMC Chem. 2022 May 17;16(1):34. doi: 10.1186/s13065-022-00828-8 (PMC9115944; doi:10.1186/s13065-022-00828-8)
Supplement: Supplementary file 1 — Additional file 1. 1H NMR, 13C NMR, 19F NMR, and HR-MS spectra of the title compounds D1–D24. [file 13065_2022_828_MOESM1_ESM.pdf]

Additional file 1: ^{1}H NMR, ^{13}C NMR, ^{19}F NMR, and HR-MS spectra of the title compounds **D1 - D24**.

Table of Contents

1、^{1}H NMR, ^{13}C NMR, and ^{19}F NMR spectra of the title compounds **D1 - D24**............................. 2

2、HRMS spectra of the title compounds **D1 - D24**..34

1、^{1}H NMR, ^{13}C NMR, and ^{19}F NMR spectra of the title compounds D1 - D24

D1

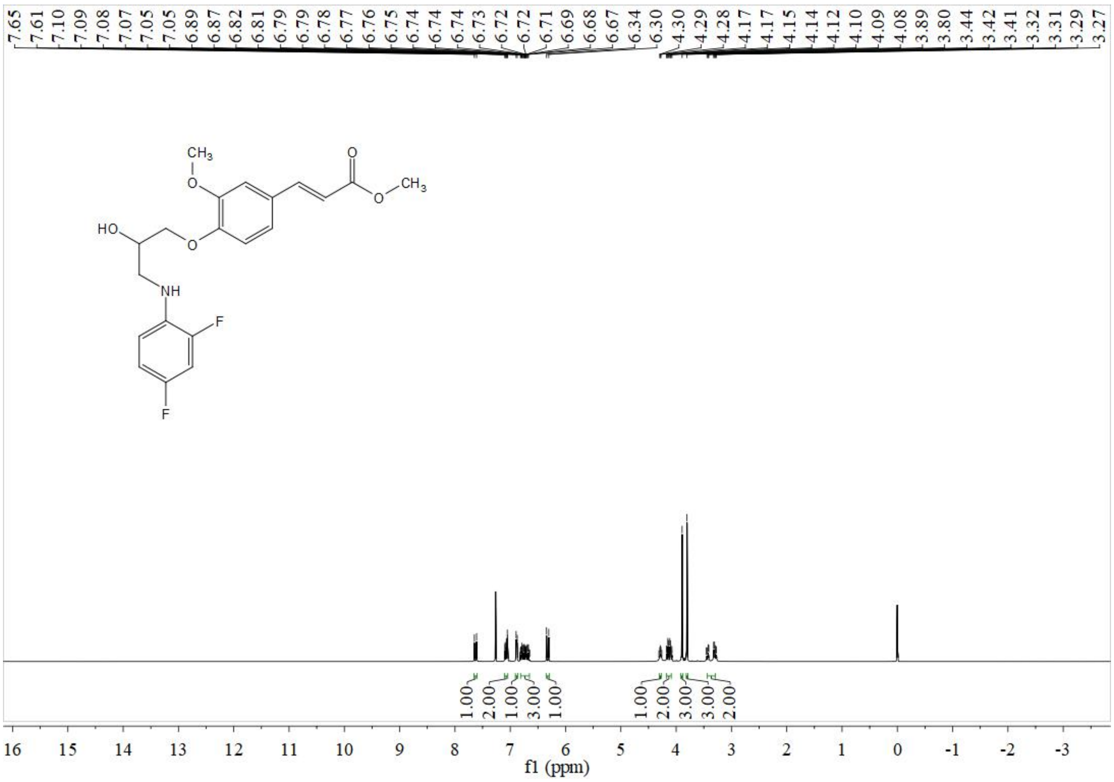

Figure S1 ^{1}H NMR Spectrum of **D1**

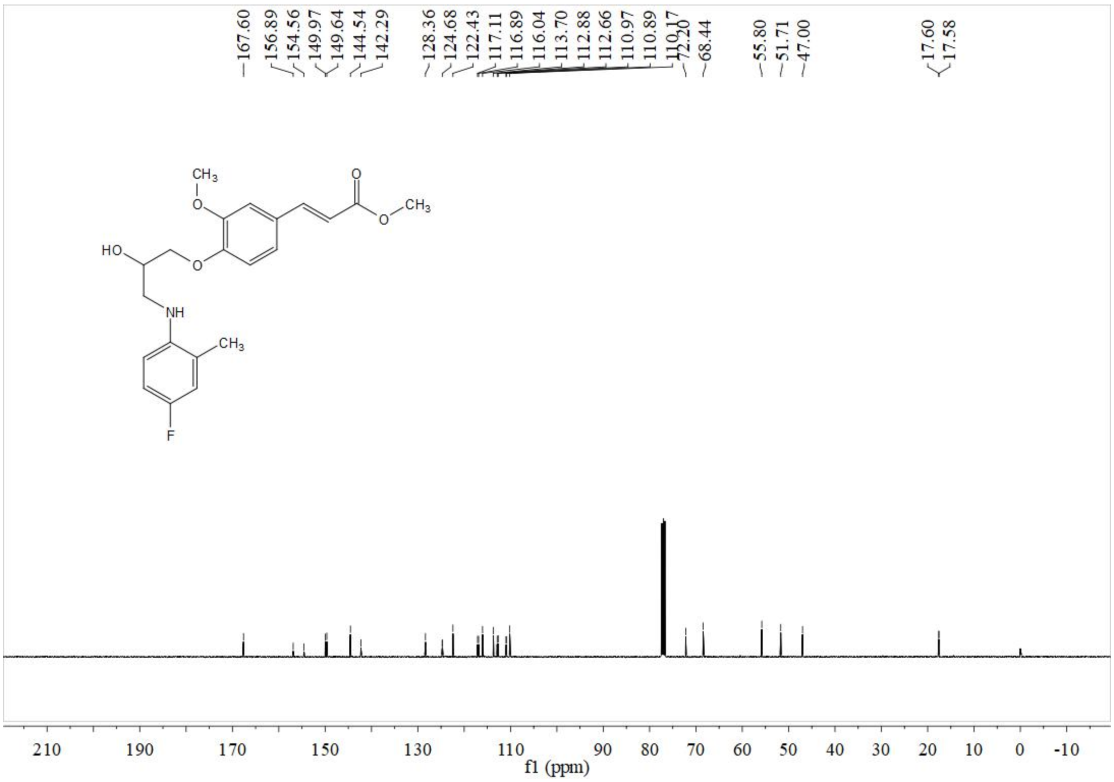

Figure S2 ^{13}C NMR Spectrum of **D1**

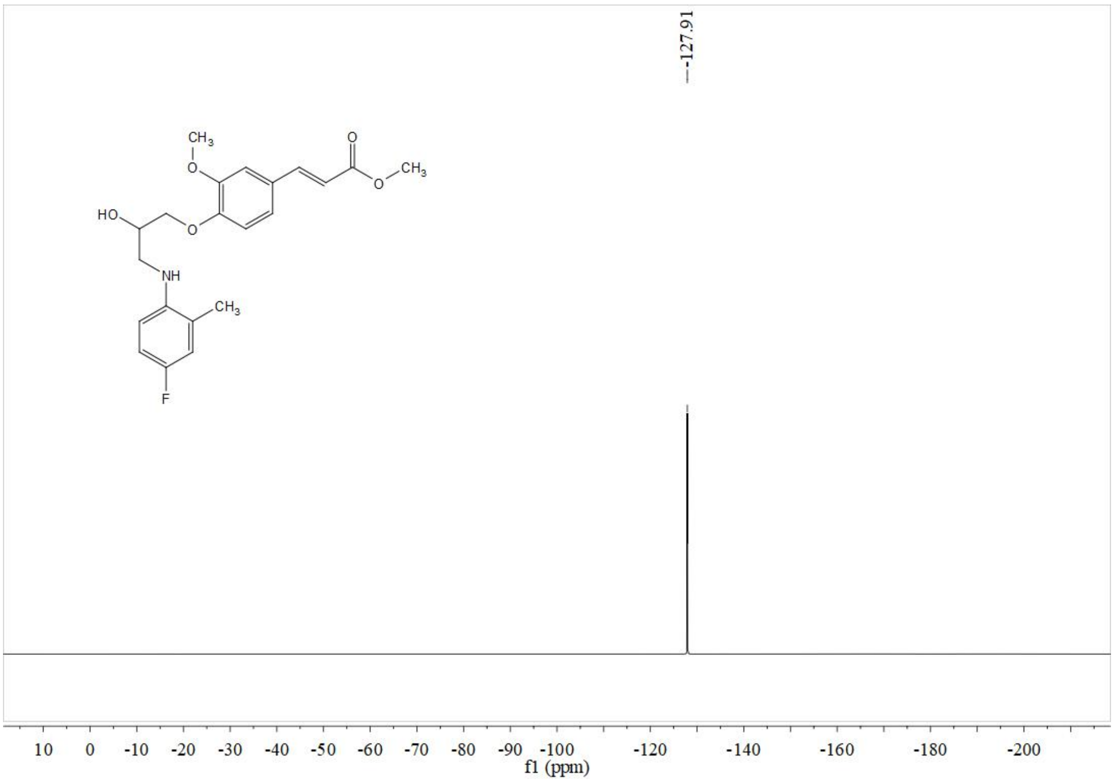

Figure S3 ^{19}F NMR Spectrum of **D1**

D2

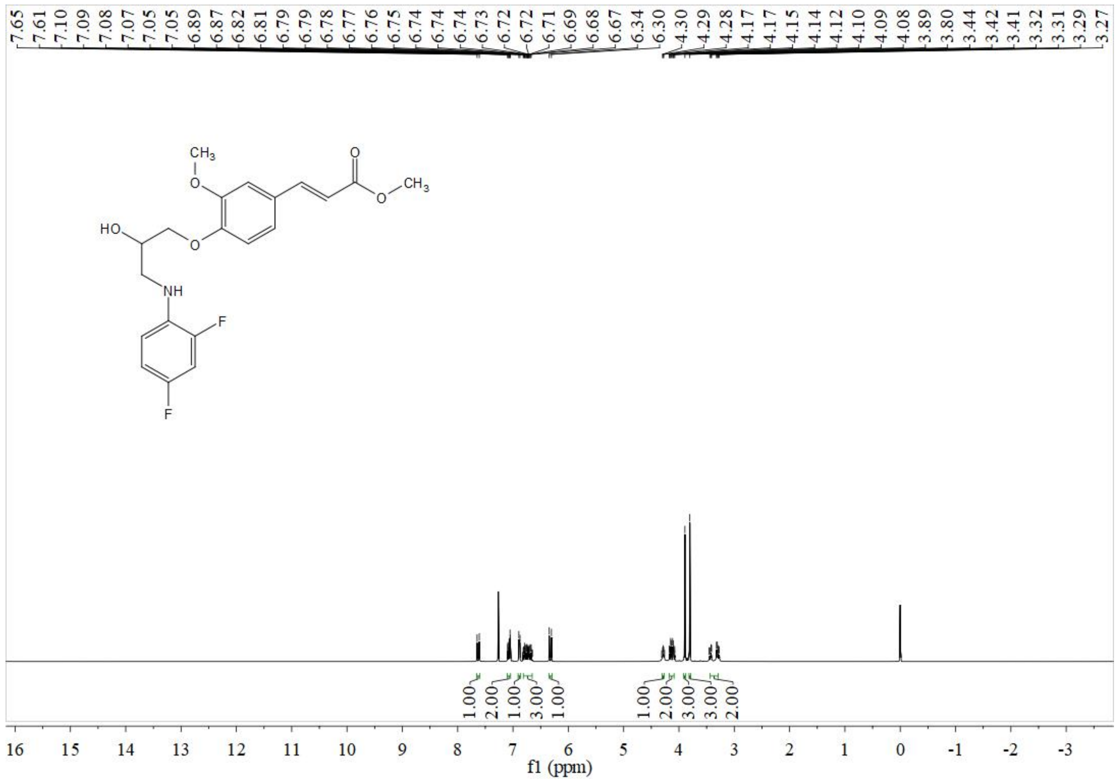

Figure S4 ^{1}H NMR Spectrum of **D2**

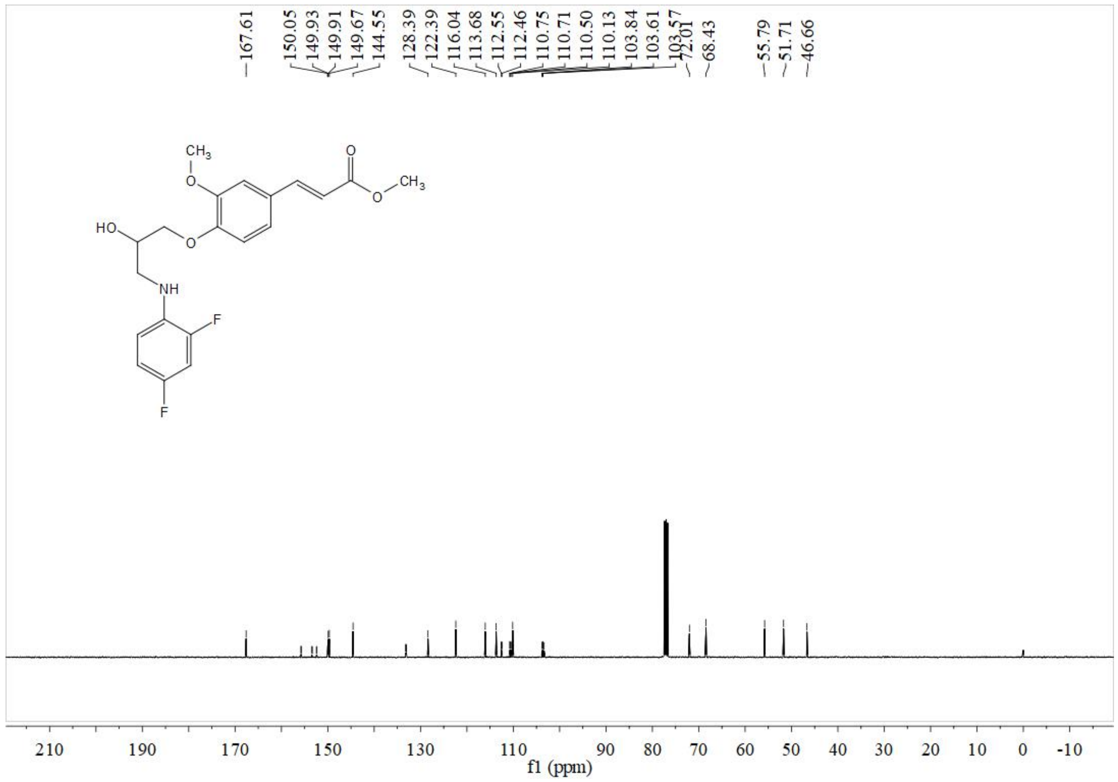

Figure S5 ^{13}C NMR Spectrum of **D2**

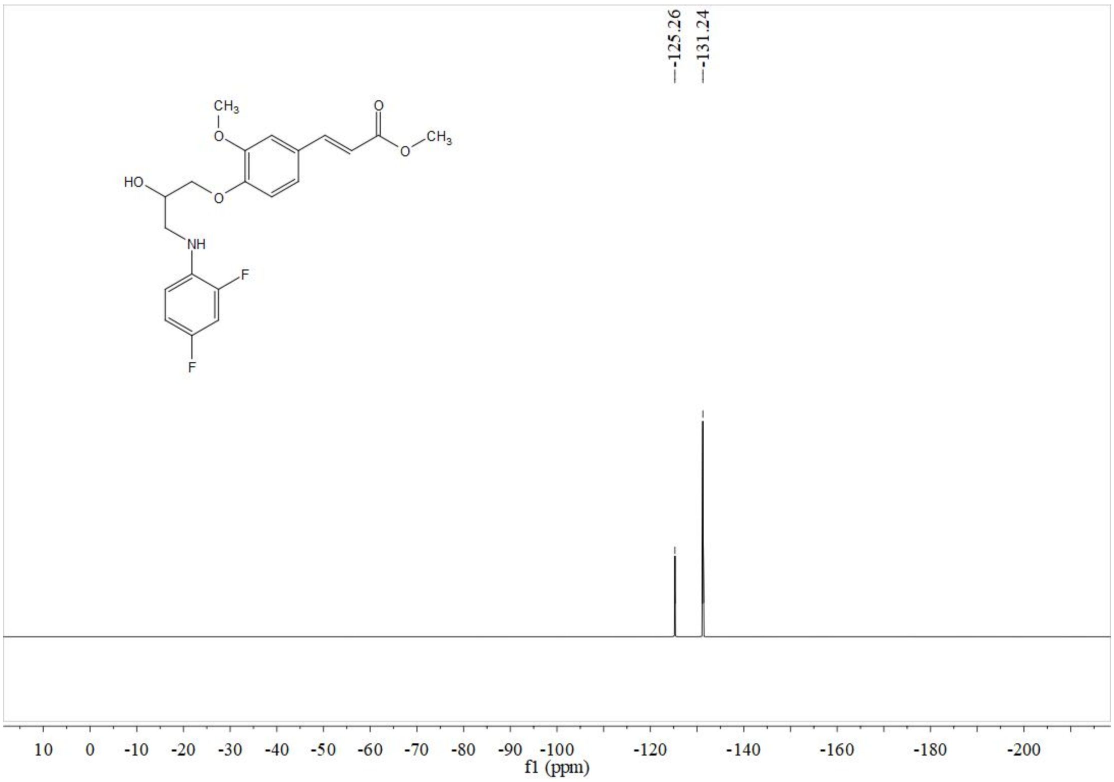

Figure S6 ^{19}F NMR Spectrum of **D2**

D3

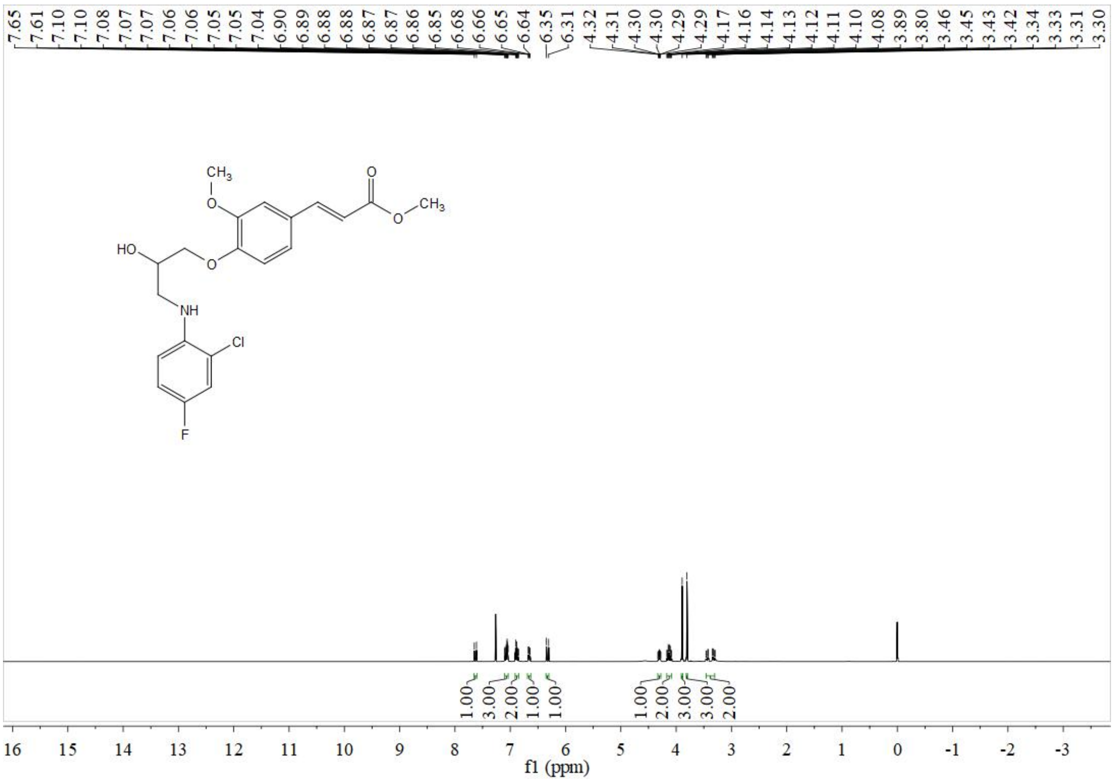

Figure S7 ^{1}H NMR Spectrum of **D3**

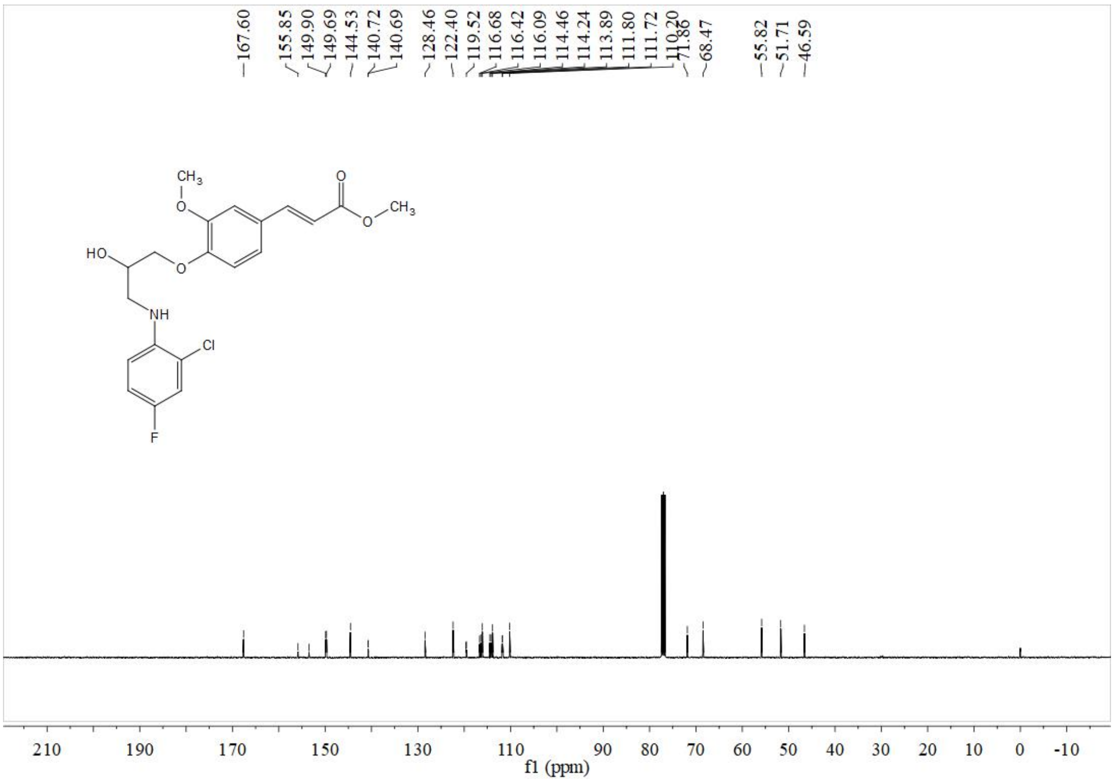

Figure S8 ^{13}C NMR Spectrum of **D3**

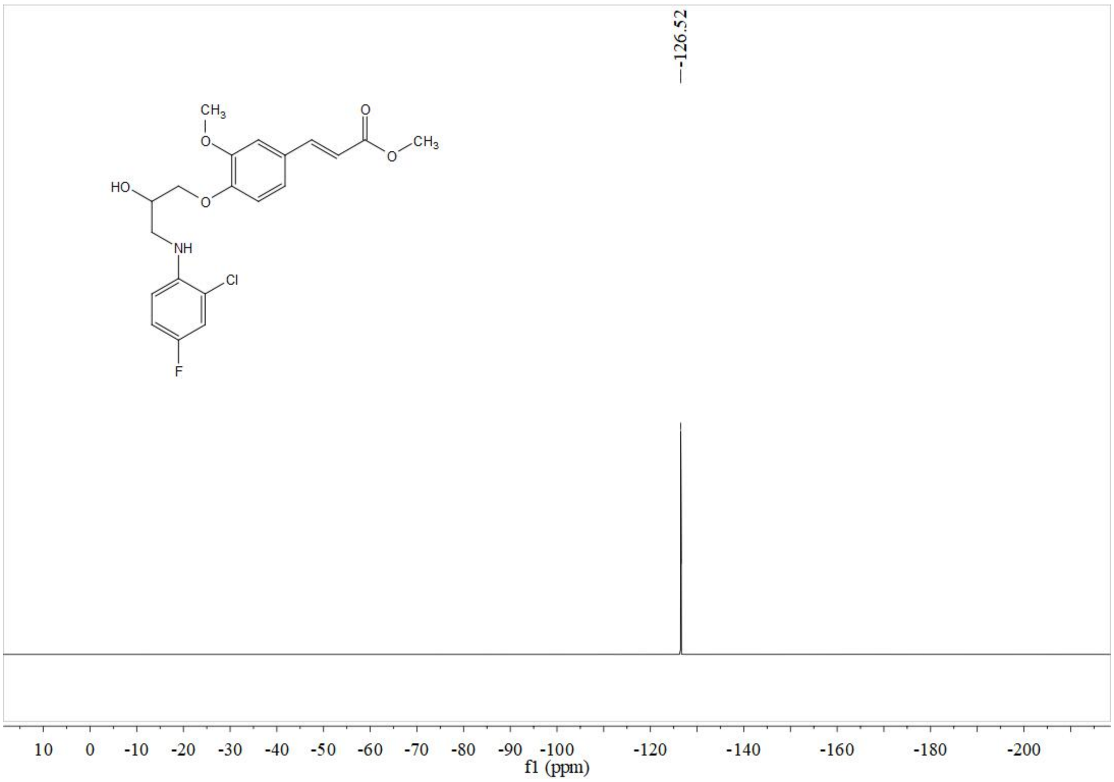

Figure S9 ^{19}F NMR Spectrum of **D3**

D4

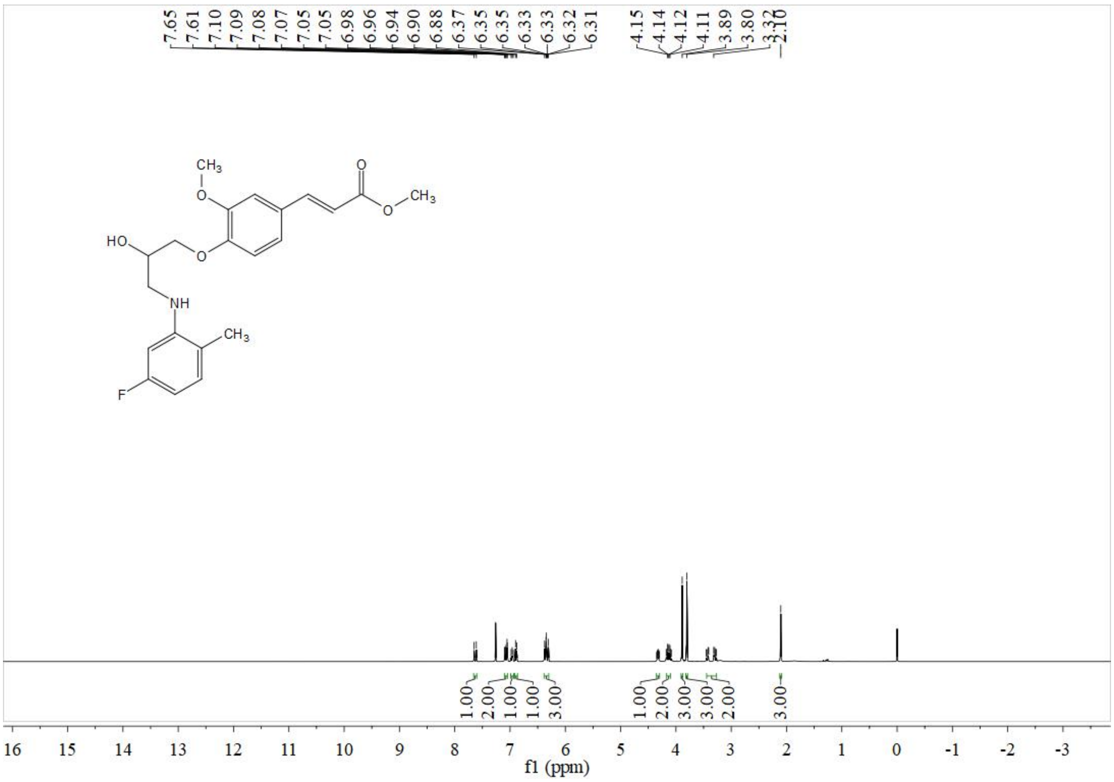

Figure S10 ^{1}H NMR Spectrum of **D4**

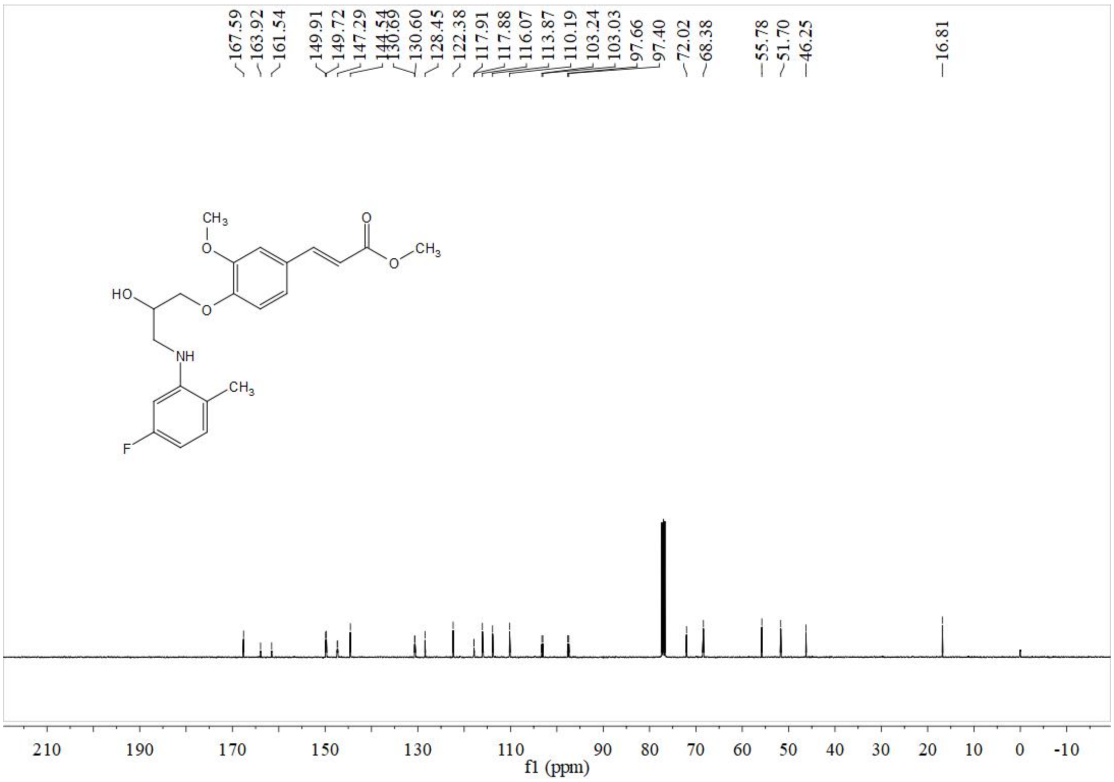

Figure S11 ^{13}C NMR Spectrum of **D4**

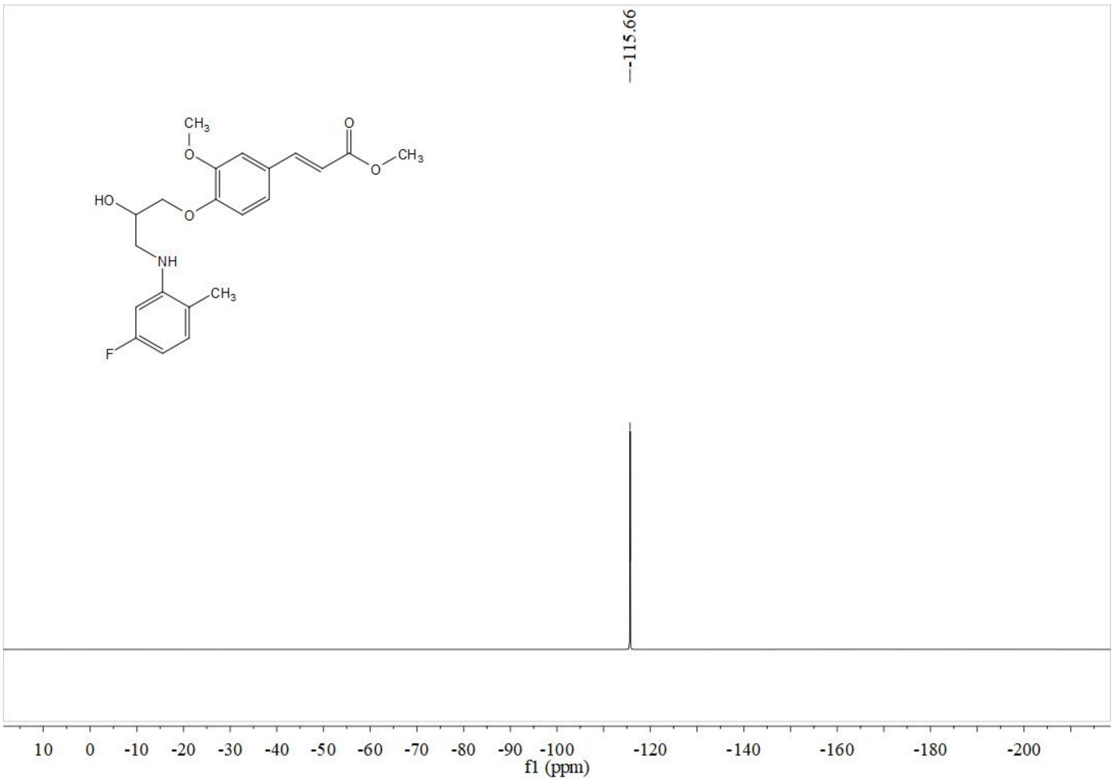

Figure S12 ^{19}F NMR Spectrum of **D4**

D5

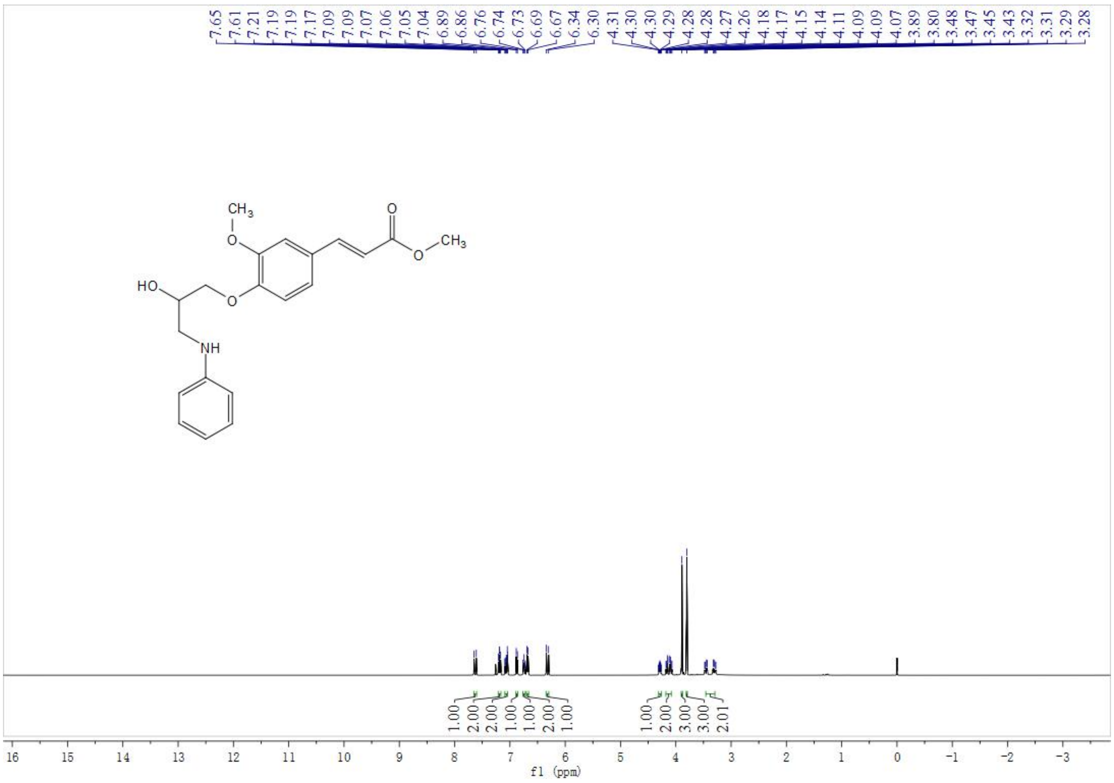

Figure S13 ^{1}H NMR Spectrum of **D5**

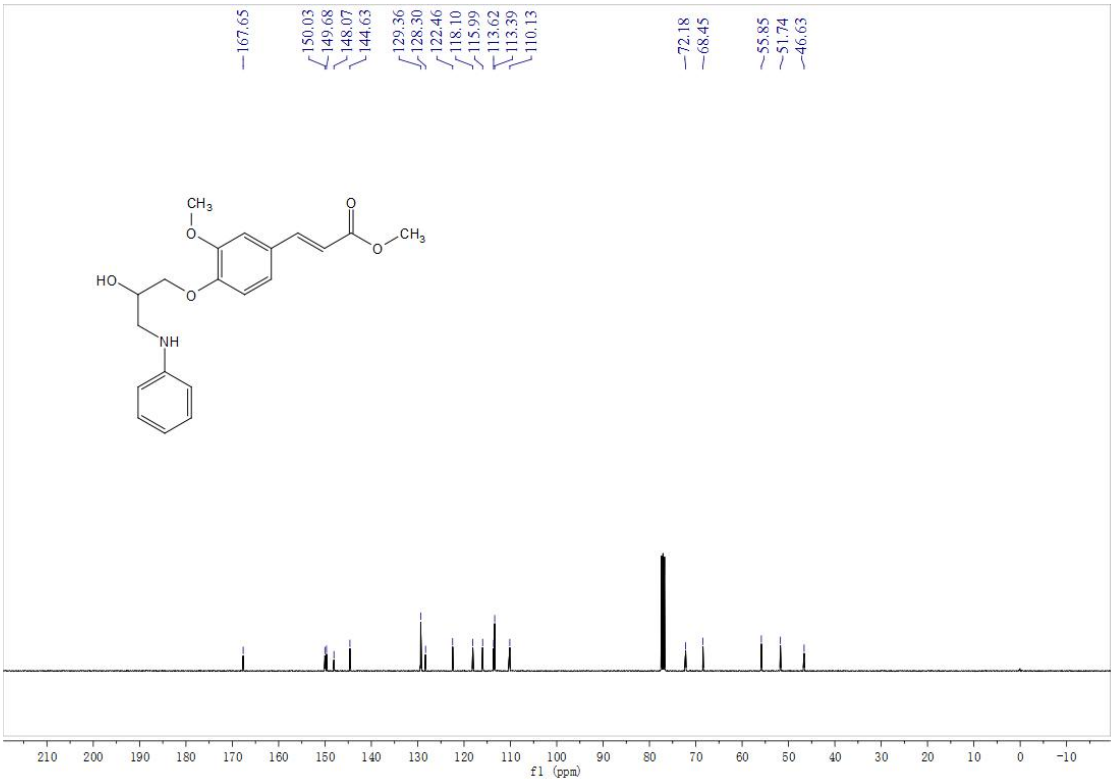

Figure S14 ^{13}C NMR Spectrum of **D5**

D6

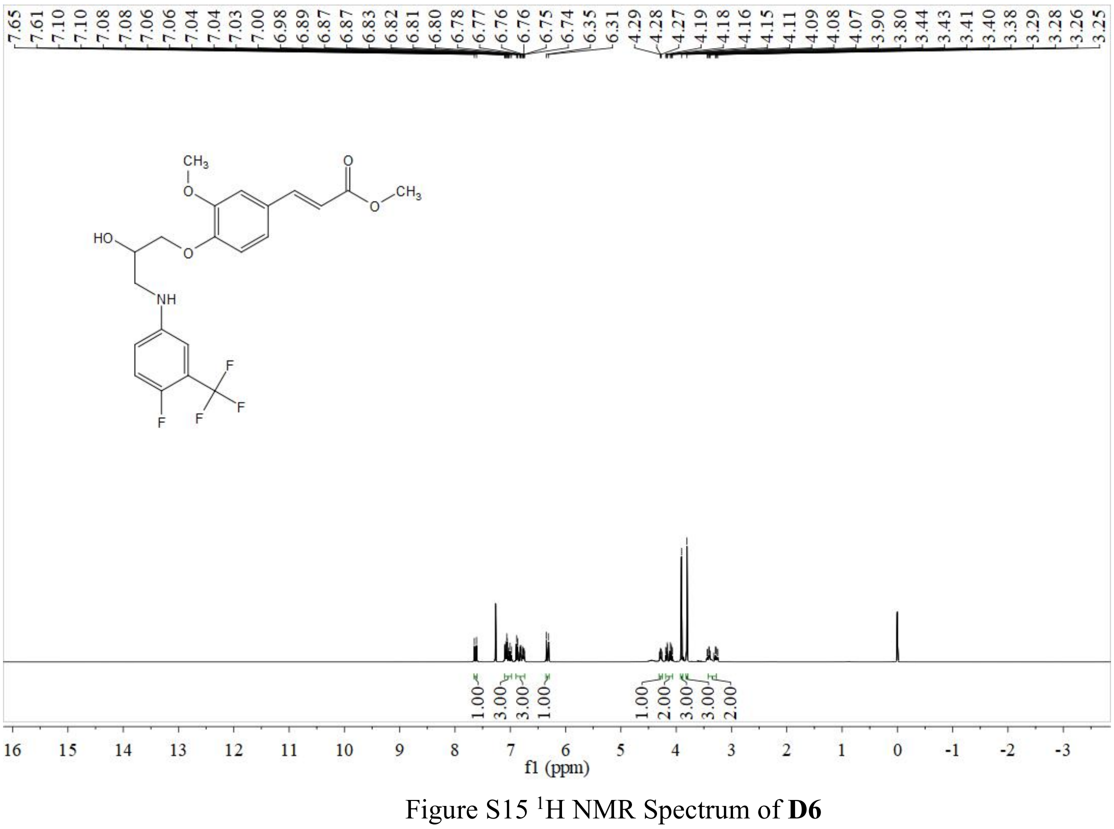

Figure S15 ^{1}H NMR Spectrum of **D6**

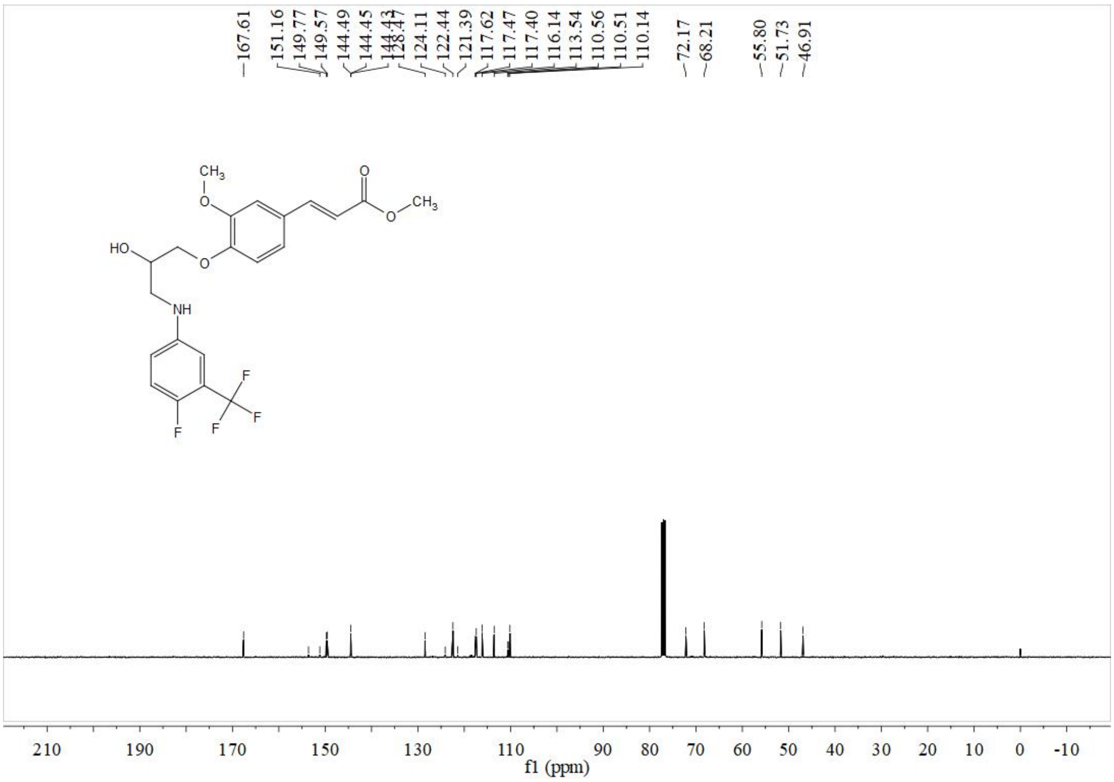

Figure S16 ^{13}C NMR Spectrum of **D6**

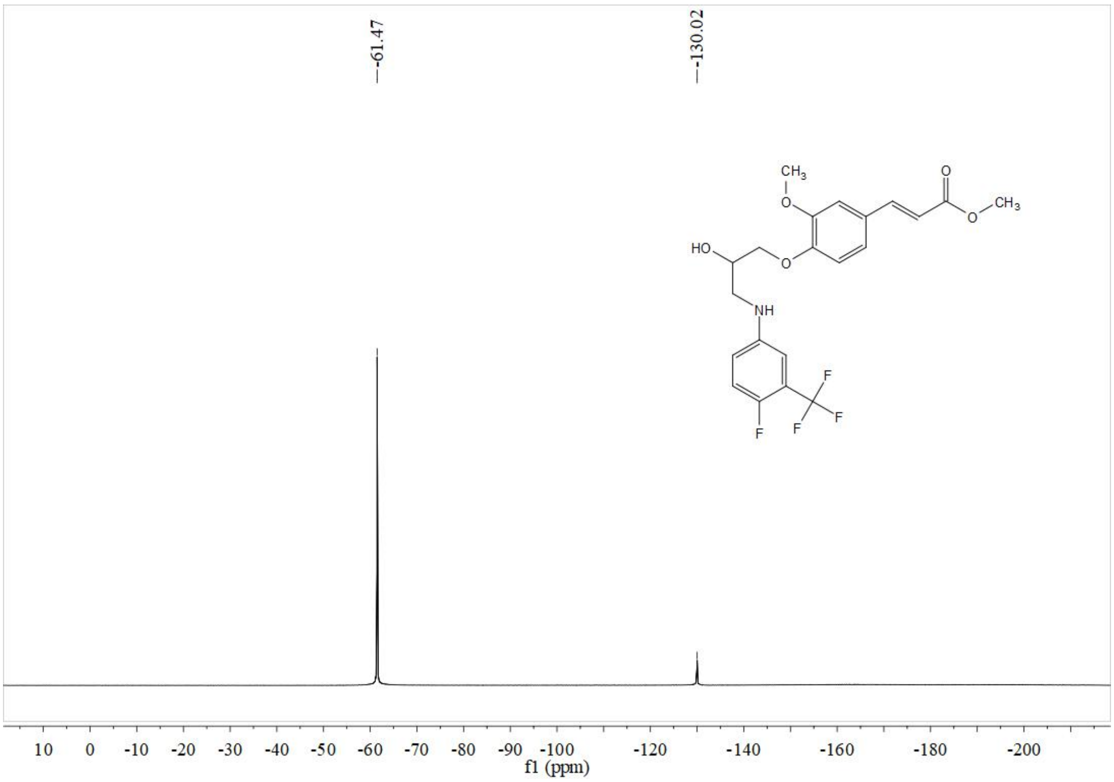

Figure S17 ^{19}F NMR Spectrum of **D6**

D7

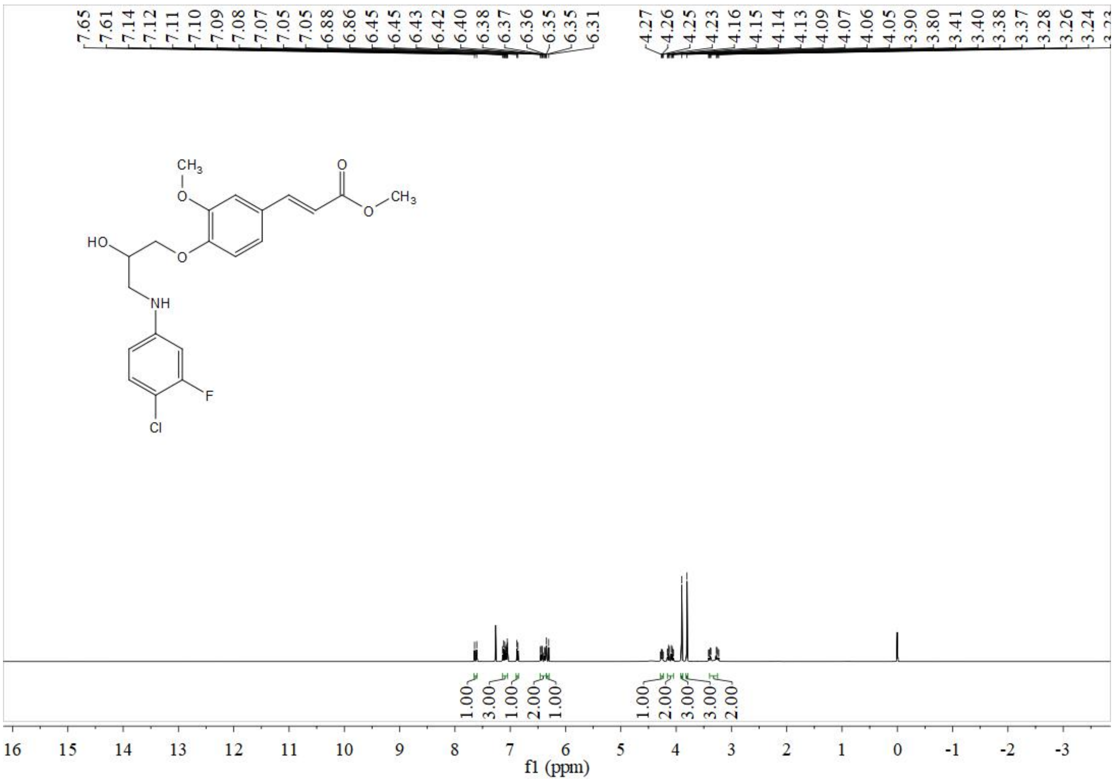

Figure S18 ^{1}H NMR Spectrum of **D7**

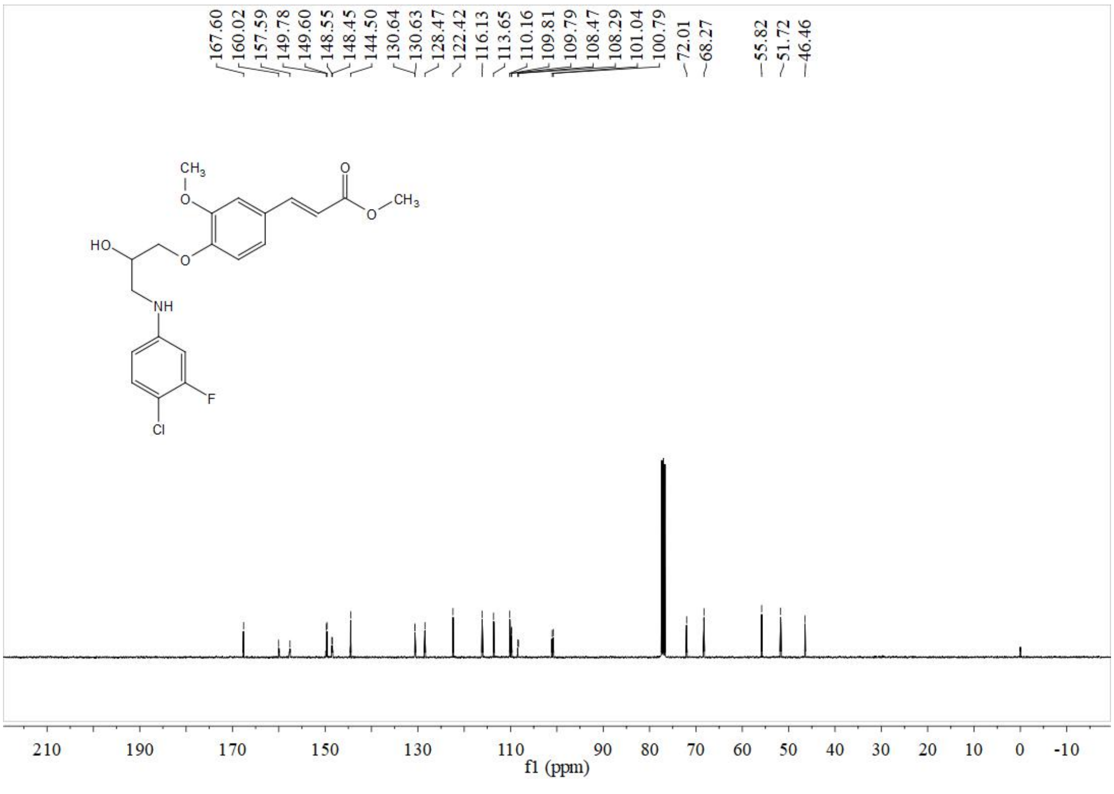

Figure S19 ^{13}C NMR Spectrum of **D7**

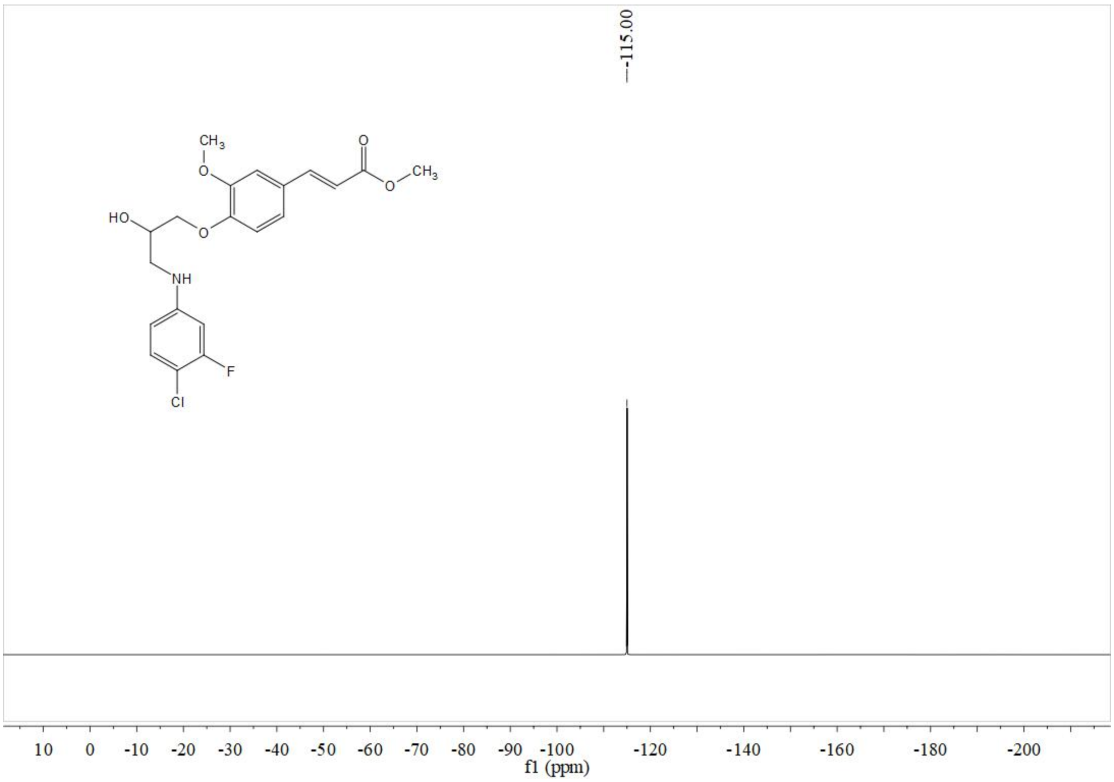

Figure S20 ^{19}F NMR Spectrum of **D7**

D8

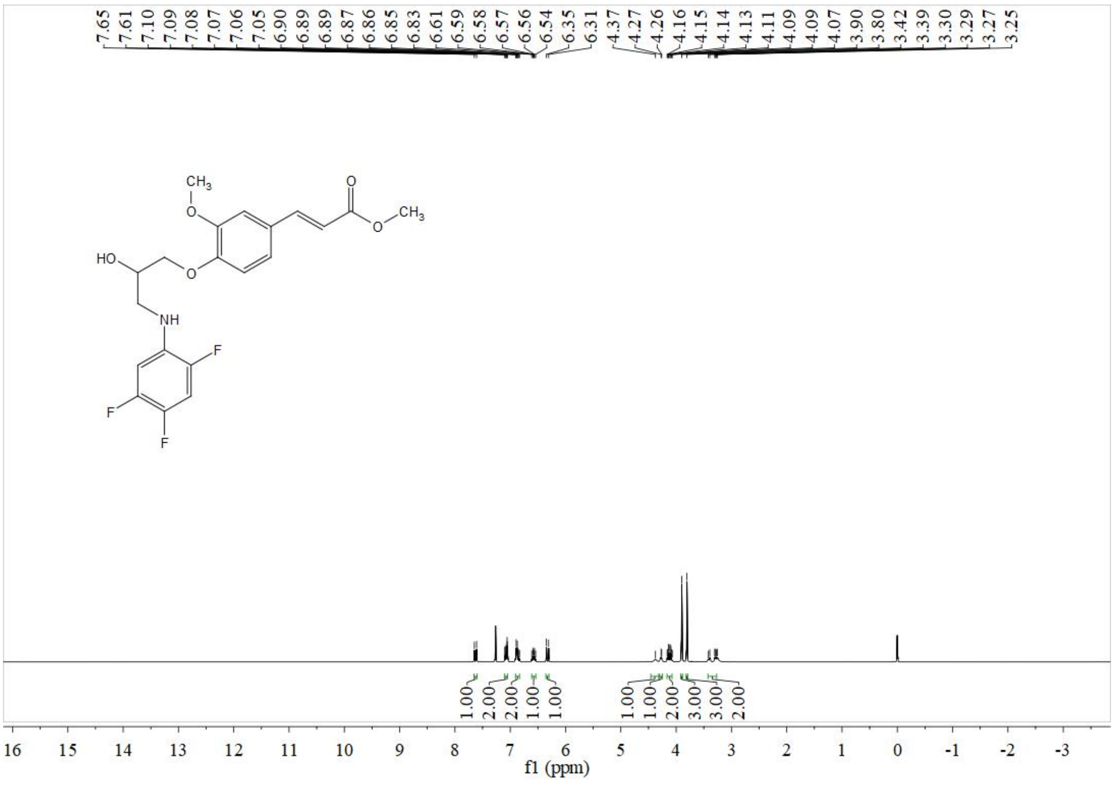

Figure S21 ^{1}H NMR Spectrum of **D8**

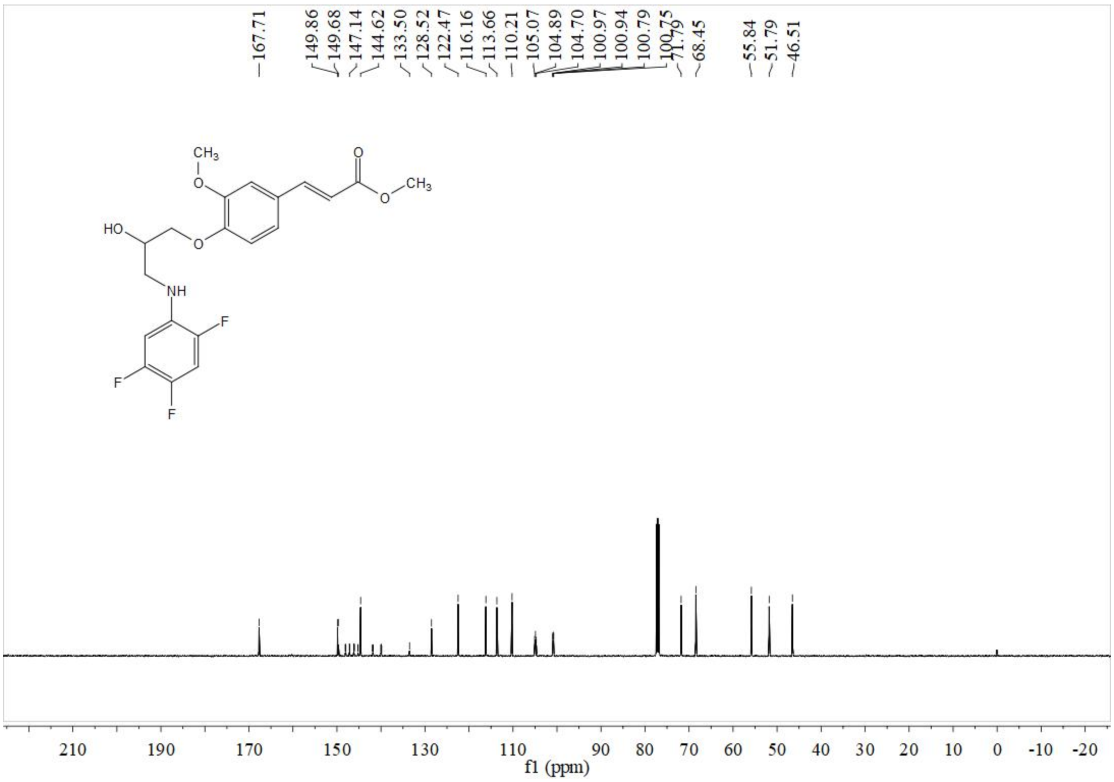

Figure S22 ^{13}C NMR Spectrum of **D8**

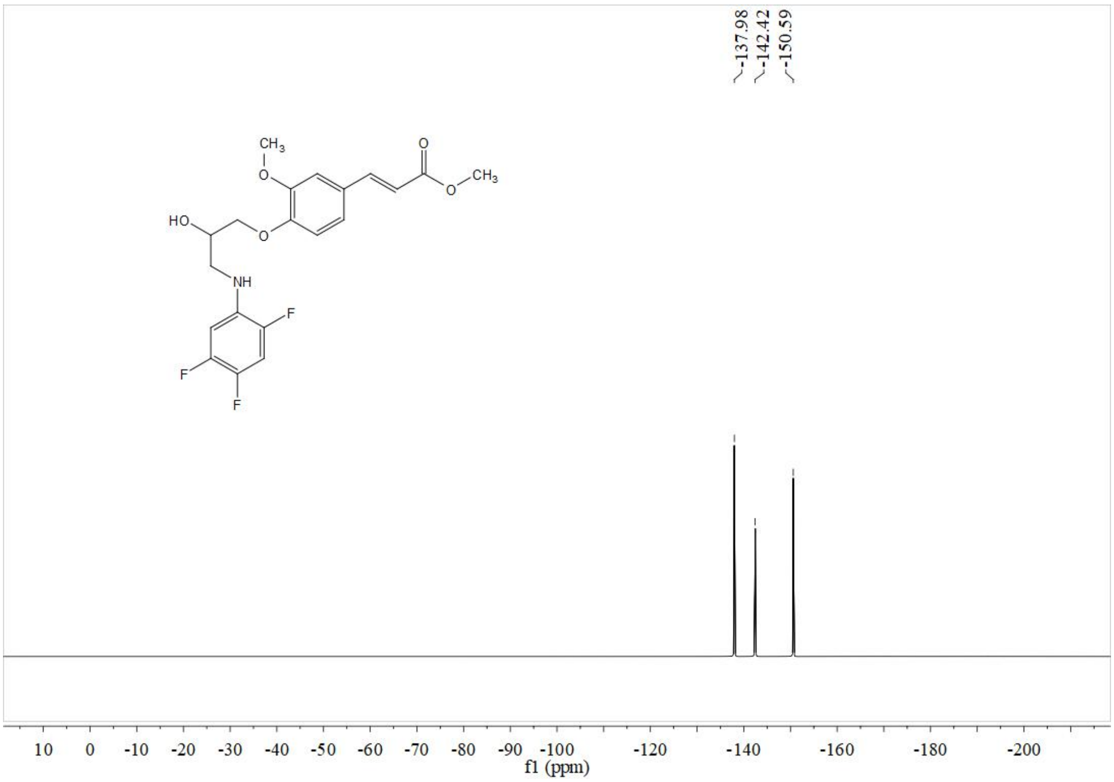

Figure S23 ^{19}F NMR Spectrum of **D8**

D9

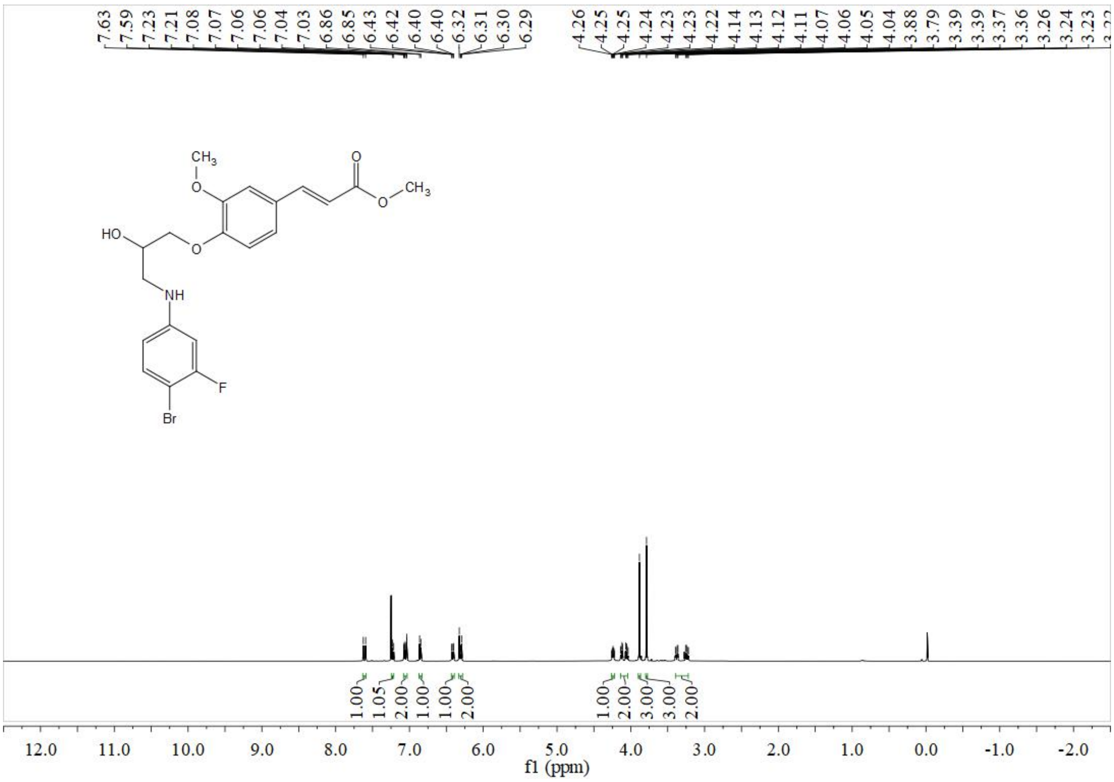

Figure S24 ^{1}H NMR Spectrum of **D9**

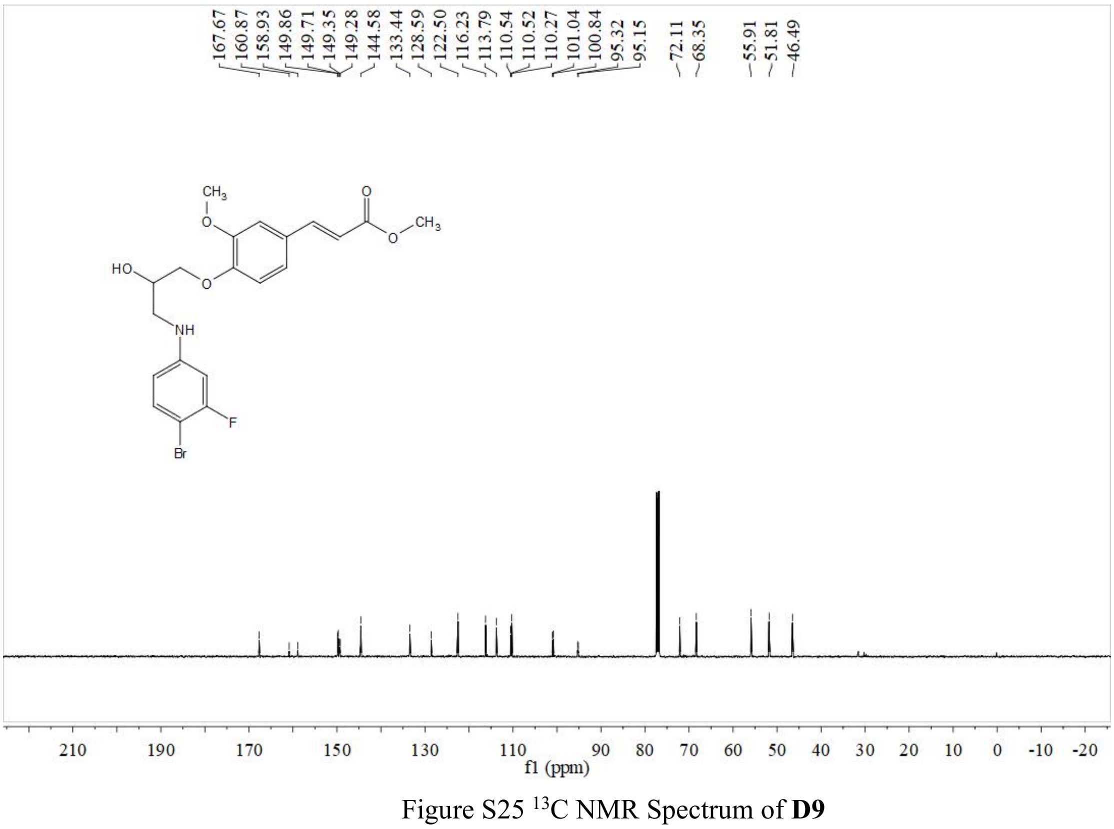

Figure S25 ^{13}C NMR Spectrum of **D9**

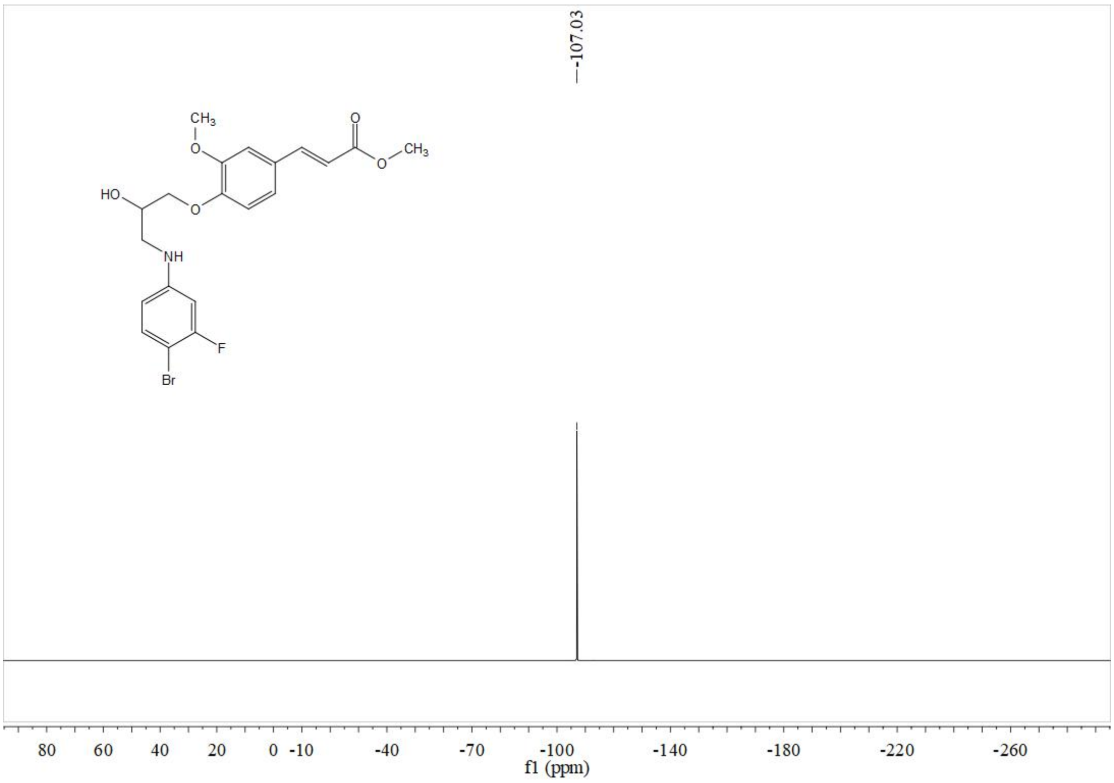

Figure S26 ^{19}F NMR Spectrum of **D9**

D10

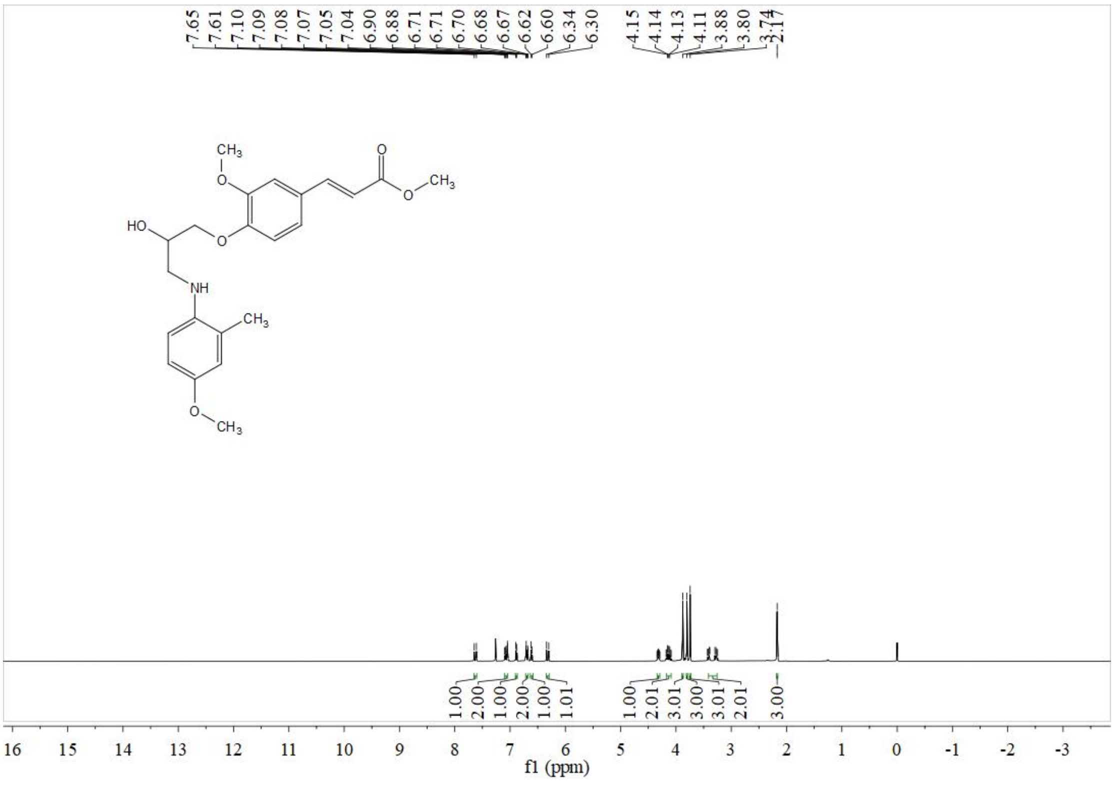

Figure S27 ^{1}H NMR Spectrum of **D10**

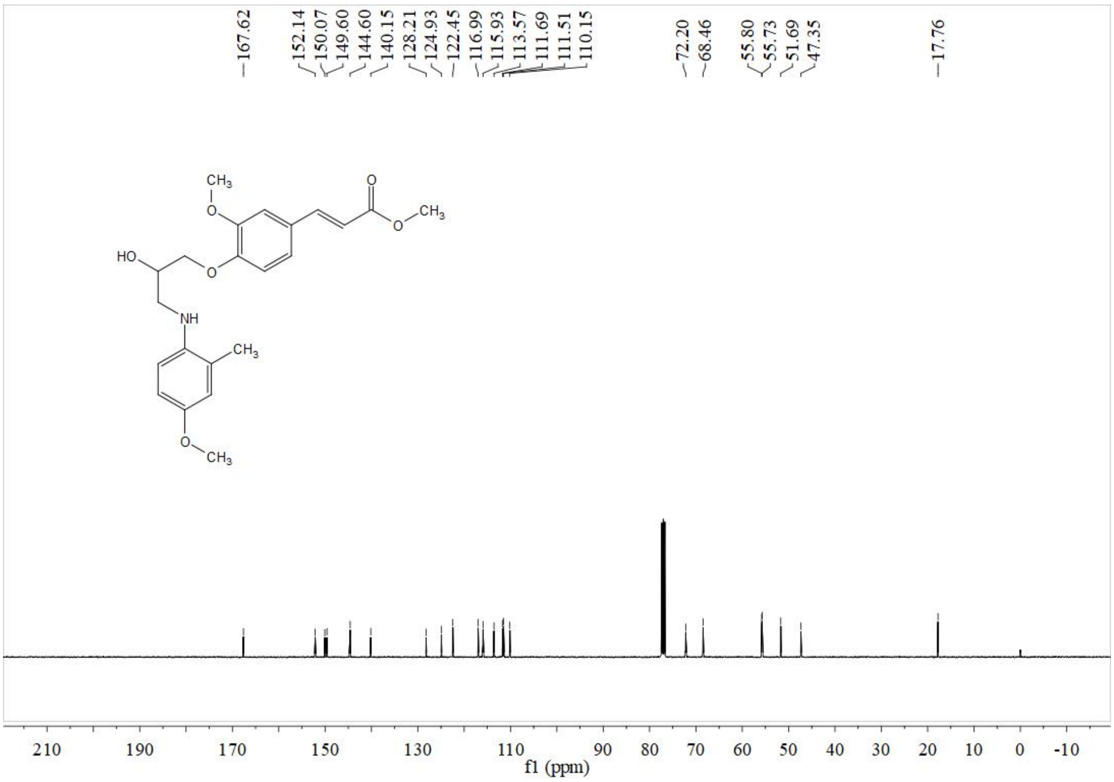

Figure S28 ^{13}C NMR Spectrum of **D10**

D11

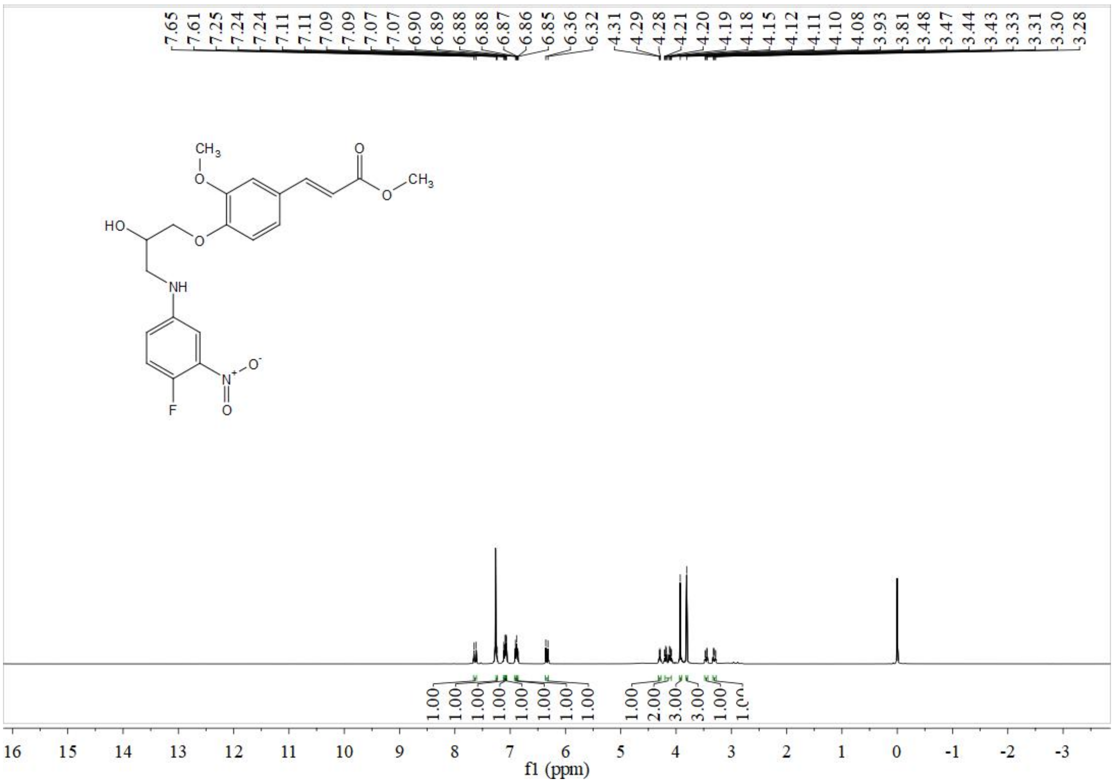

Figure S29 ^{1}H NMR Spectrum of **D11**

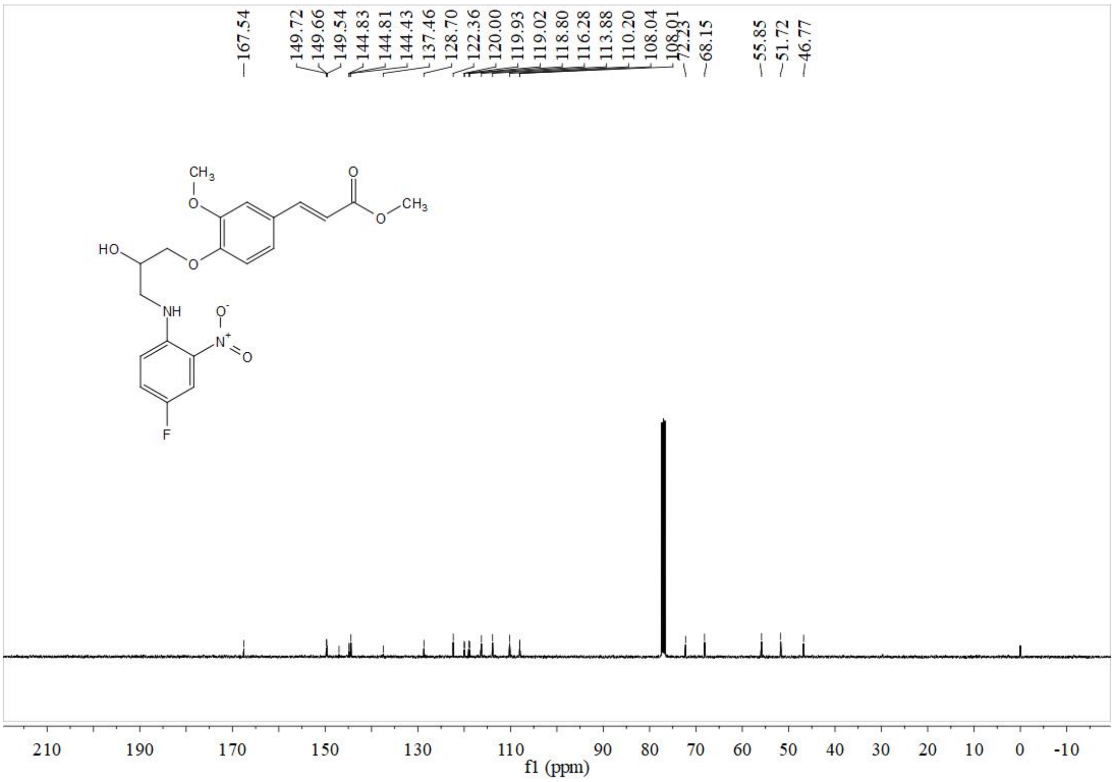

Figure S30 ^{13}C NMR Spectrum of **D11**

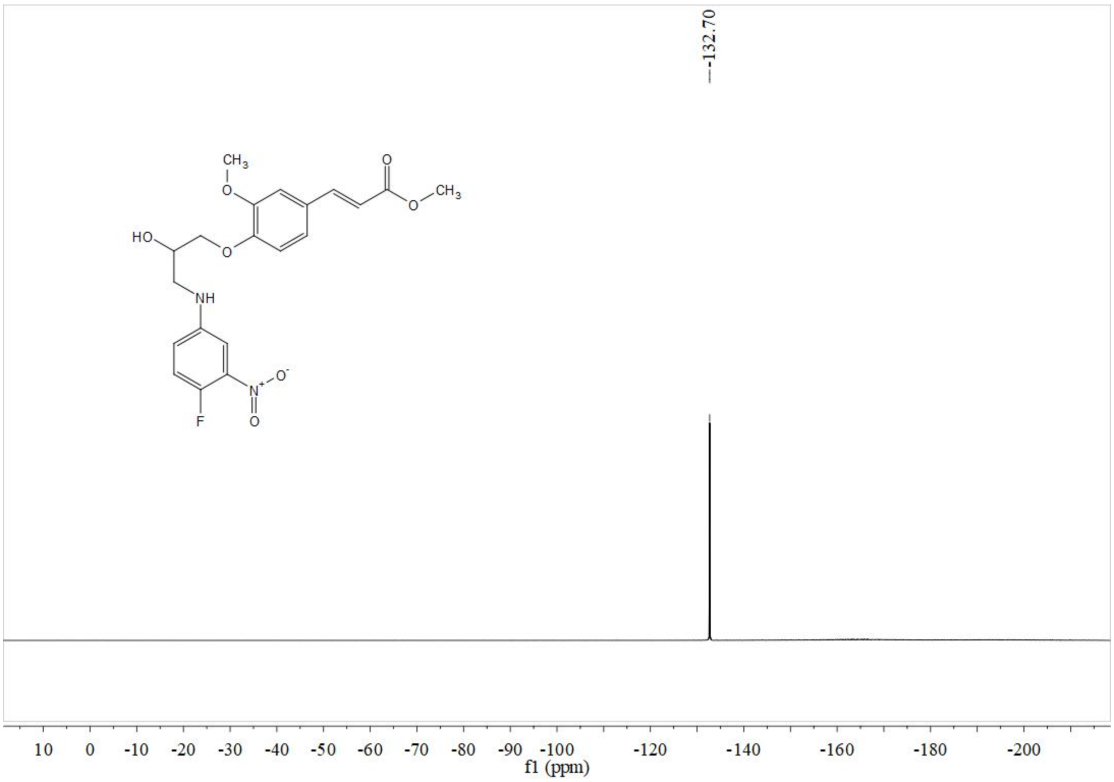

Figure S31 ^{1}H NMR Spectrum of **D12**

D12

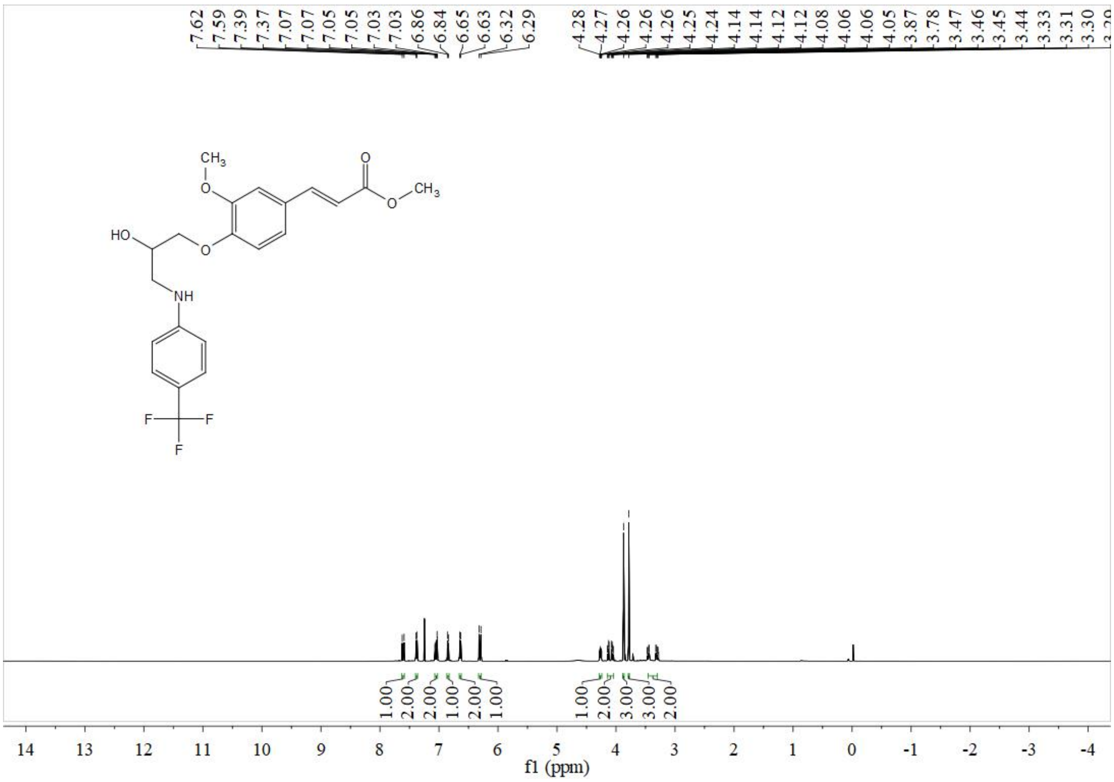

Figure S32 ^{1}H NMR Spectrum of **D12**

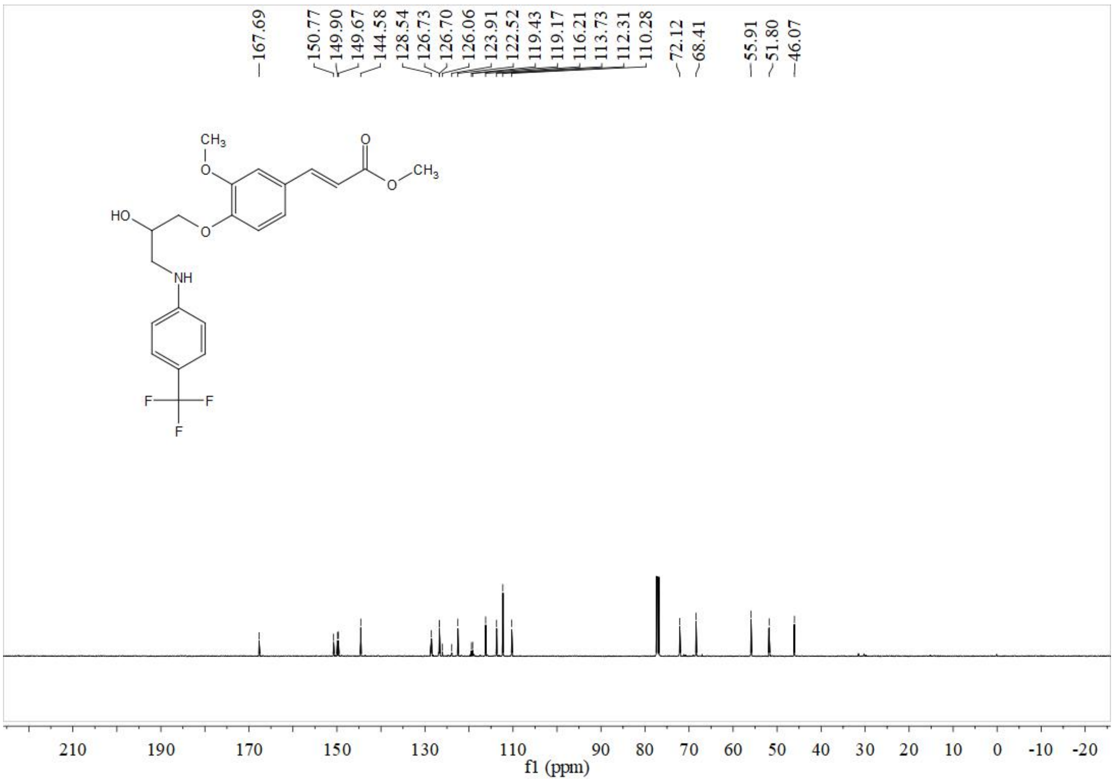

Figure S33 ^{13}C NMR Spectrum of **D12**

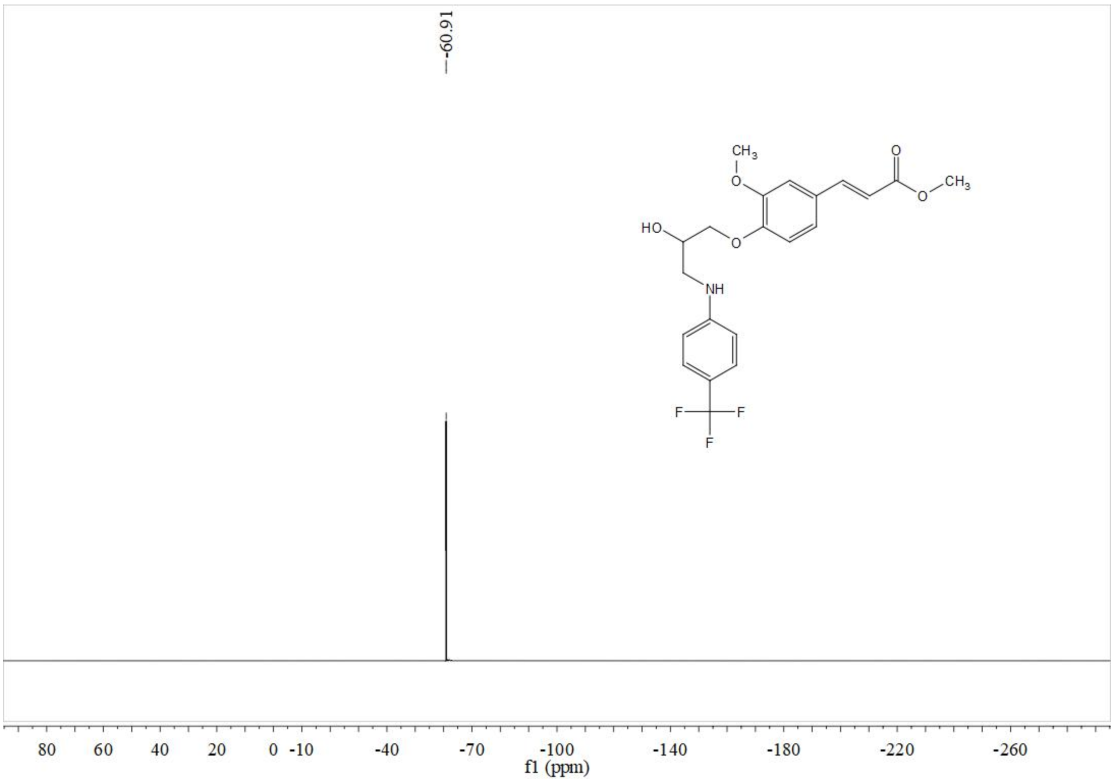

Figure S34 ^{19}F NMR Spectrum of **D12**

D13

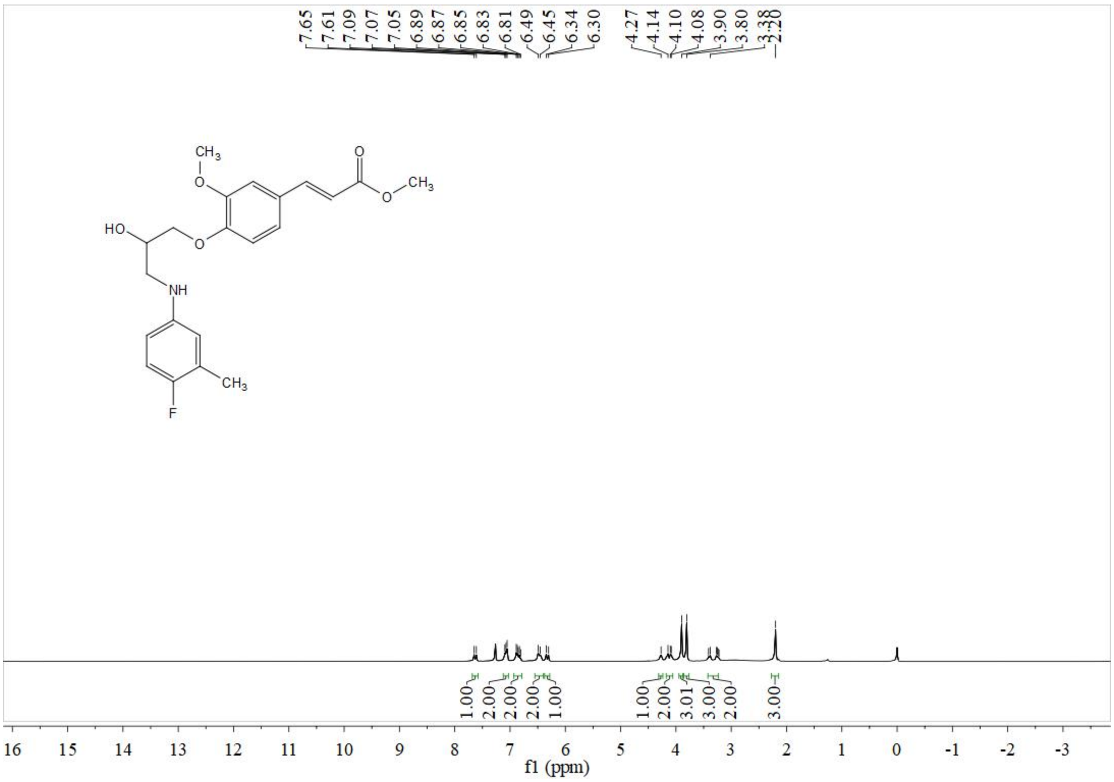

Figure S35 ^{1}H NMR Spectrum of **D13**

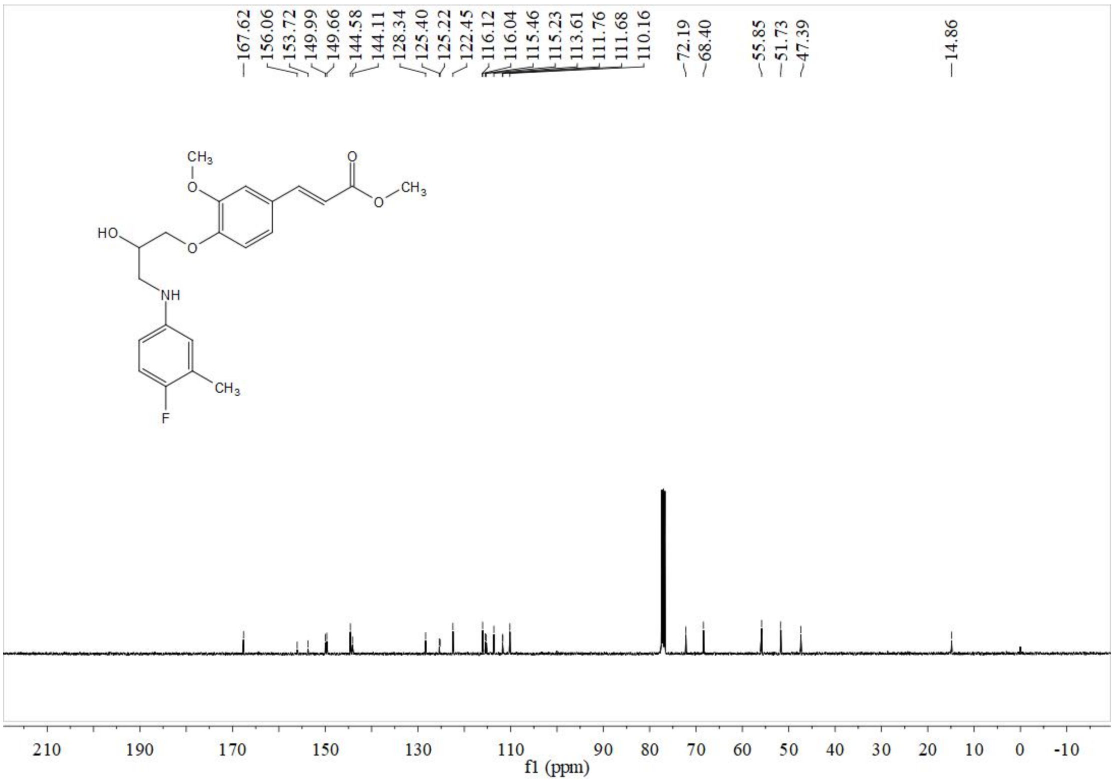

Figure S36 ^{13}C NMR Spectrum of **D13**

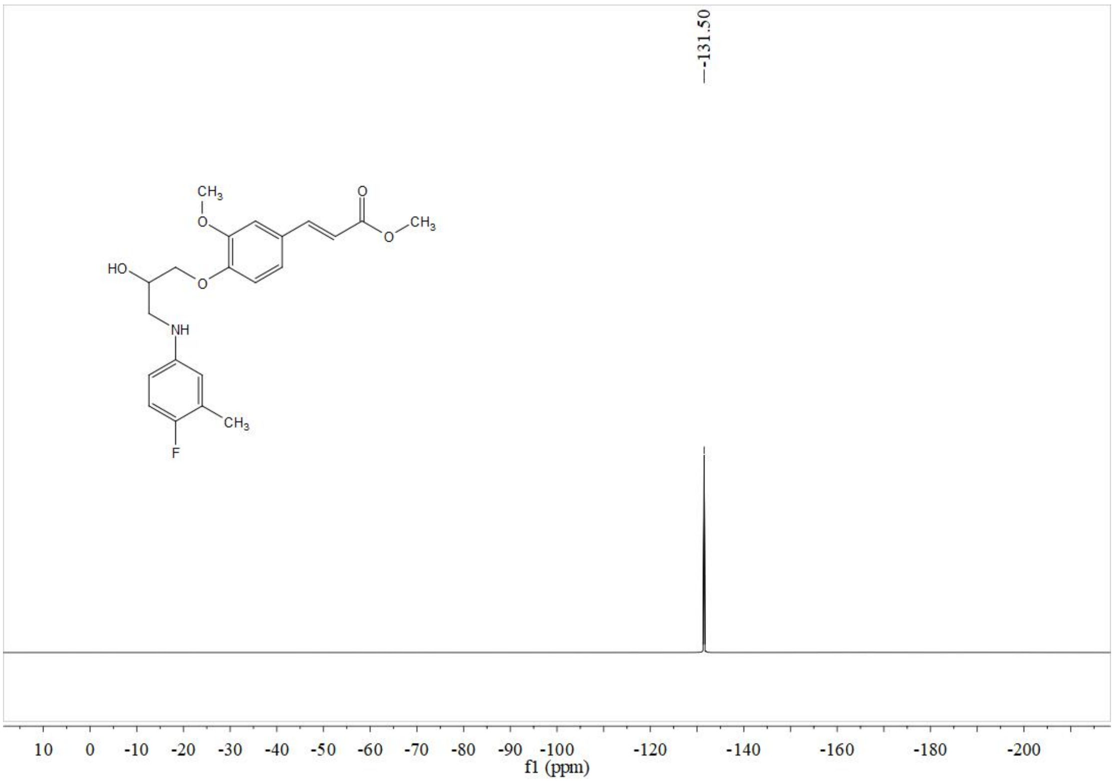

Figure S37 ^{19}F NMR Spectrum of **D13**

D14

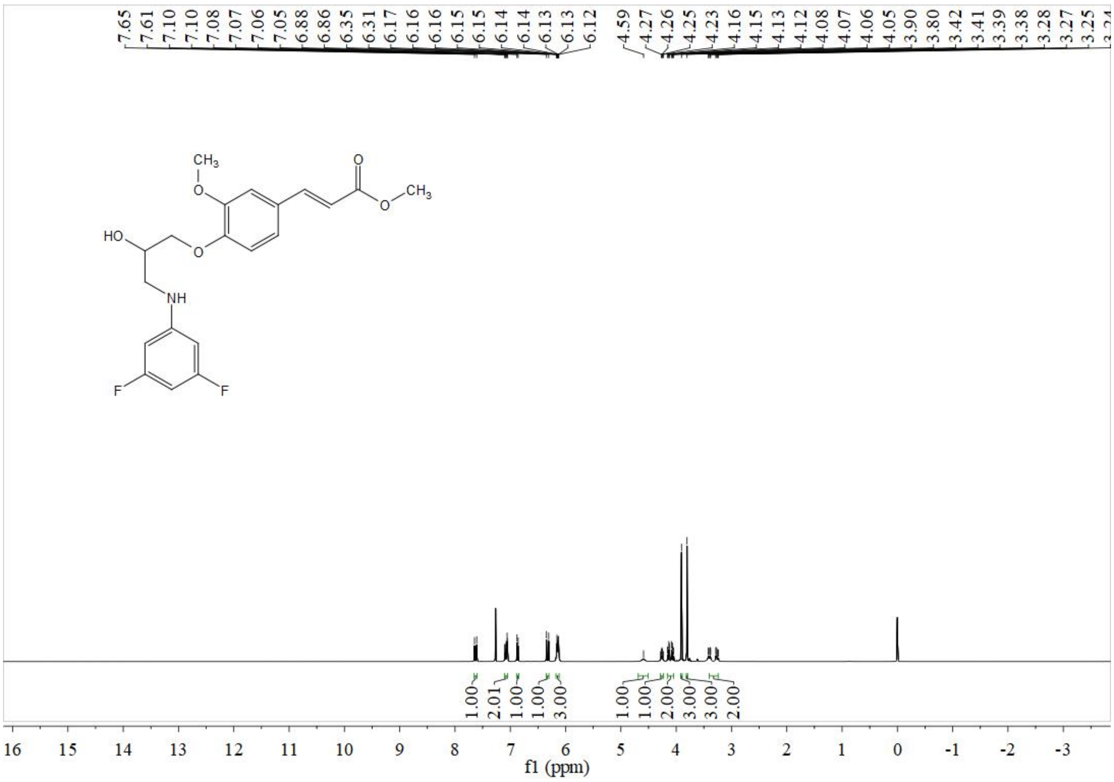

Figure S38 ^{1}H NMR Spectrum of **D14**

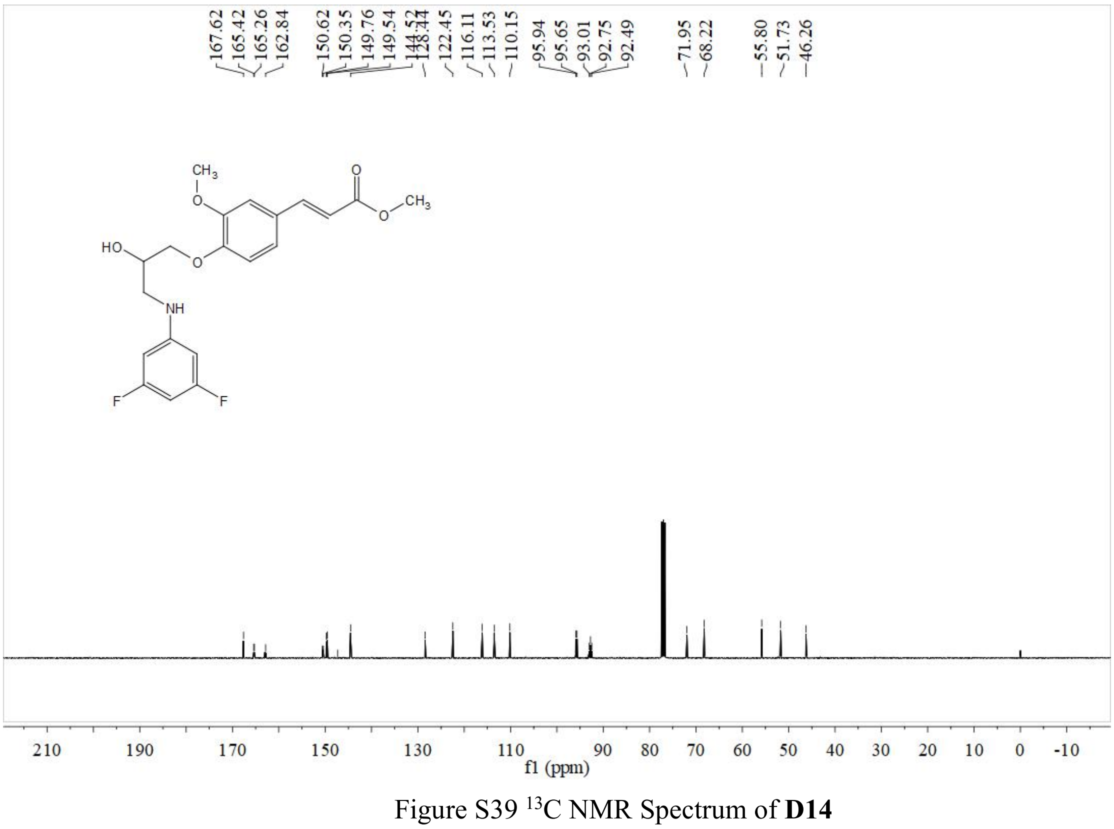

Figure S39 ^{13}C NMR Spectrum of **D14**

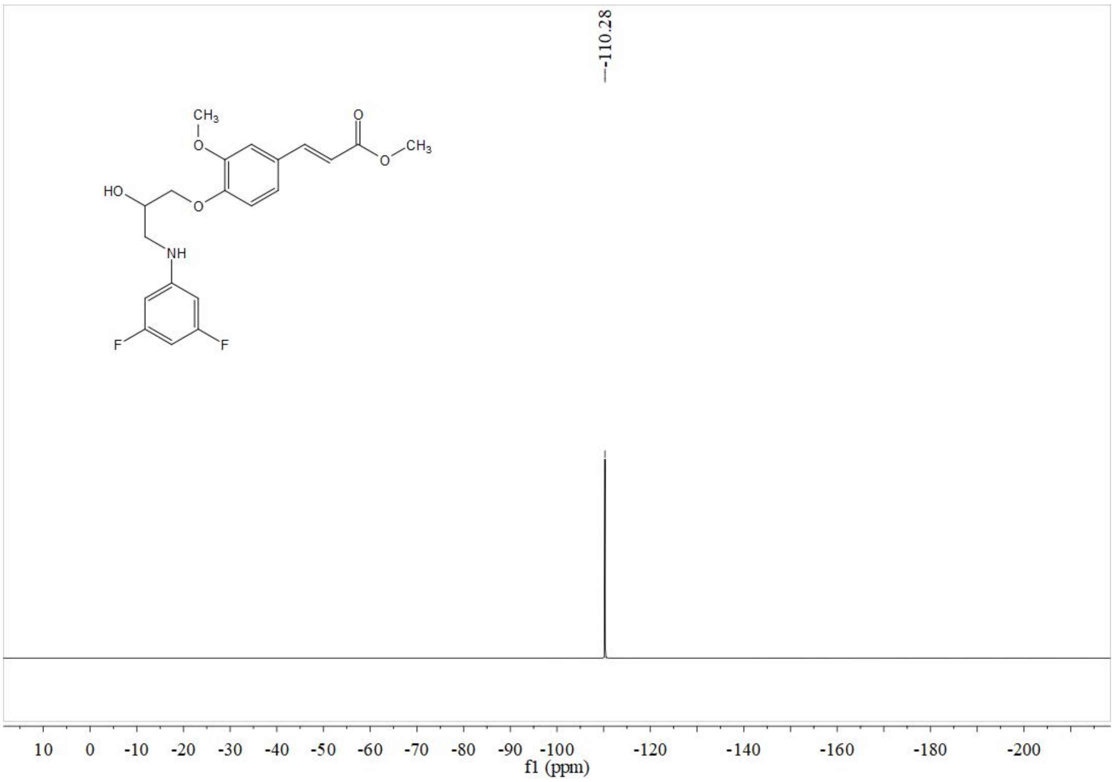

Figure S40 ^{19}F NMR Spectrum of **D14**

D15

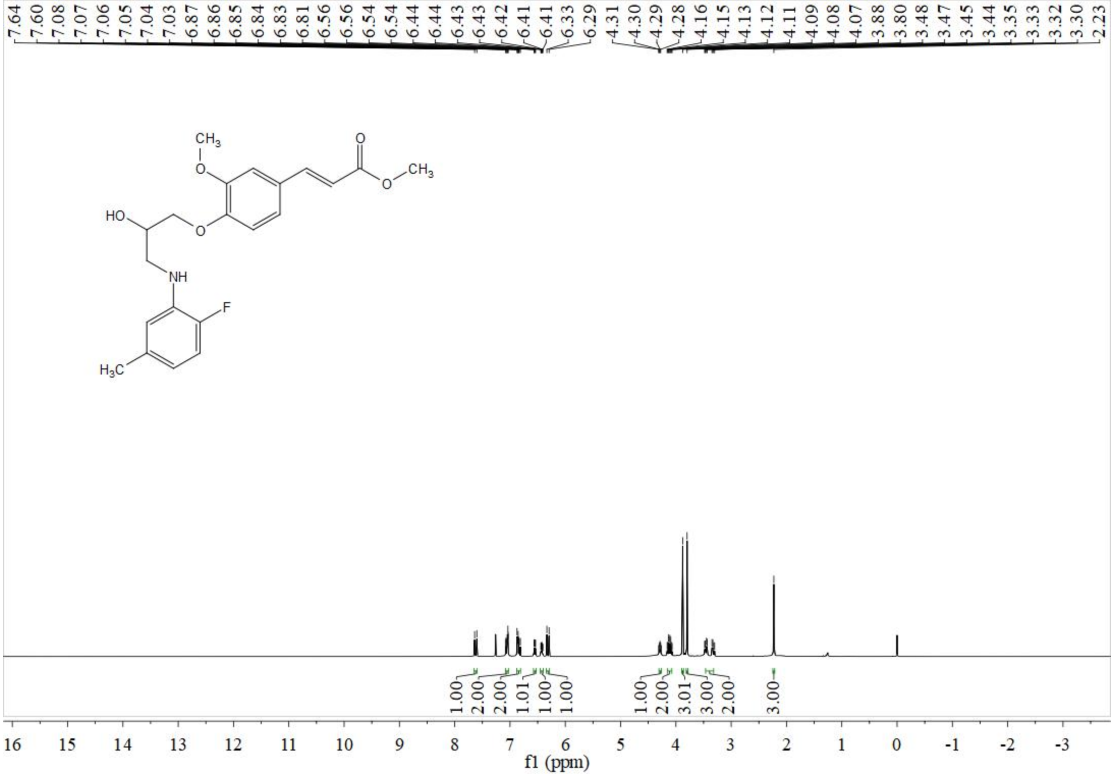

Figure S41 ^{1}H NMR Spectrum of **D15**

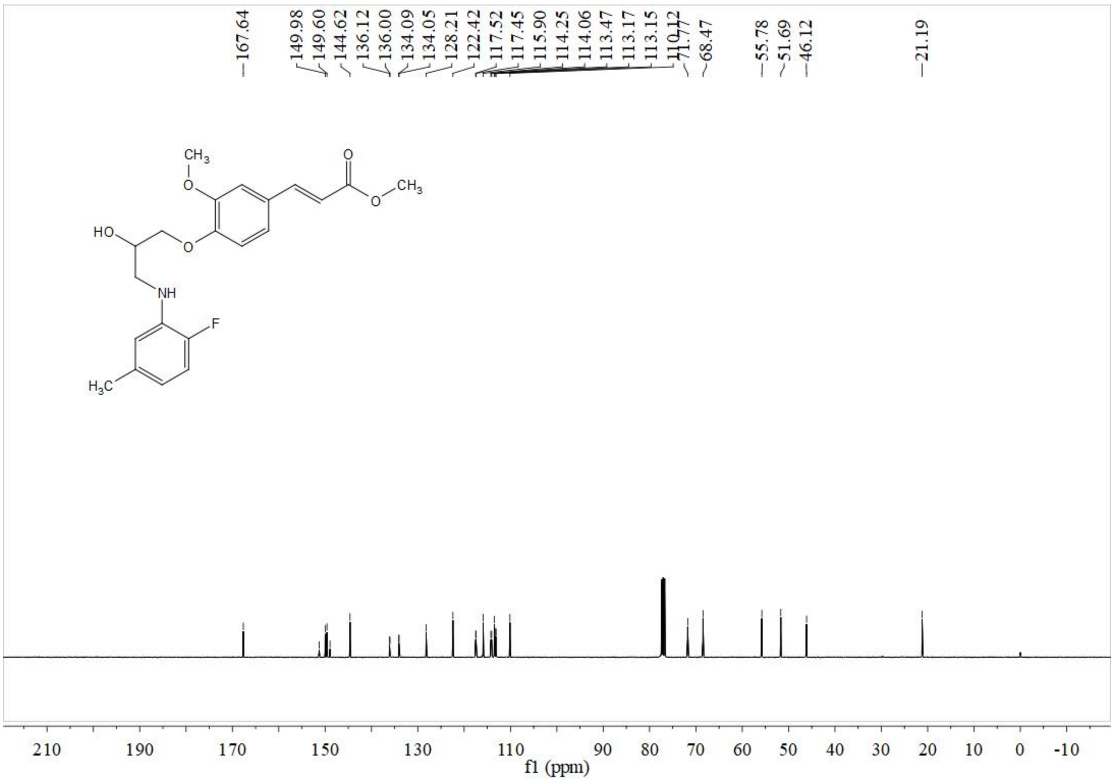

Figure S42 ^{13}C NMR Spectrum of **D15**

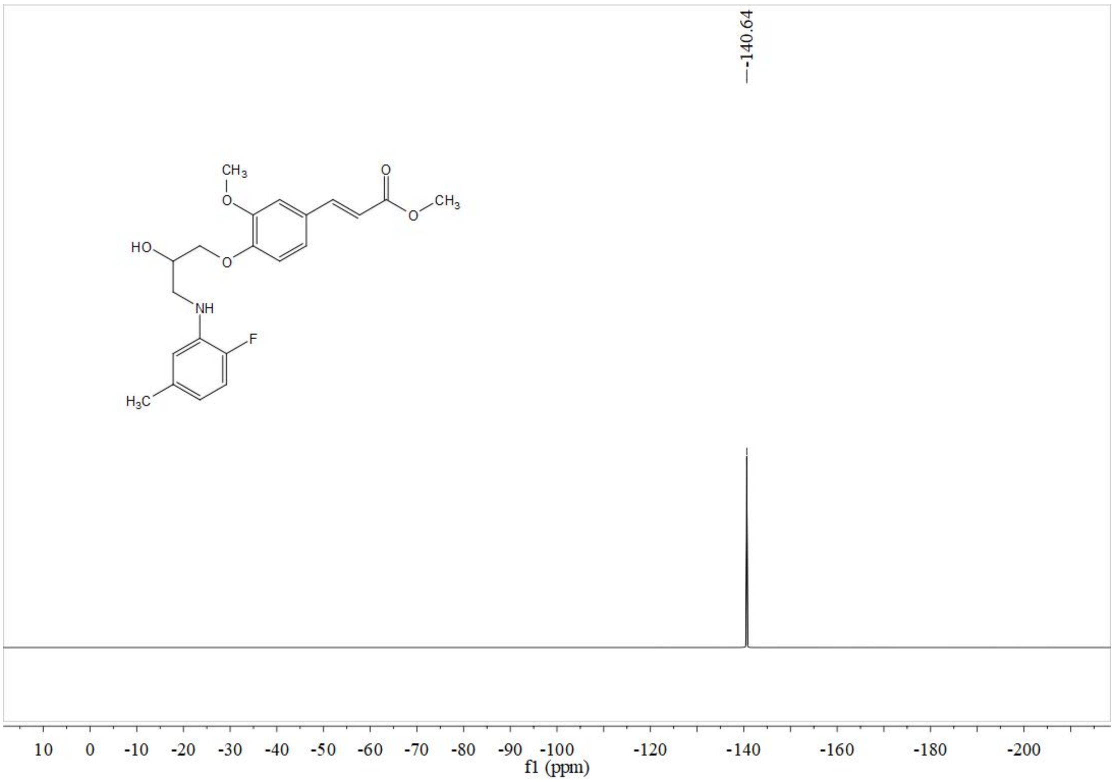

Figure S43 ^{19}F NMR Spectrum of **D15**

D16

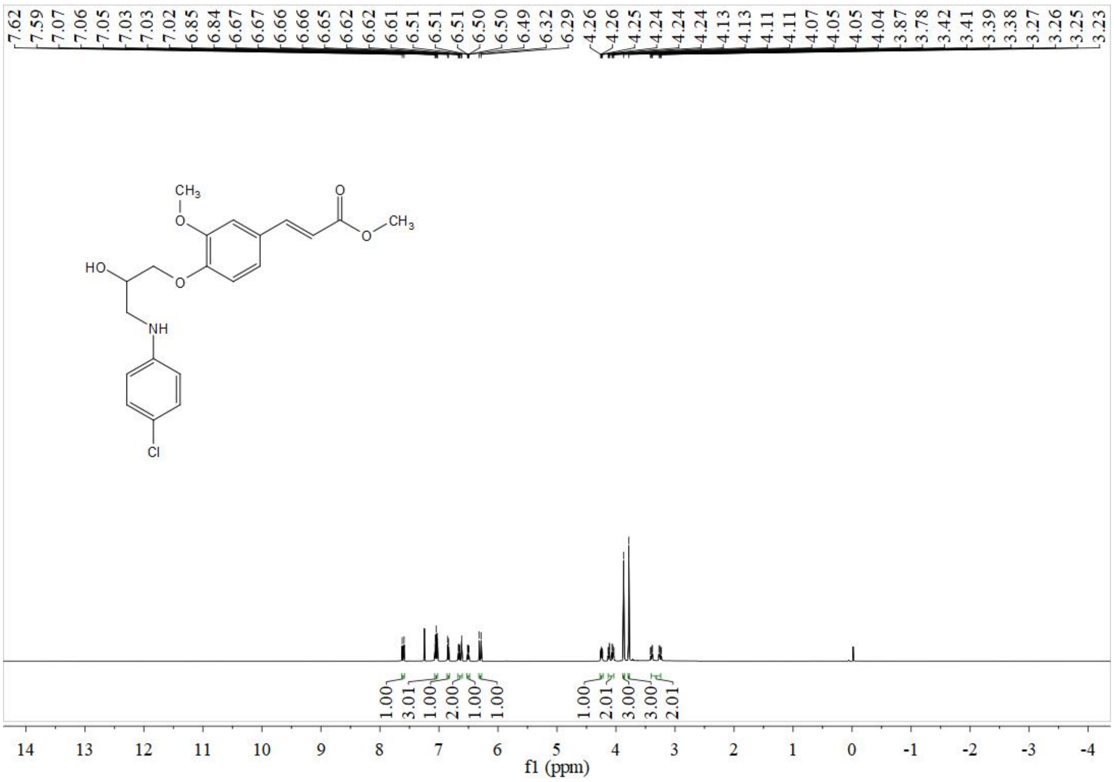

Figure S44 ^{1}H NMR Spectrum of **D16**

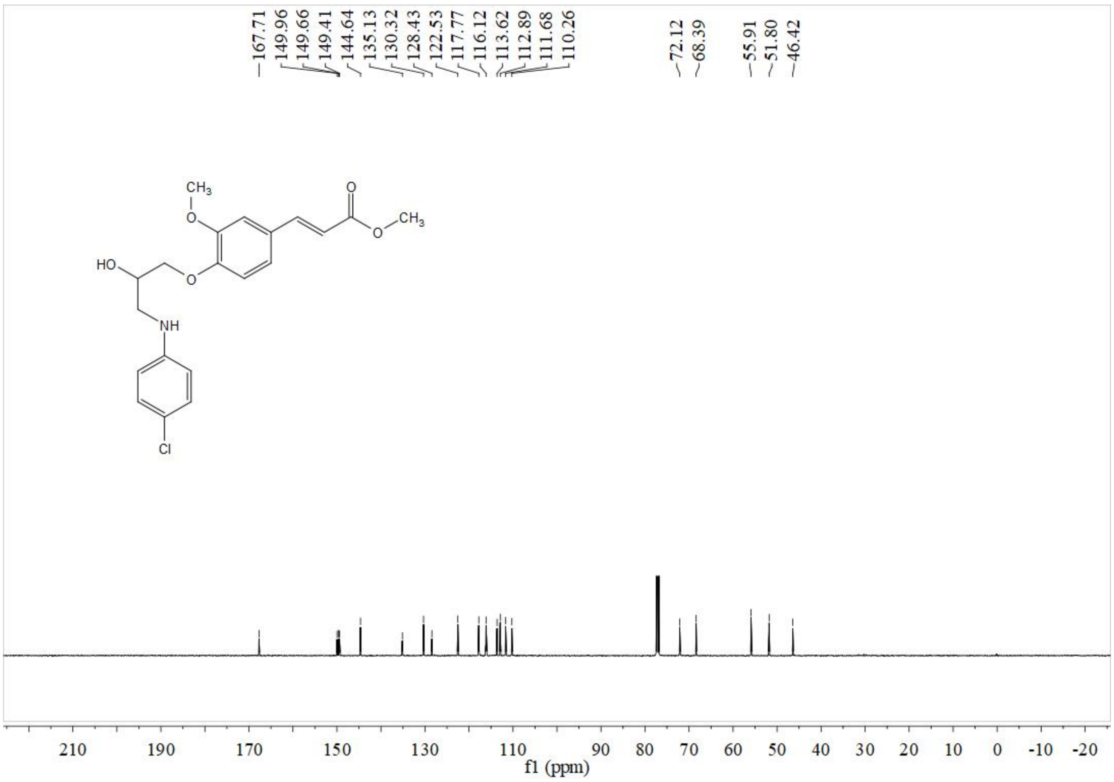

Figure S45 ^{13}C NMR Spectrum of **D16**

D17

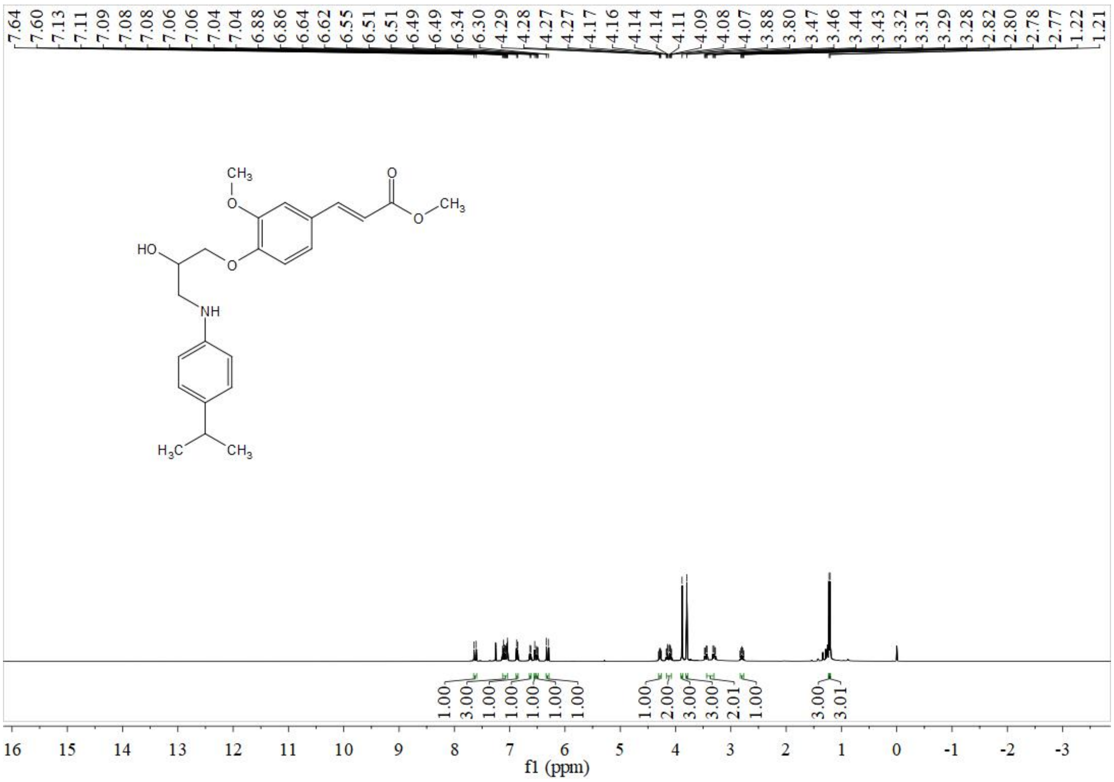

Figure S46 ^{1}H NMR Spectrum of **D17**

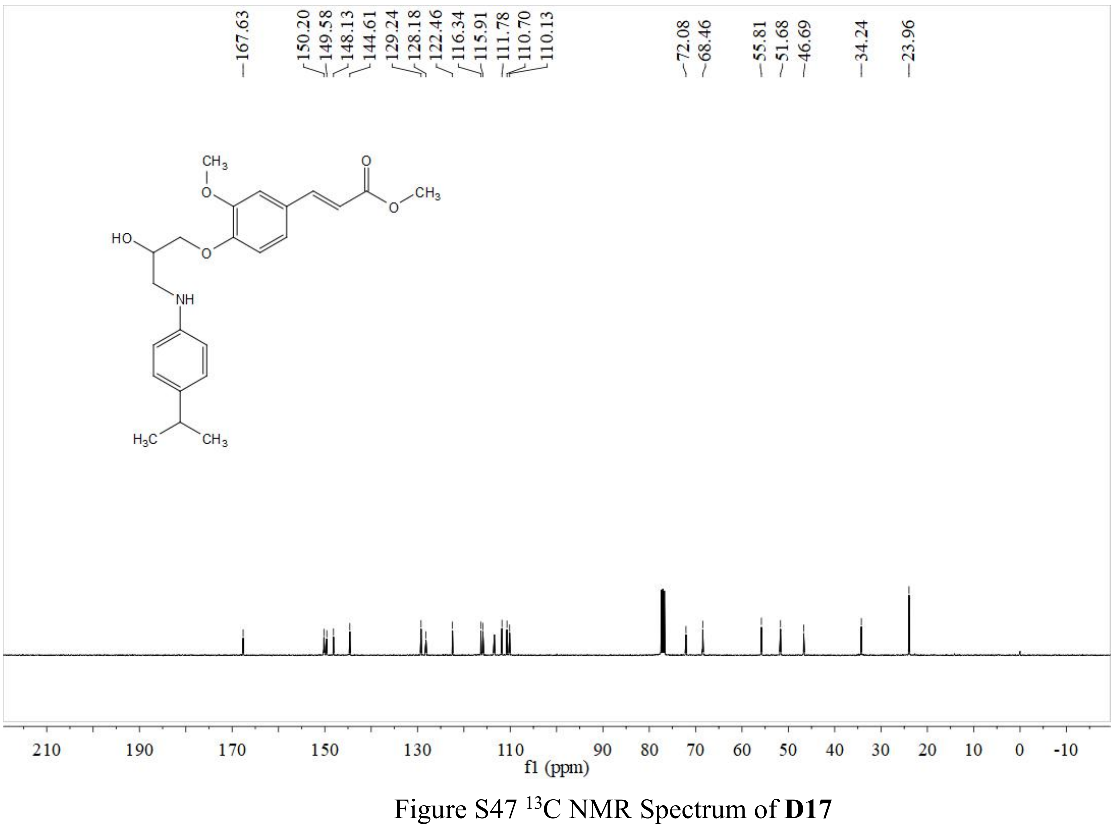

Figure S47 ^{13}C NMR Spectrum of **D17**

D18

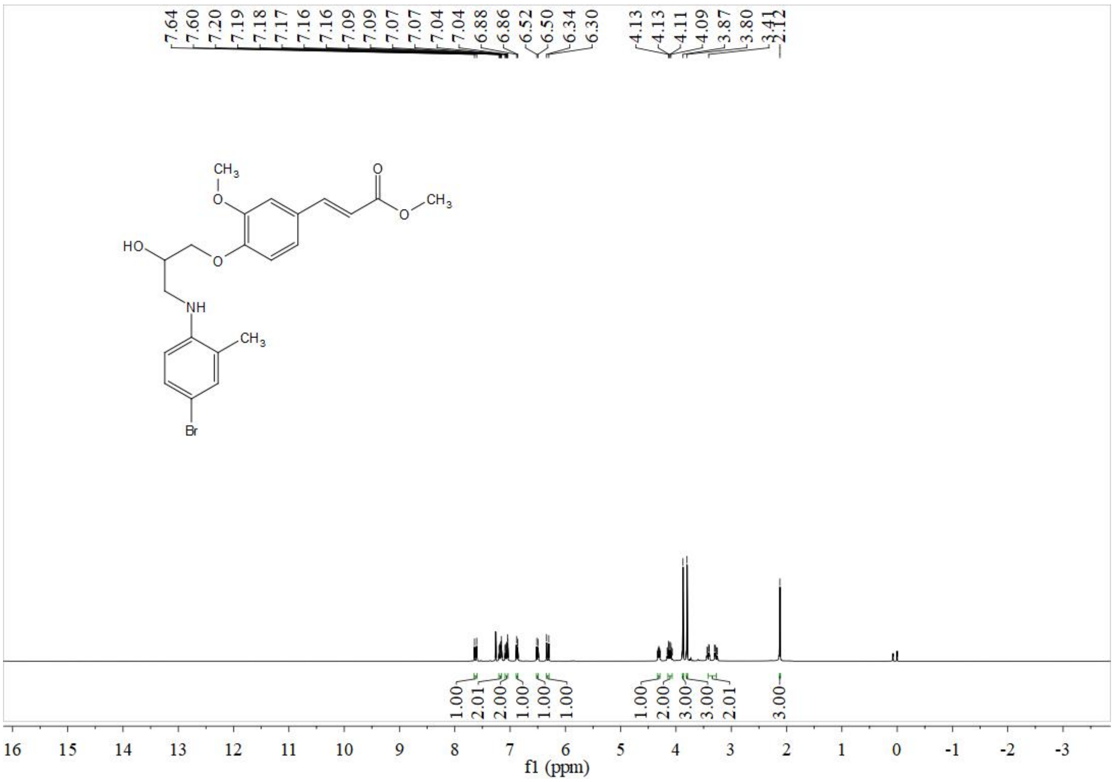

Figure S48 ^{1}H NMR Spectrum of **D18**

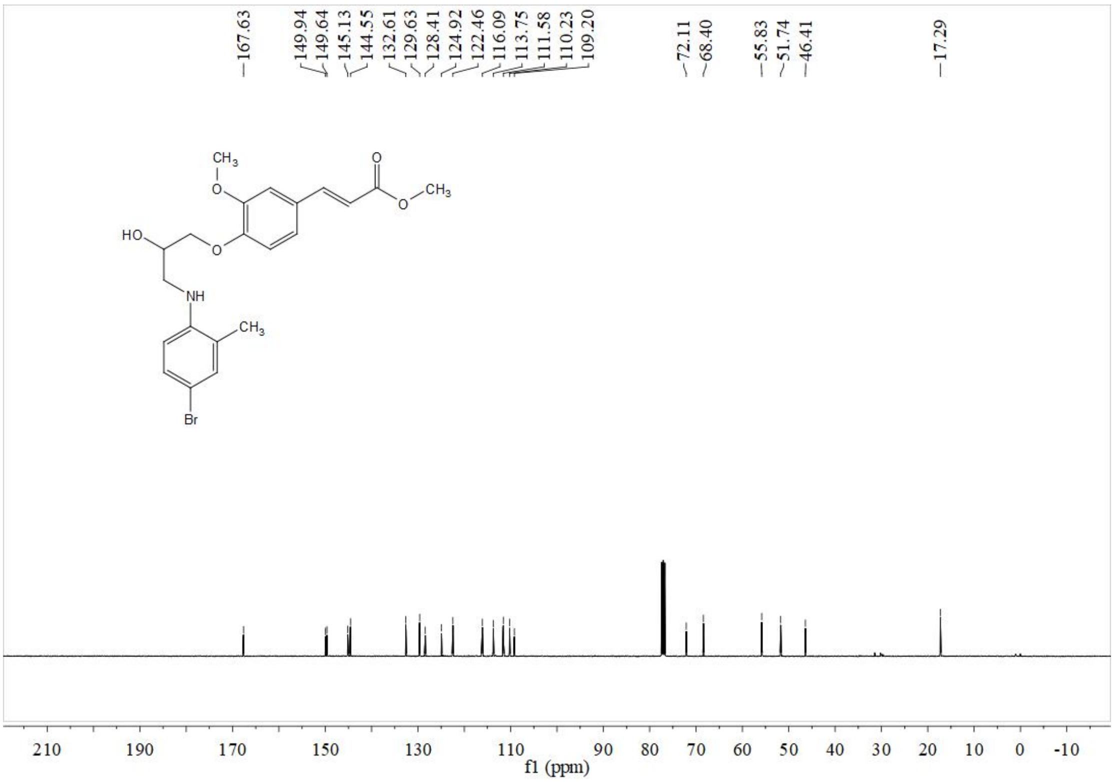

Figure S49 ^{19}F NMR Spectrum of **D18**

D19

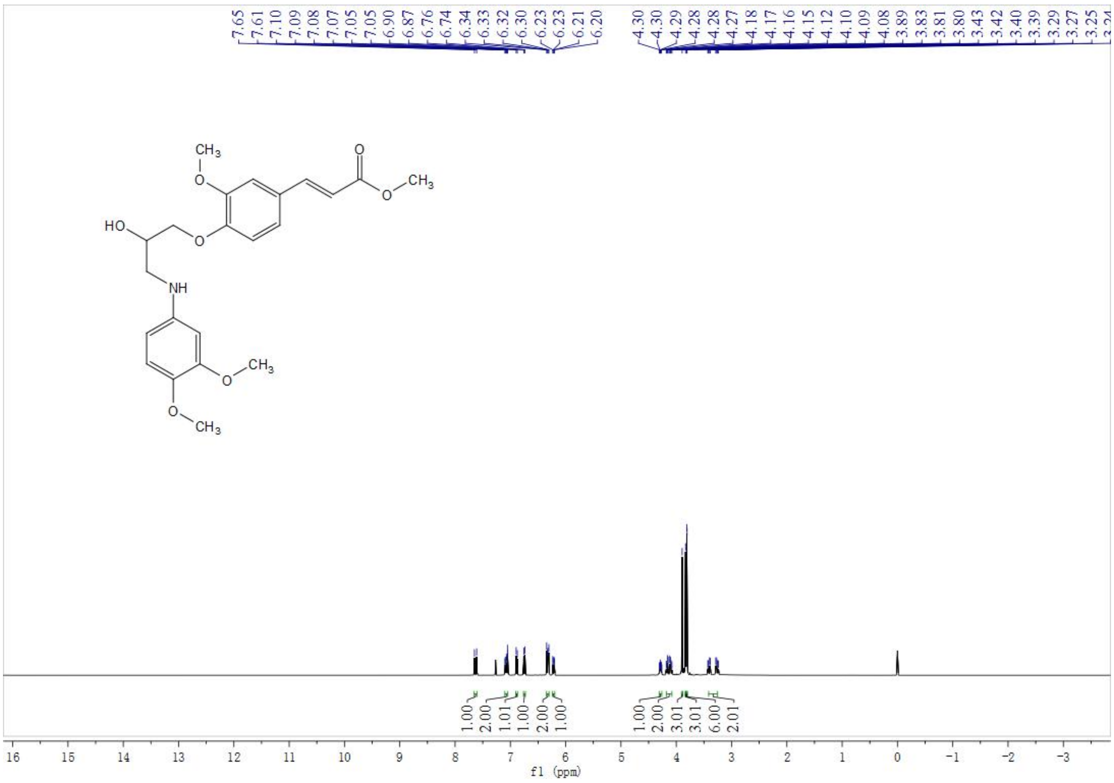

Figure S50 ^{1}H NMR Spectrum of **D19**

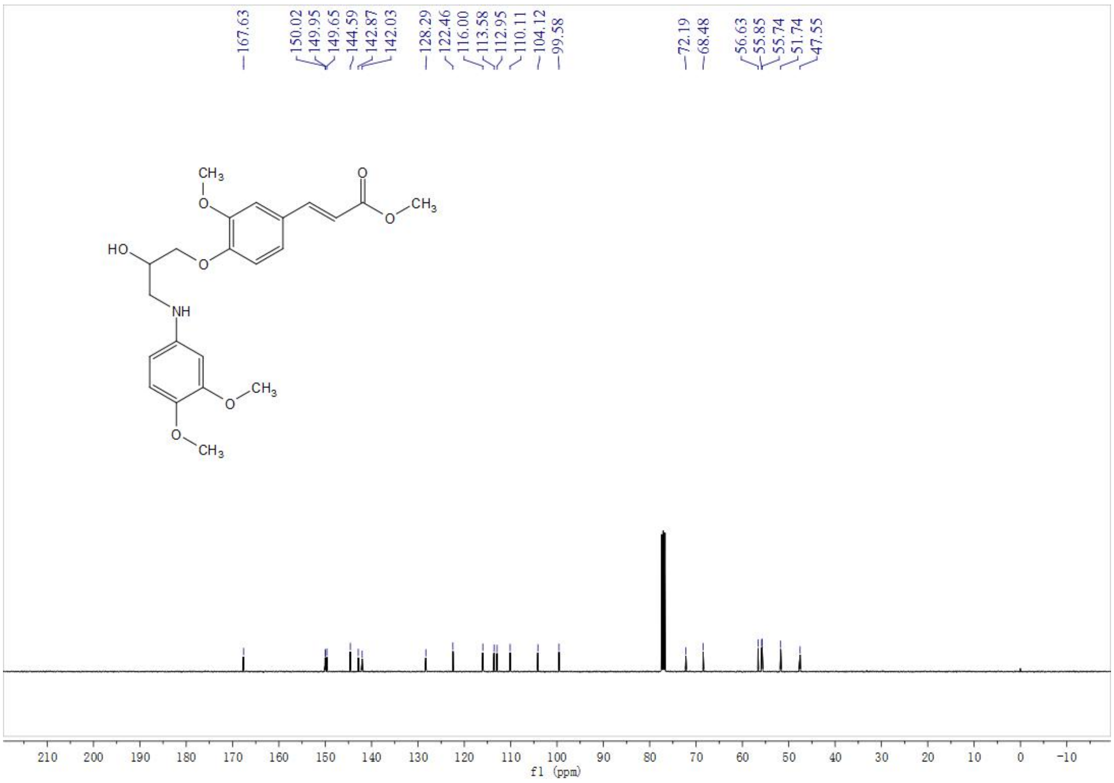

Figure S51 ^{13}C NMR Spectrum of **D19**

D20

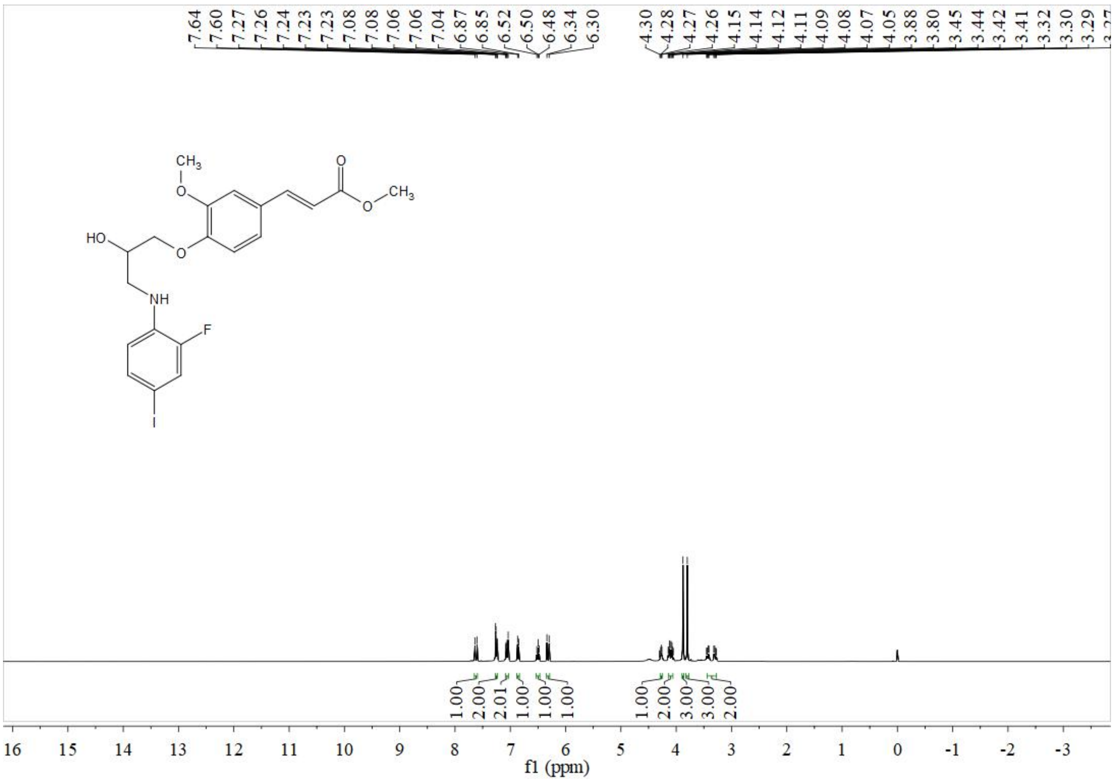

Figure S52 ^{1}H NMR Spectrum of **D20**

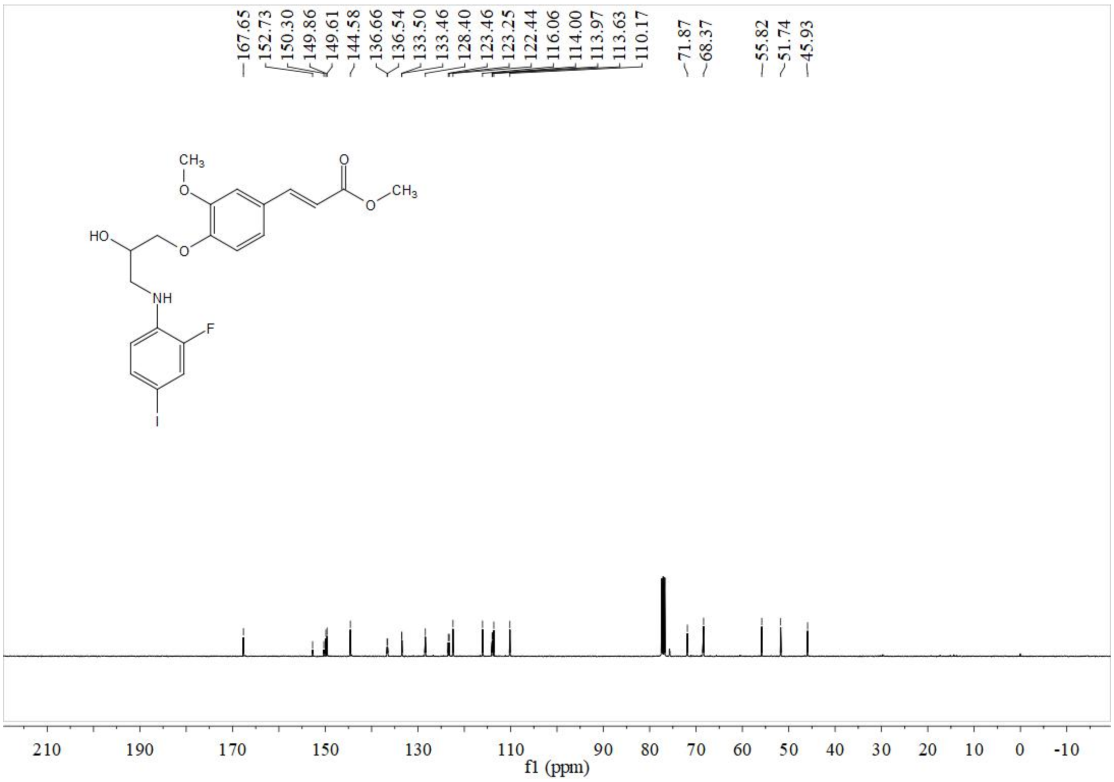

Figure S53 ^{13}C NMR Spectrum of **D20**

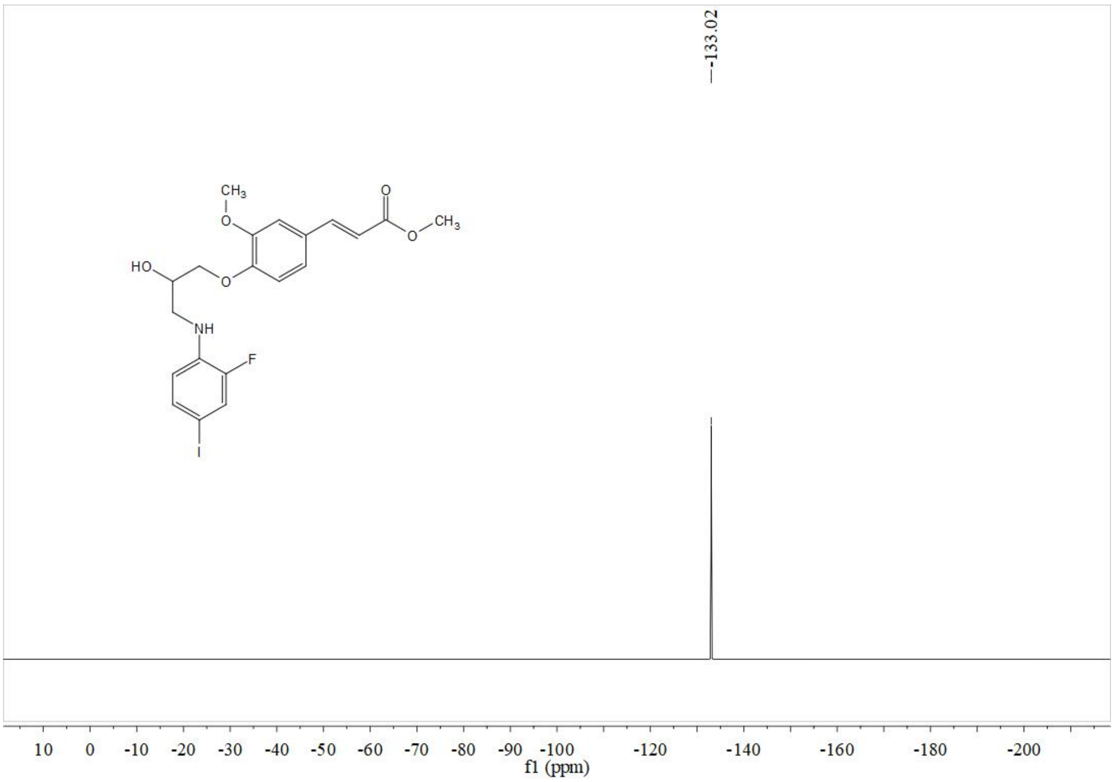

Figure S54 ^{19}F NMR Spectrum of **D20**

D21

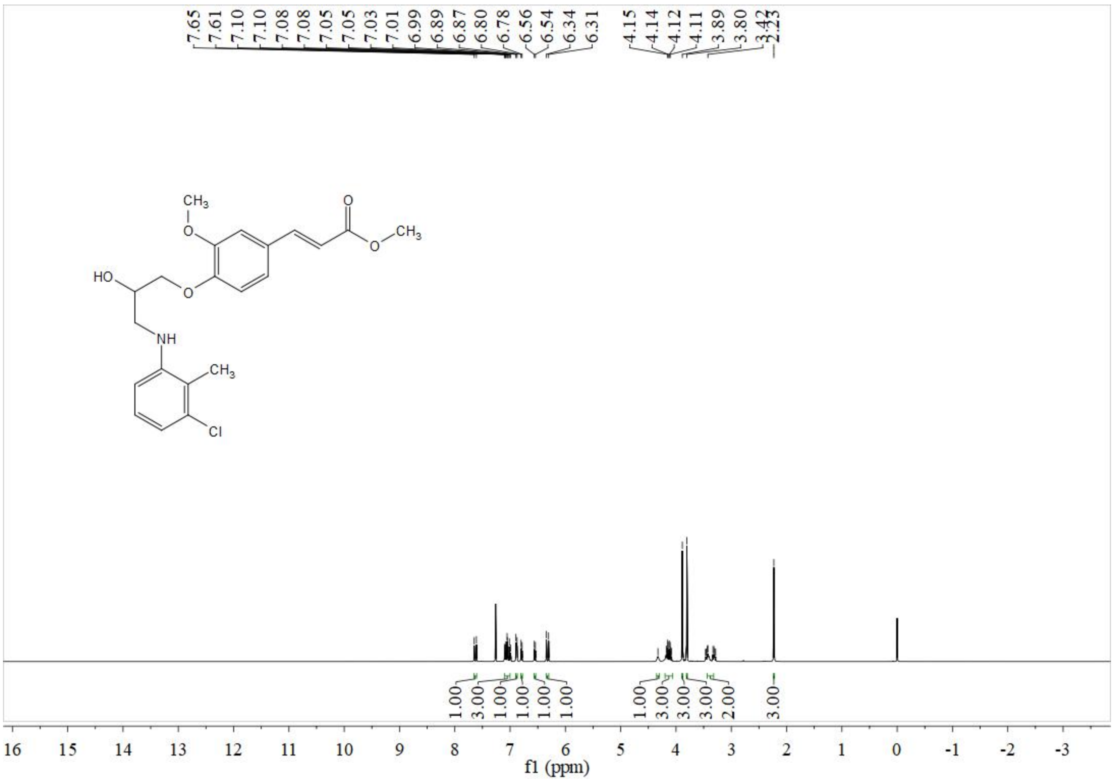

Figure S55 ^{1}H NMR Spectrum of **D21**

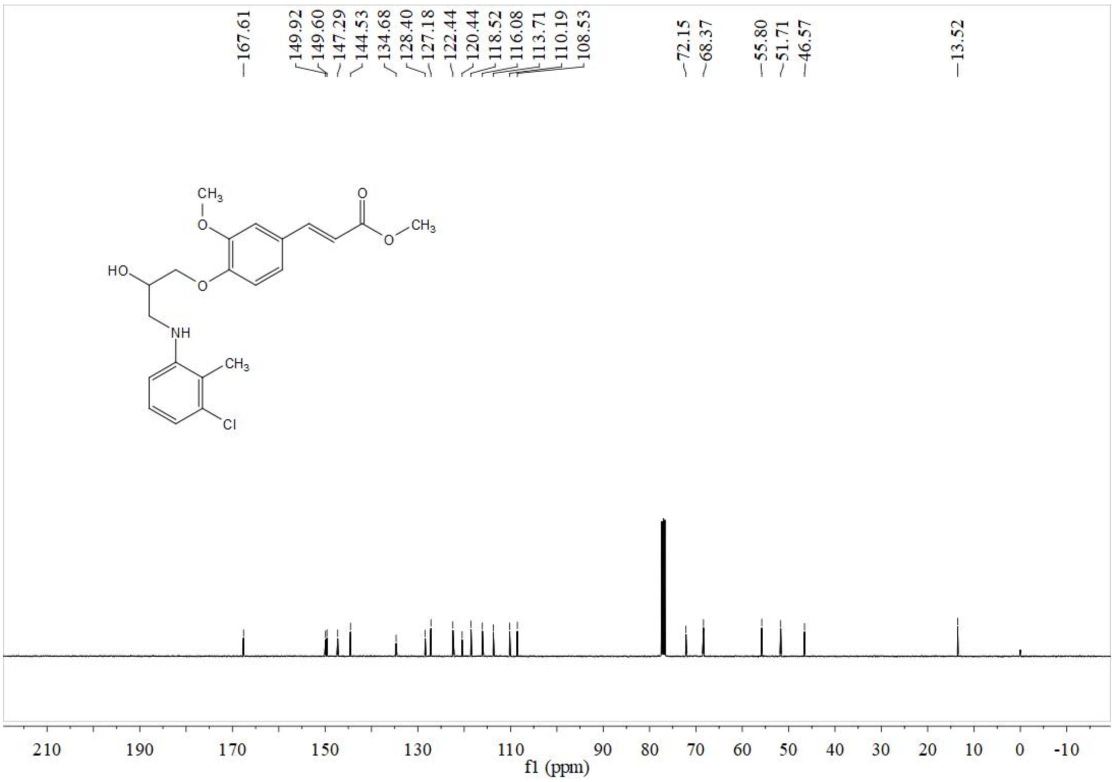

Figure S56 ^{13}C NMR Spectrum of **D21**

D22

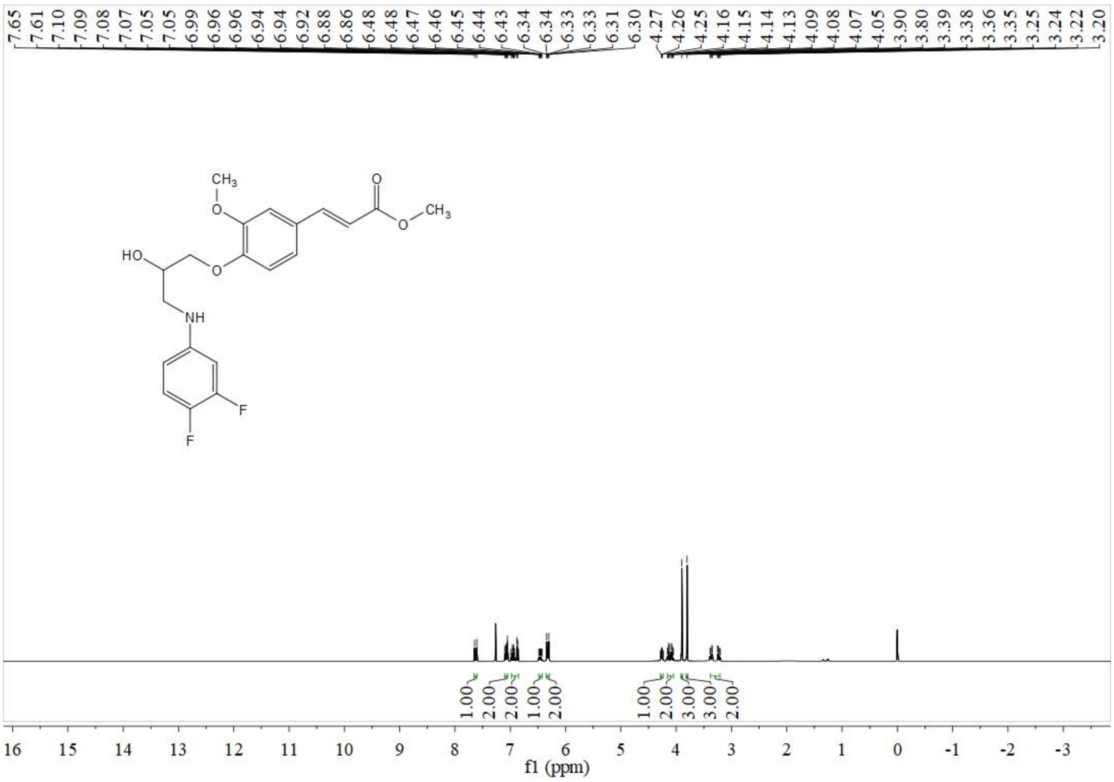

Figure S57 ^{1}H NMR Spectrum of **D22**

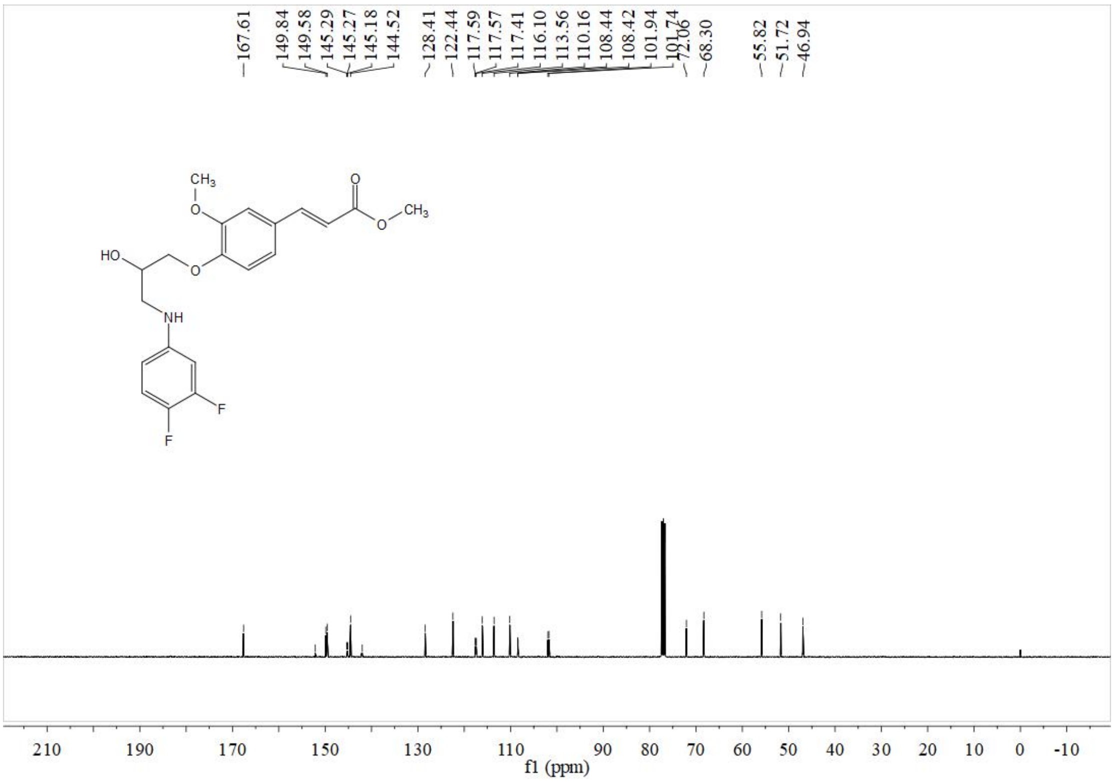

Figure S58 ^{13}C NMR Spectrum of **D22**

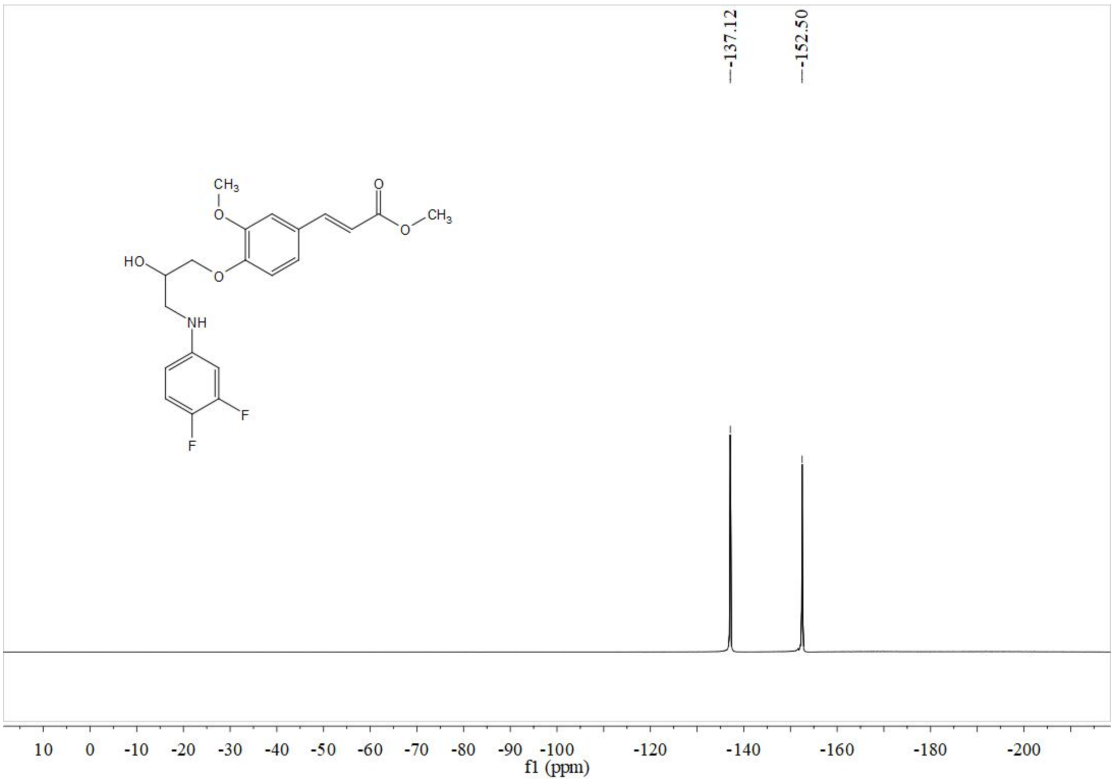

Figure S59 ^{19}F NMR Spectrum of **D22**

D23

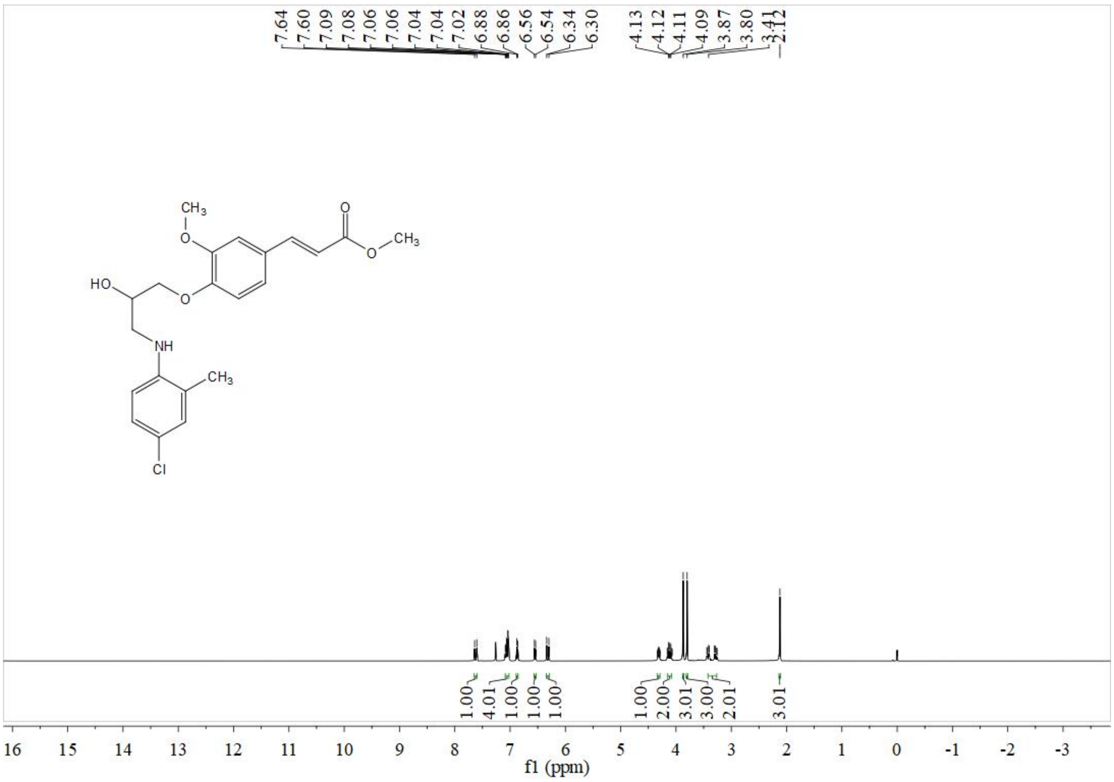

Figure S60 ^{1}H NMR Spectrum of **D23**

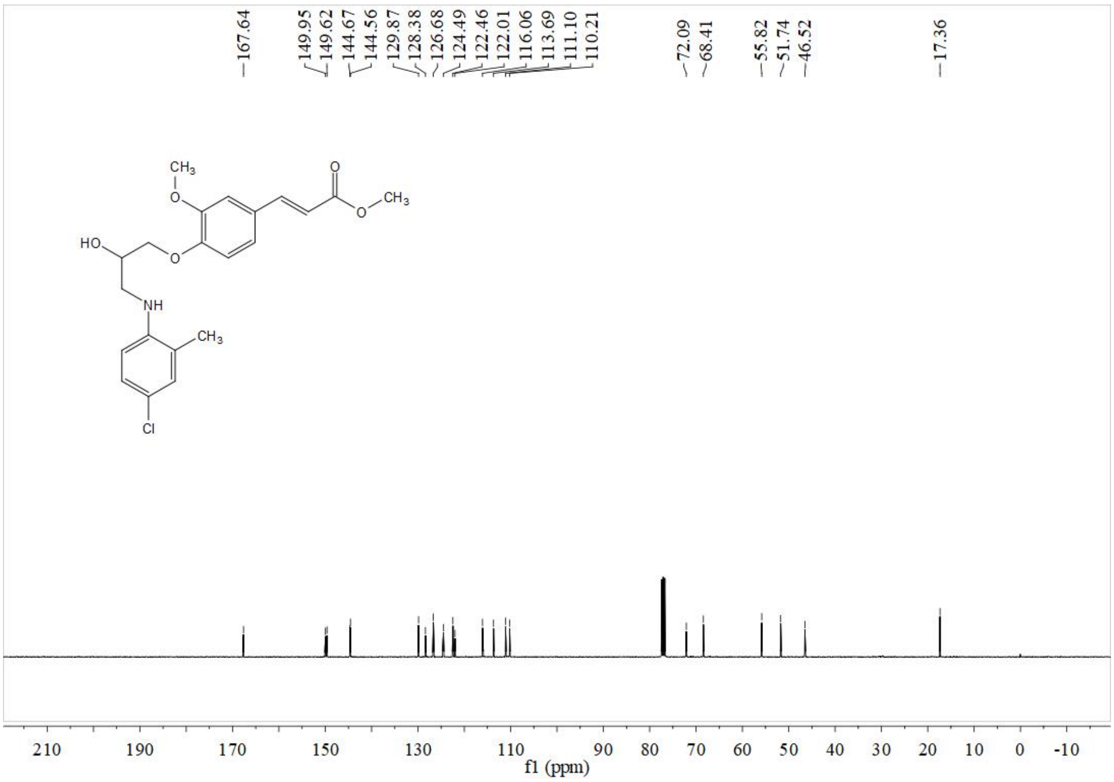

Figure S61 ^{13}C NMR Spectrum of **D23**

D24

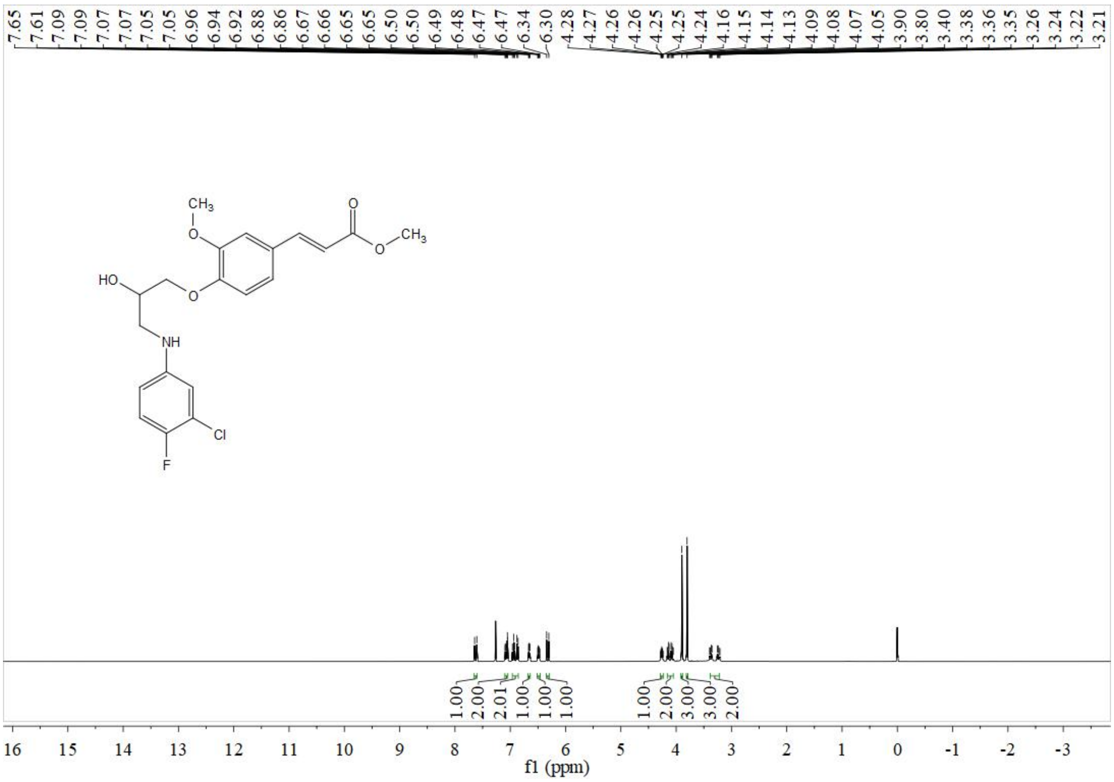

Figure S62 ^{1}H NMR Spectrum of **D24**

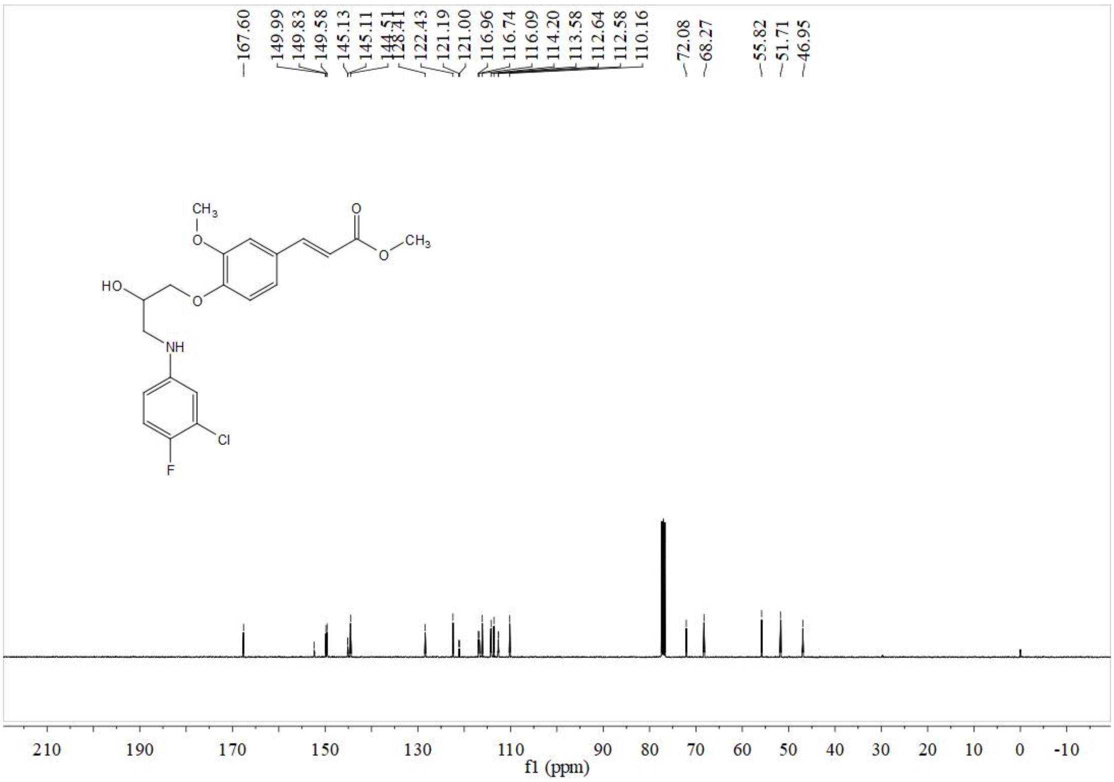

Figure S63 ^{13}C NMR Spectrum of **D24**

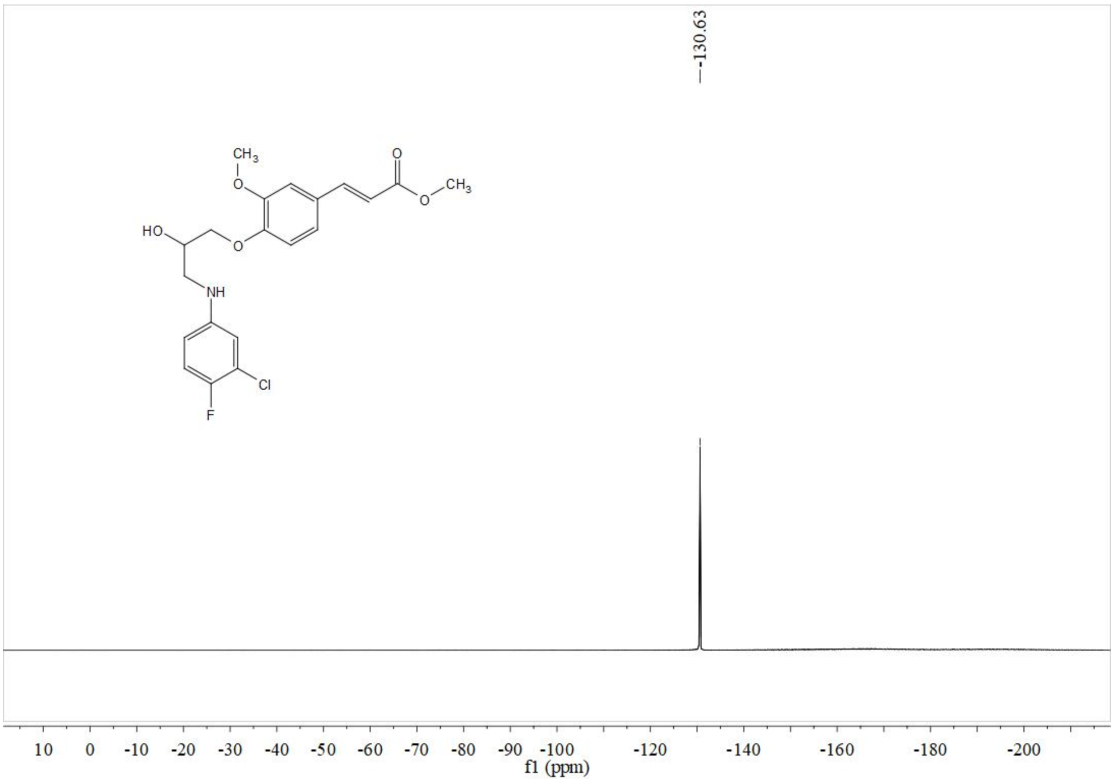

Figure S64 ^{19}F NMR Spectrum of **D24**

2、HRMS spectra of the title compounds D1 - D24

D1

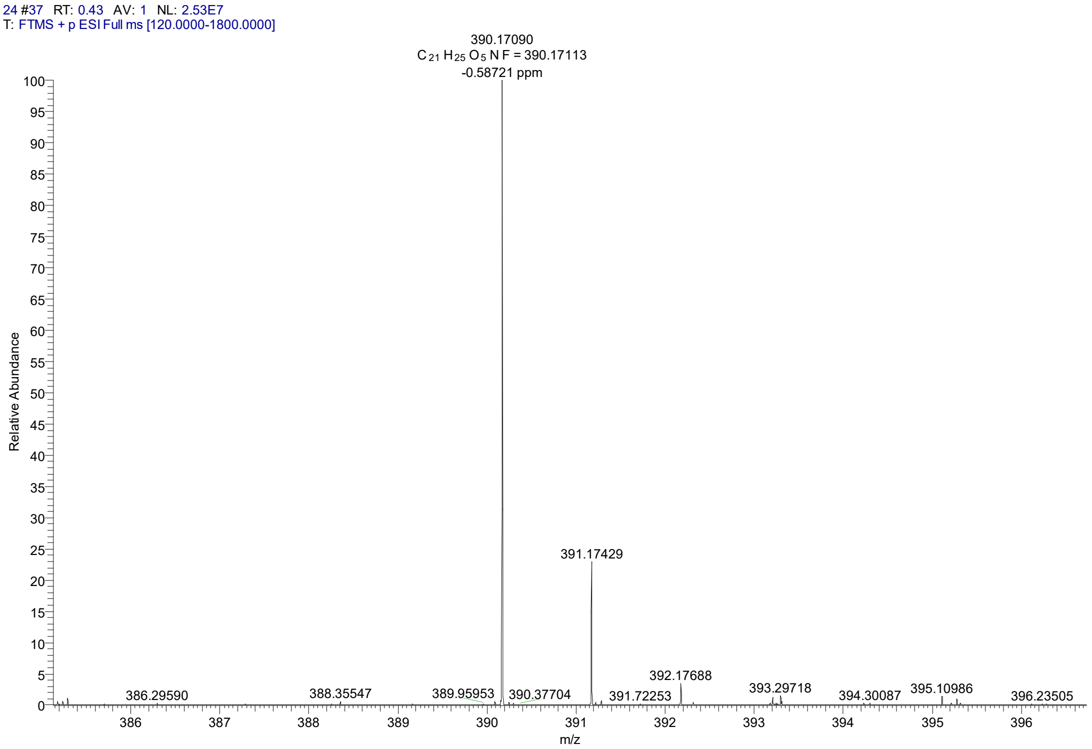

Figure S65 HRMS Spectrum of **D1**

D2

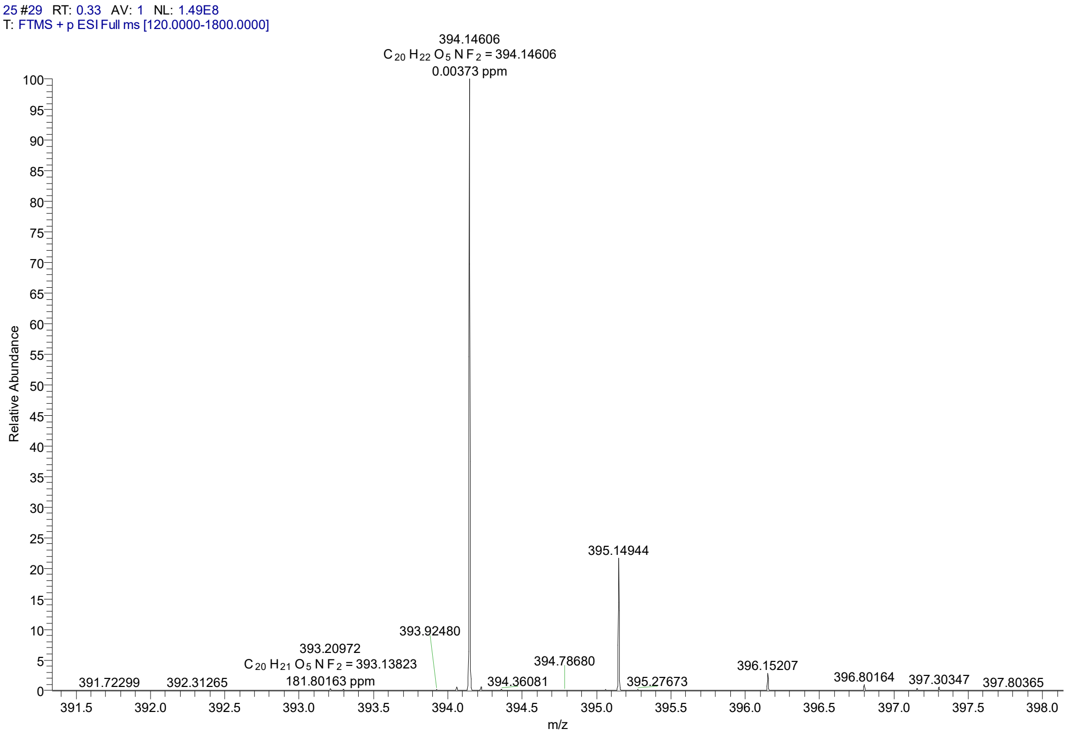

Figure S66 HRMS Spectrum of **D2**

D3

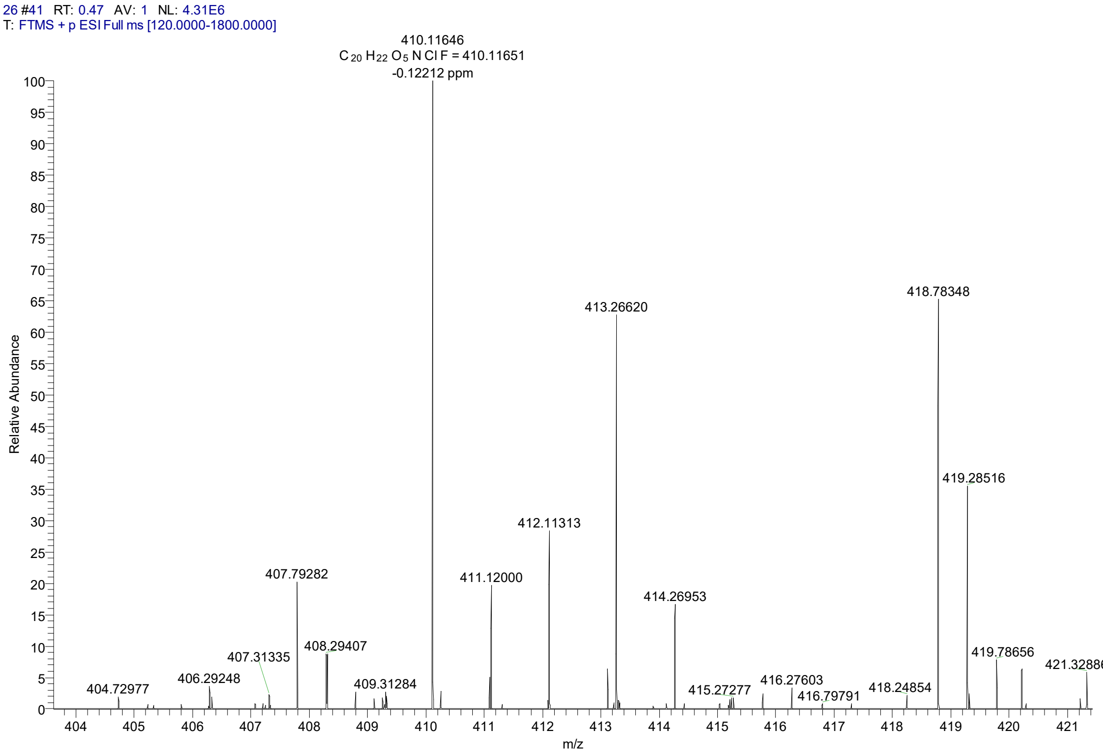

Figure S67 HRMS Spectrum of **D3**

D4

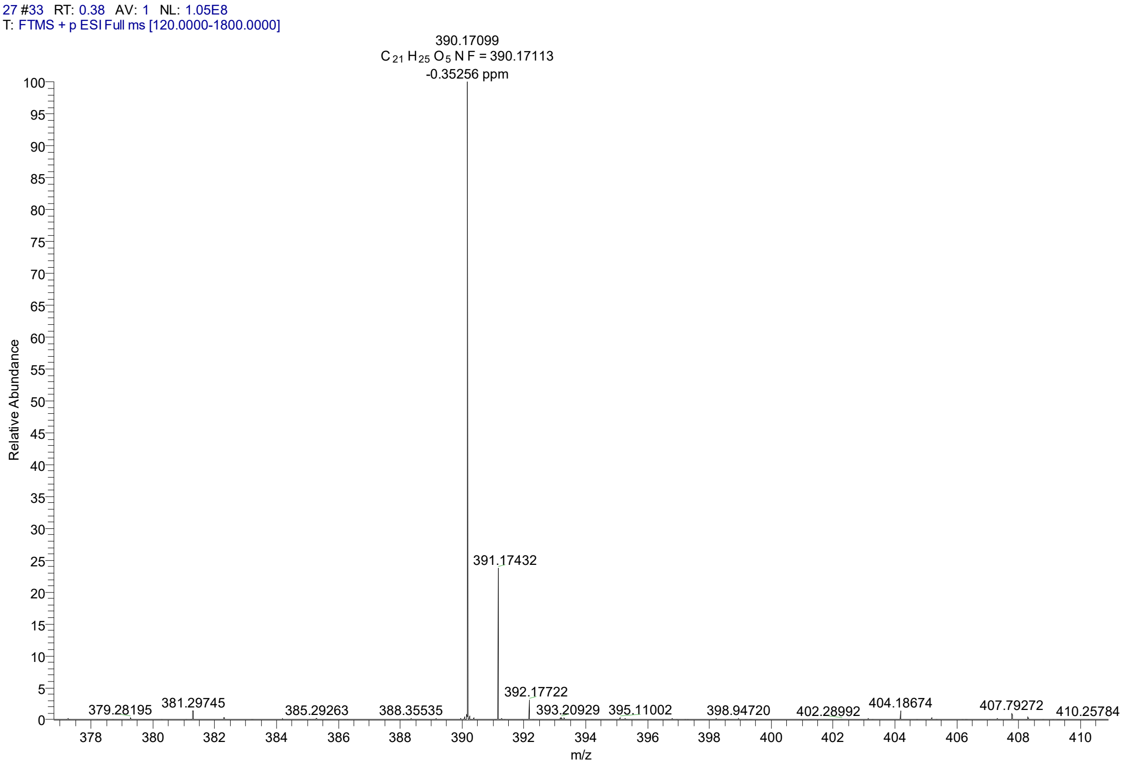

Figure S68 HRMS Spectrum of **D4**

D5

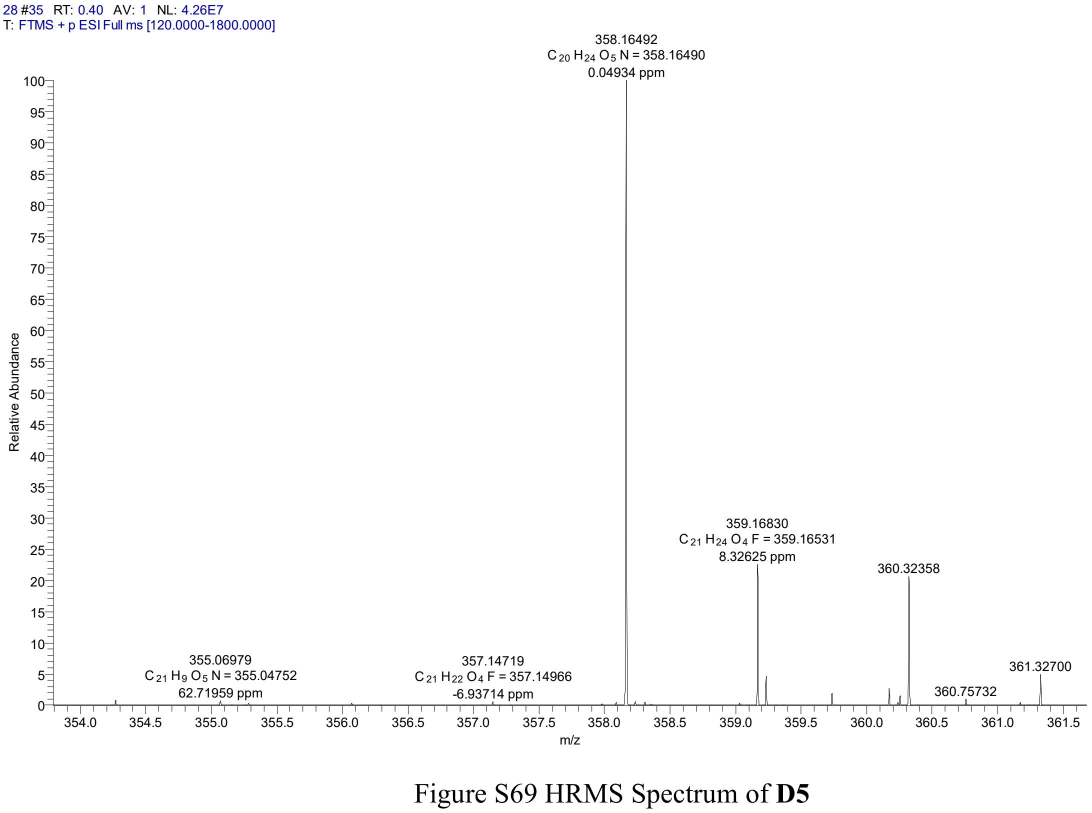

Figure S69 HRMS Spectrum of **D5**

D6

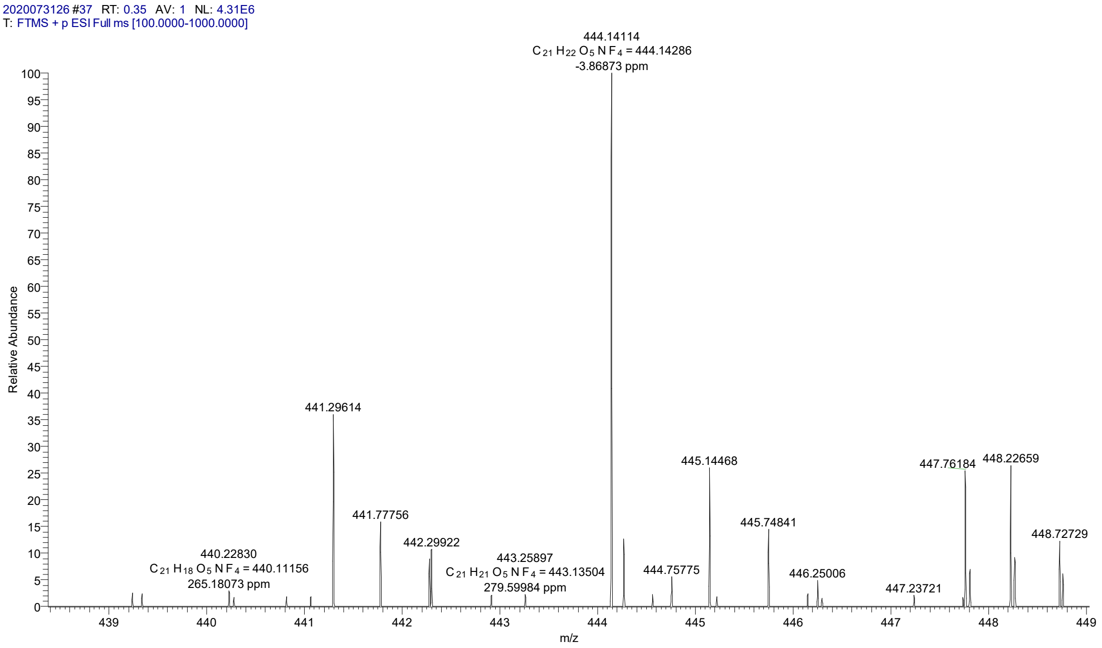

Figure S70 HRMS Spectrum of **D6**

D7

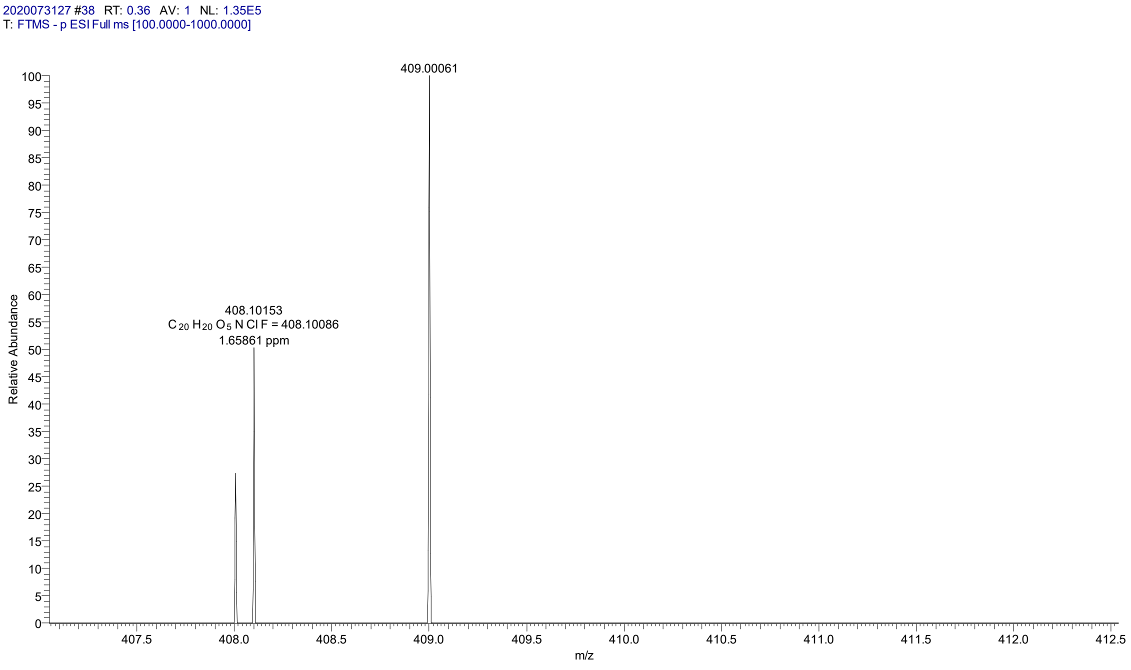

Figure S71 HRMS Spectrum of **D7**

D8

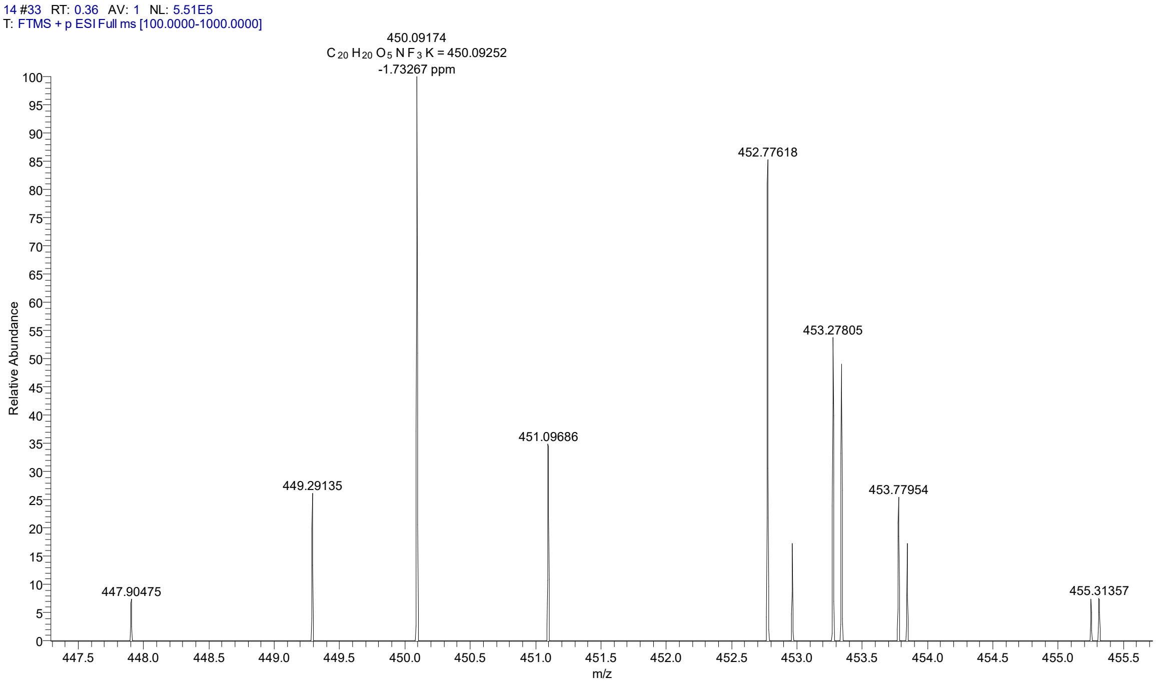

Figure S72 HRMS Spectrum of **D8**

D9

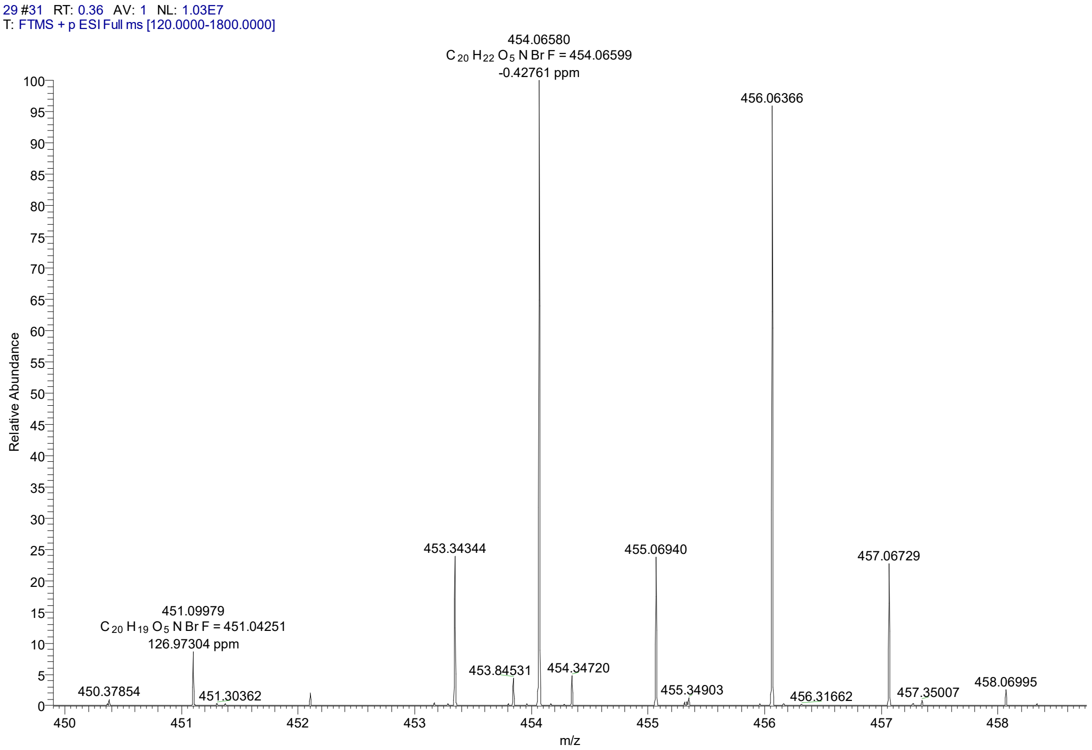

Figure S73 HRMS Spectrum of **D9**

D10

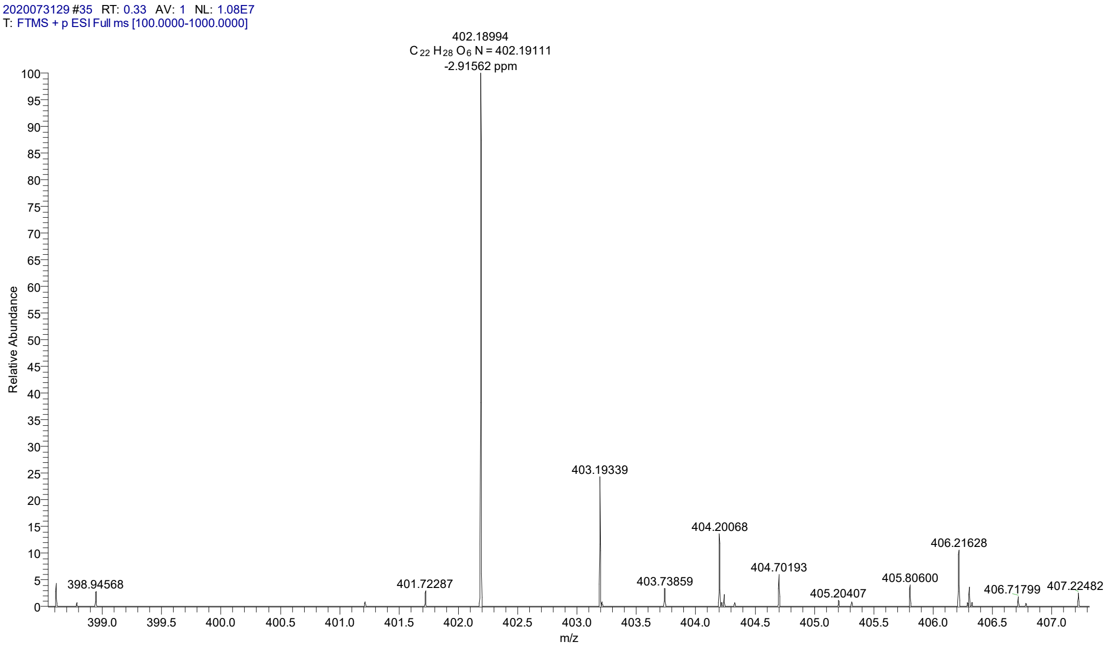

Figure S74 HRMS Spectrum of **D10**

D11

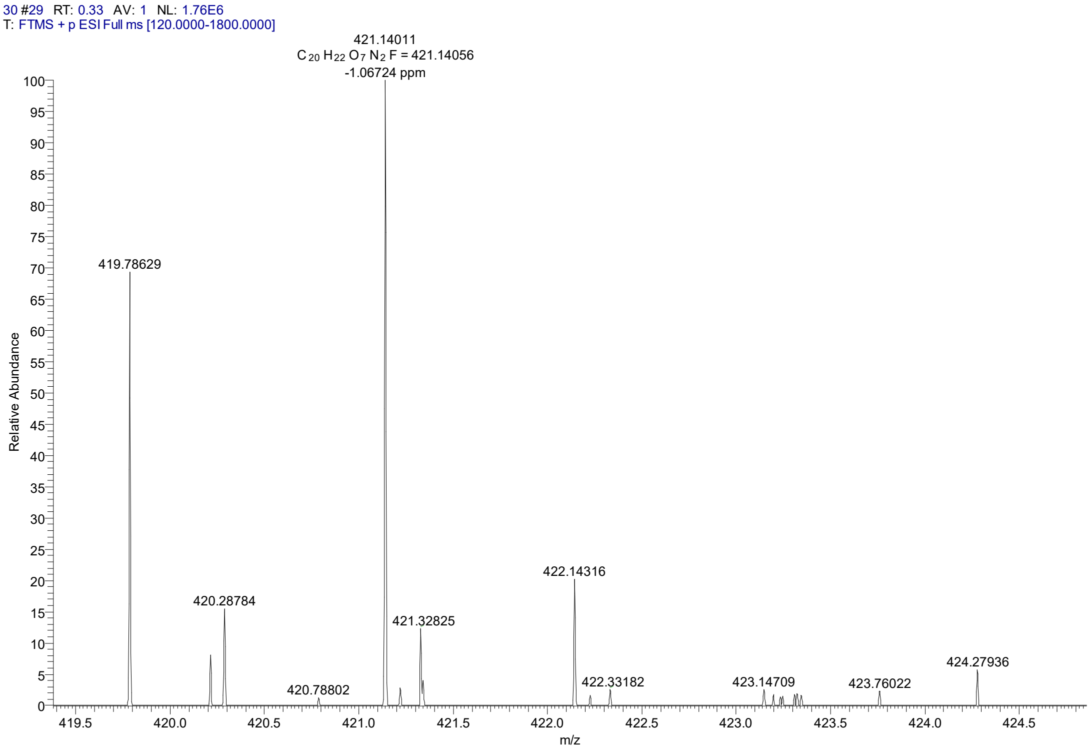

Figure S75 HRMS Spectrum of **D11**

D12

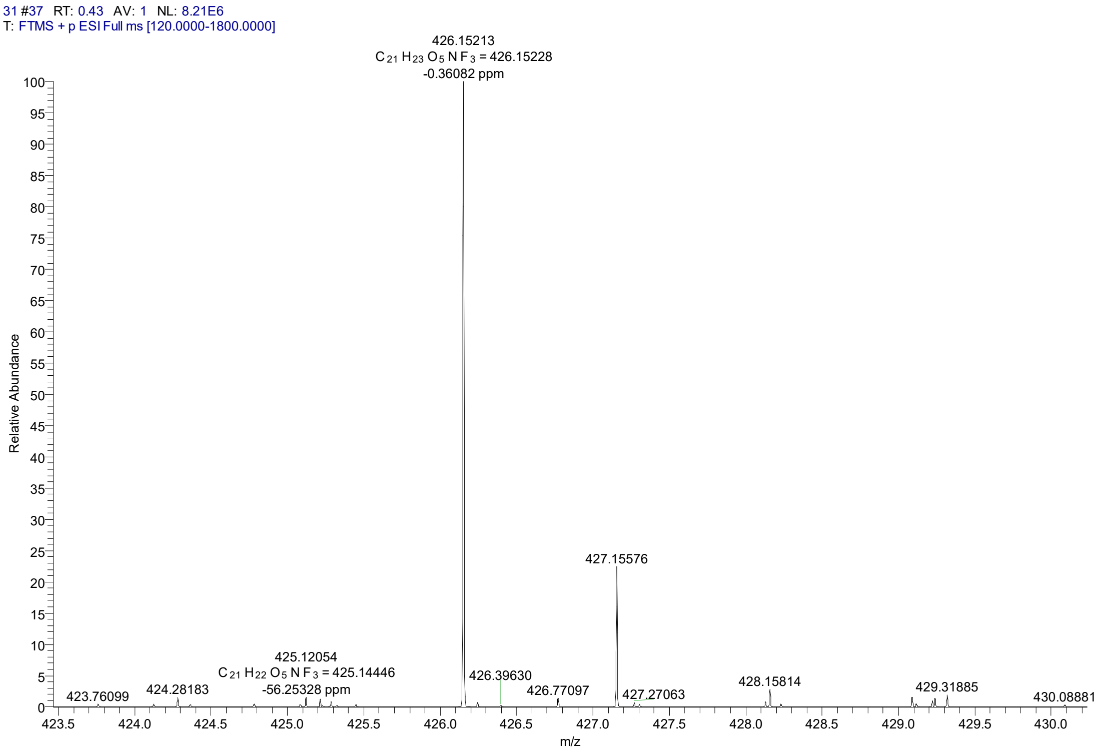

Figure S76 HRMS Spectrum of **D12**

D13

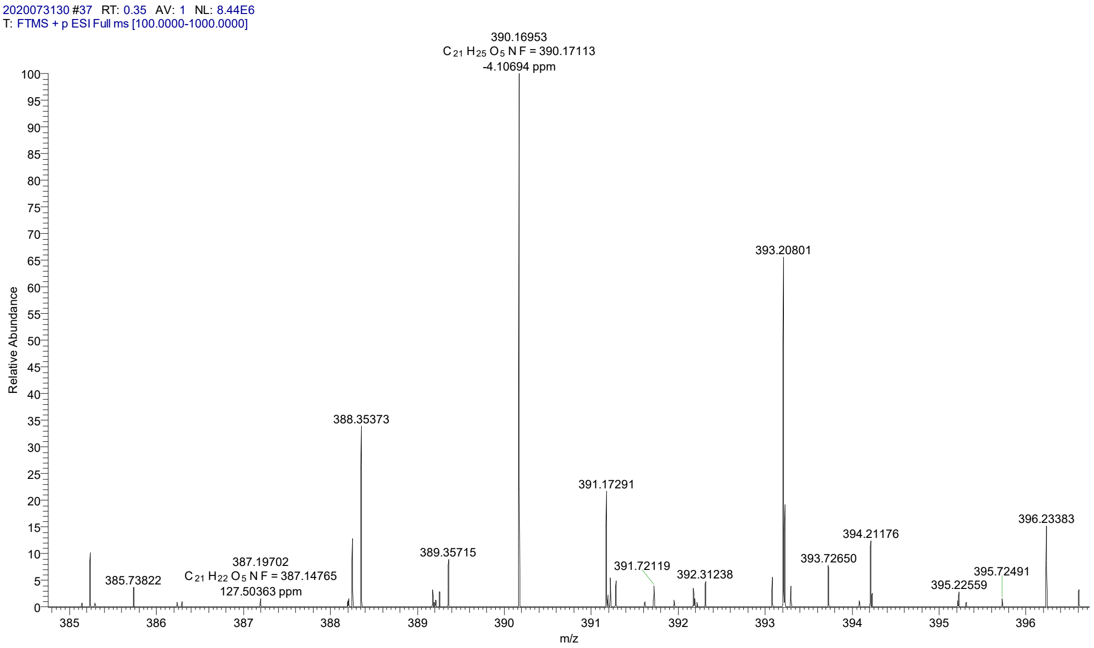

Figure S77 HRMS Spectrum of **D13**

D14

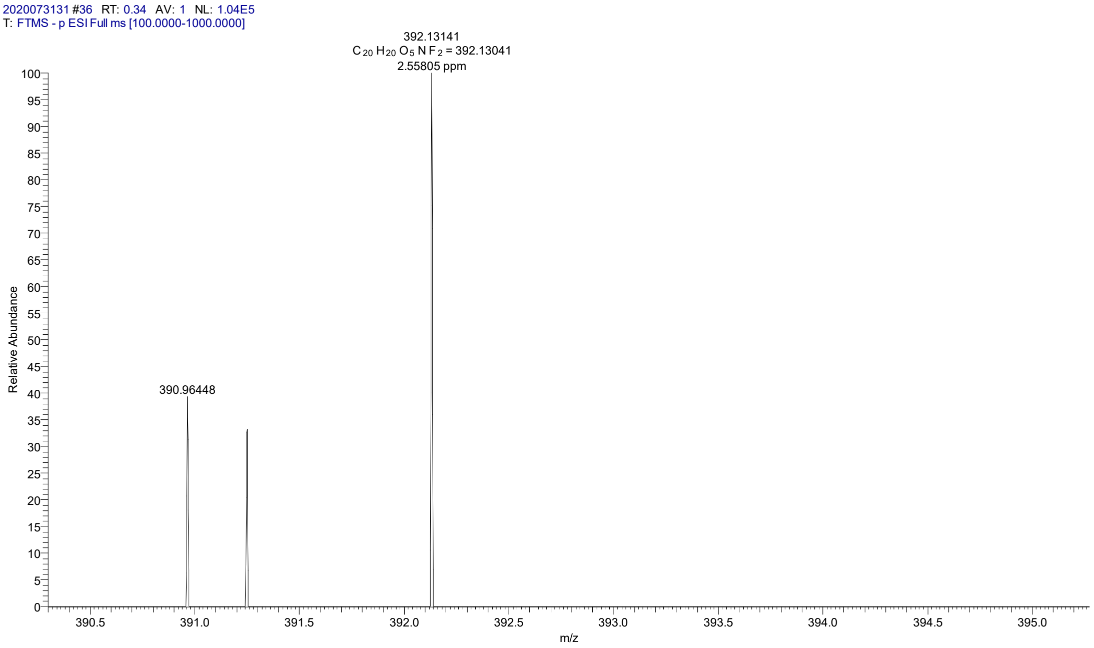

Figure S78 HRMS Spectrum of **D14**

D15

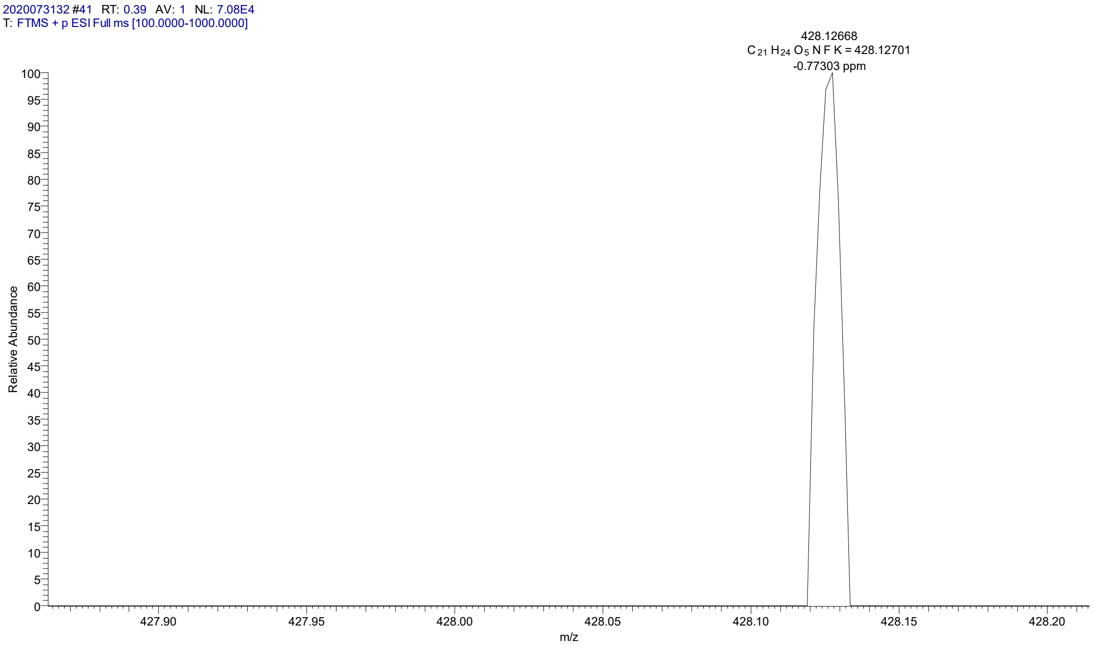

Figure S79 HRMS Spectrum of **D15**

D16

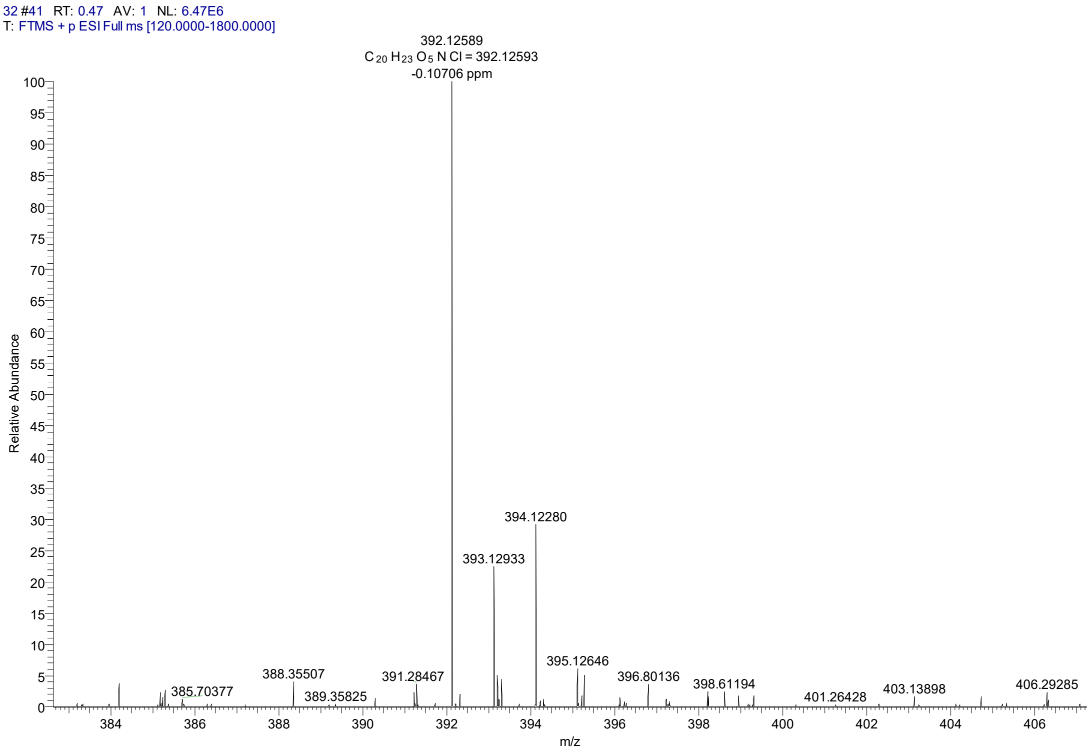

Figure S80 HRMS Spectrum of **D16**

D17

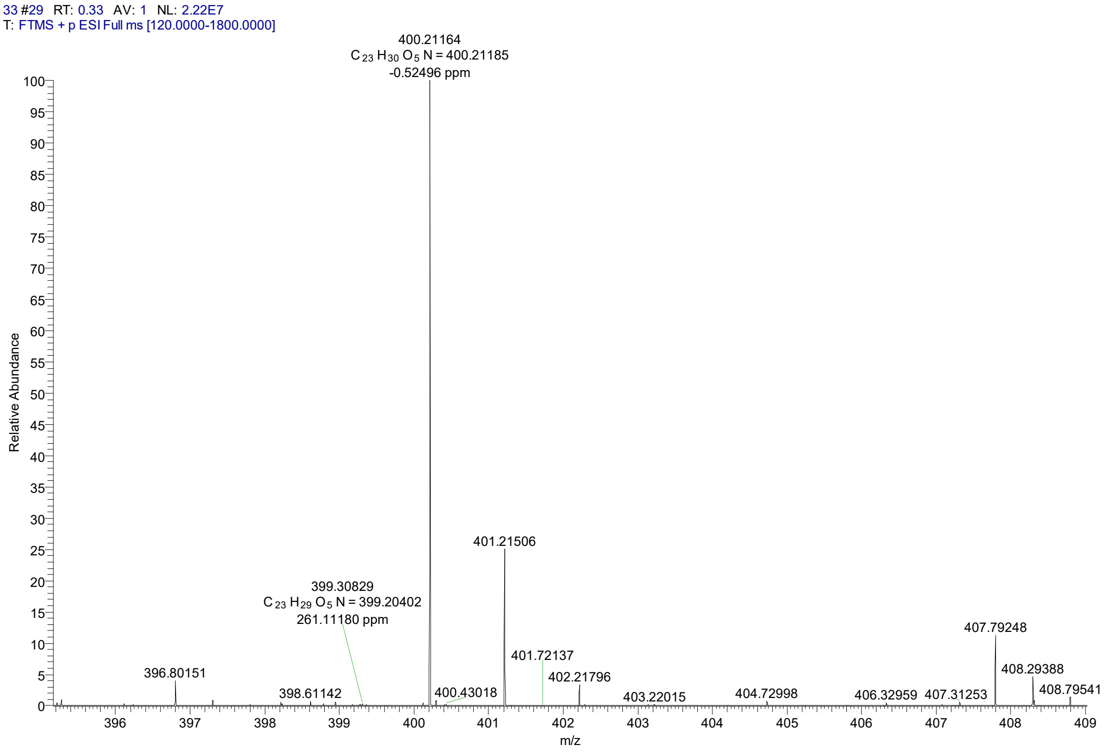

Figure S81 HRMS Spectrum of **D17**

D18

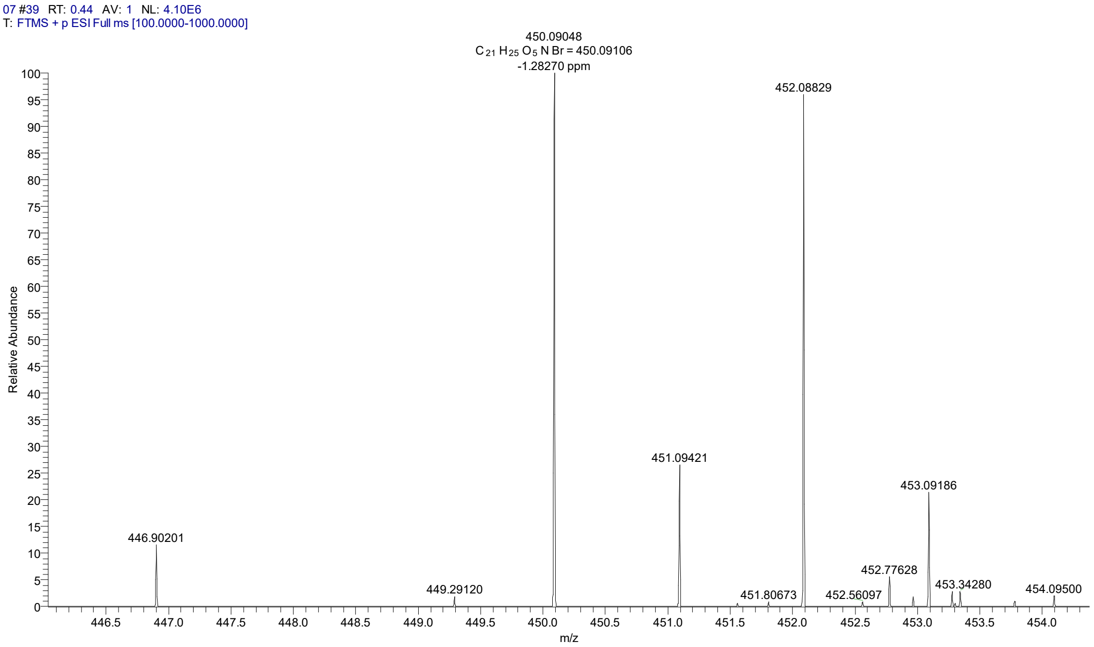

Figure S82 HRMS Spectrum of **D18**

D19

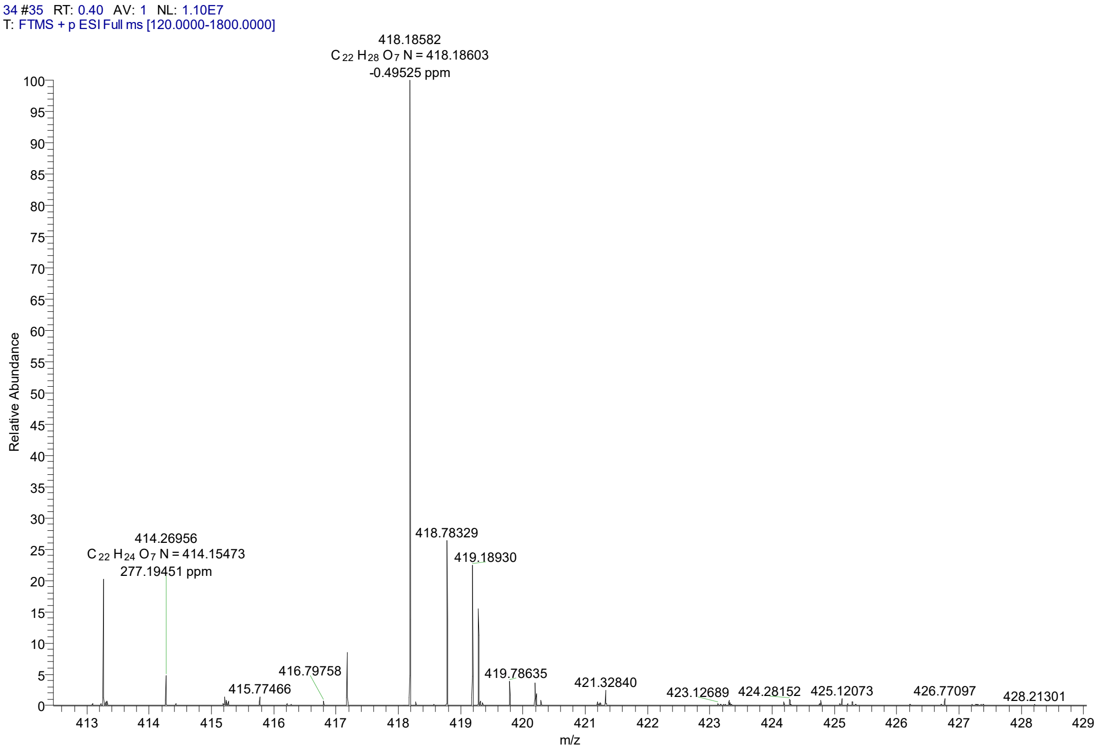

Figure S83 HRMS Spectrum of **D19**

D20

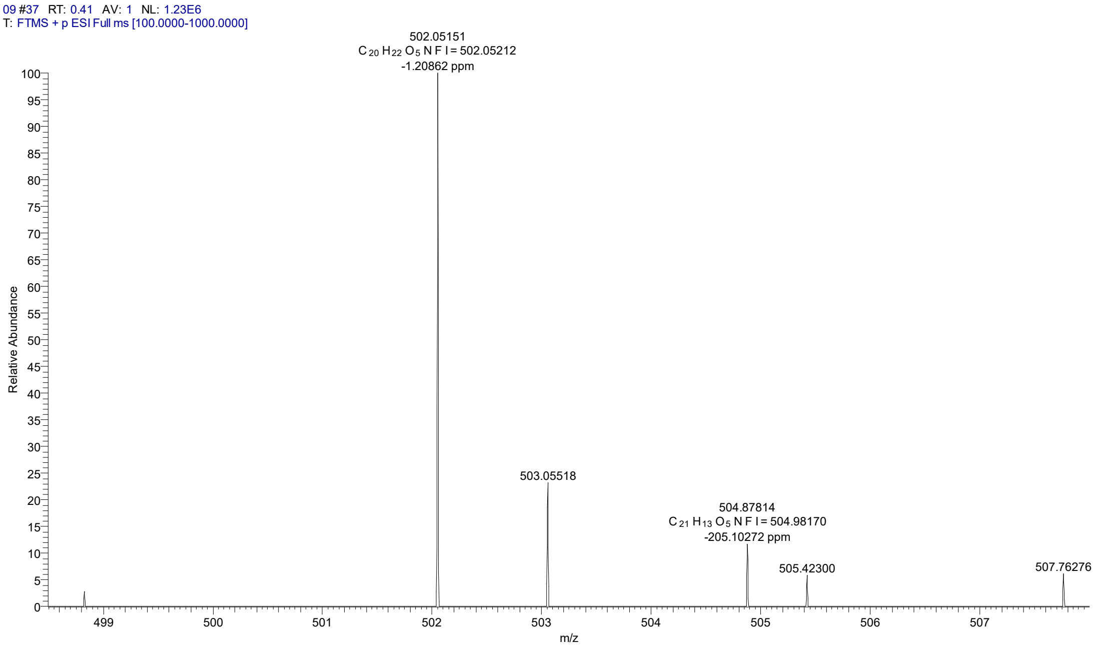

Figure S84 HRMS Spectrum of **D20**

D21

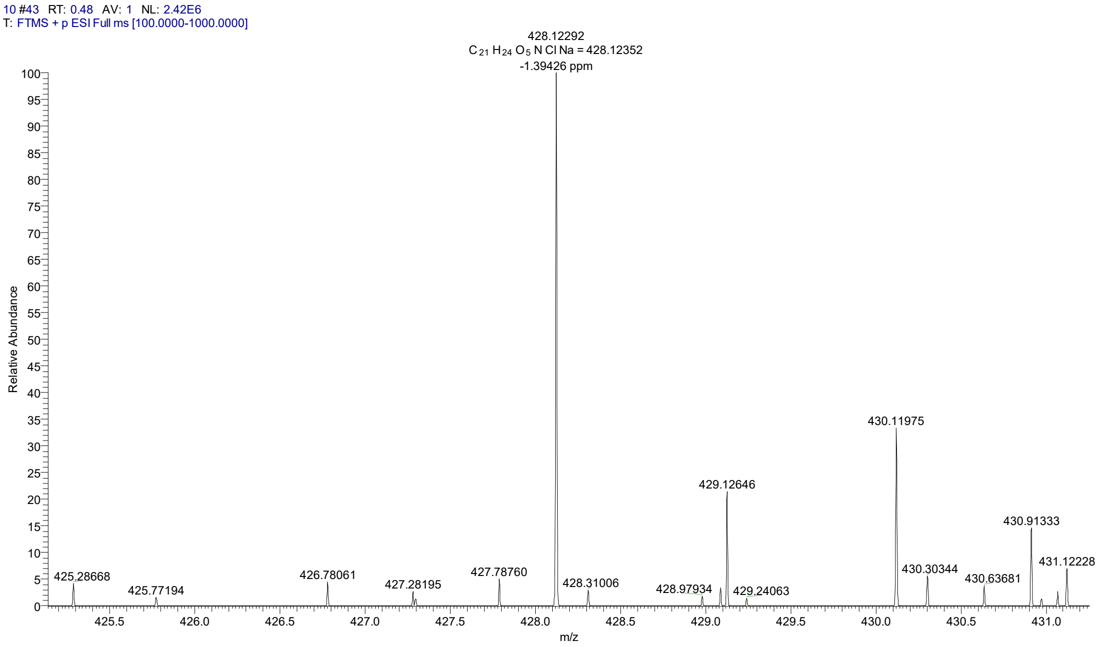

Figure S85 HRMS Spectrum of **D21**

D22

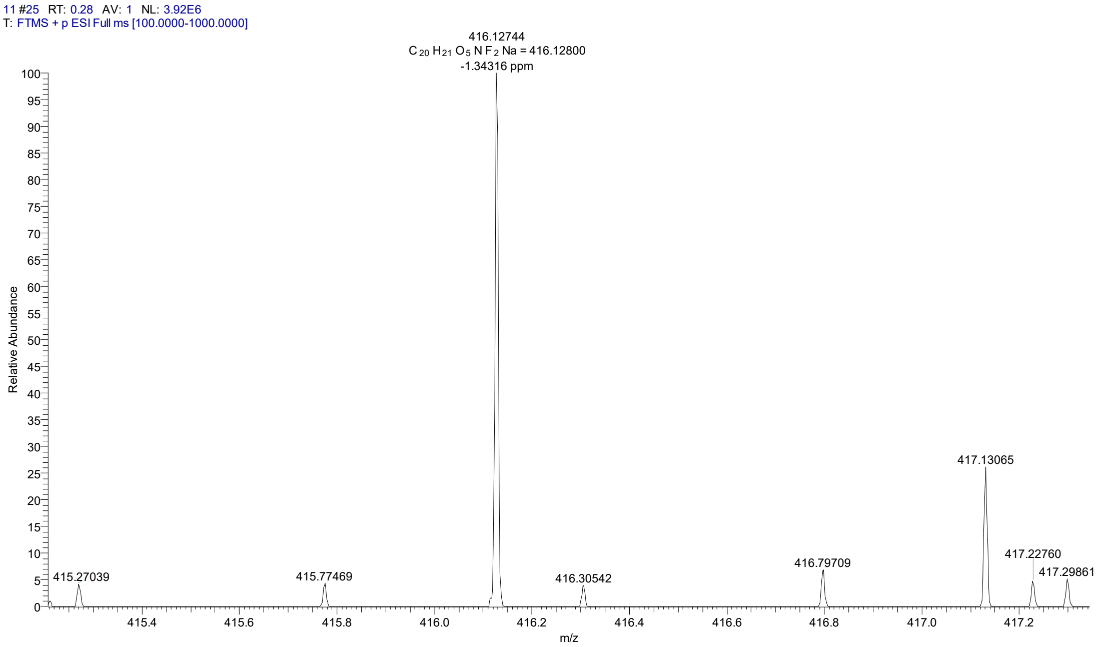

Figure S86 HRMS Spectrum of **D22**

D23

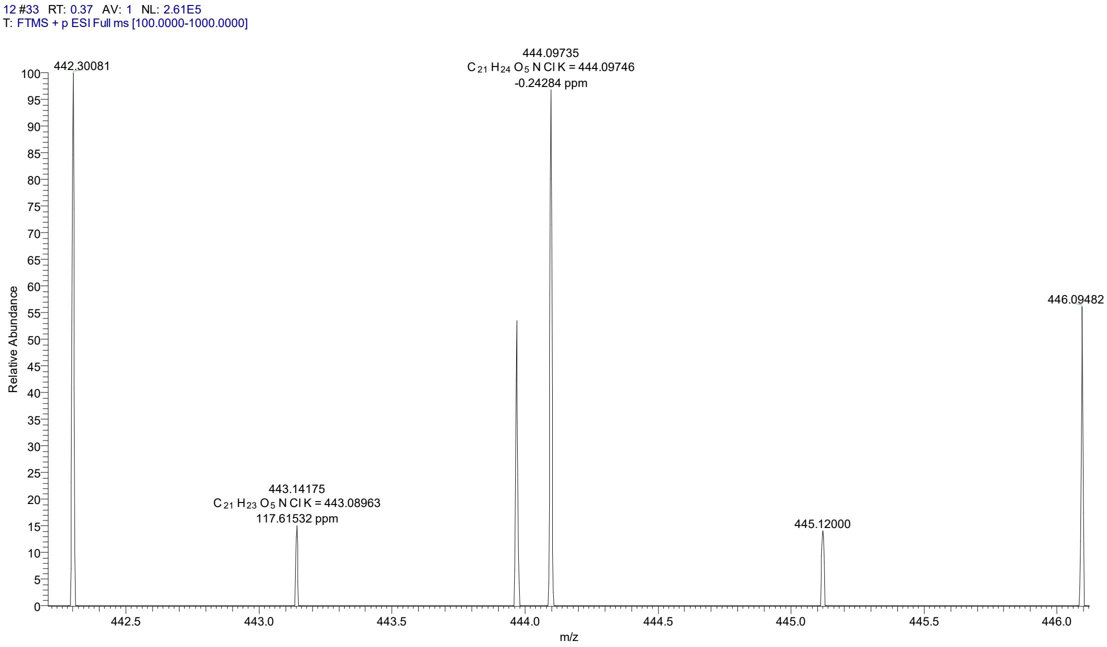

Figure S87 HRMS Spectrum of **D23**

D24

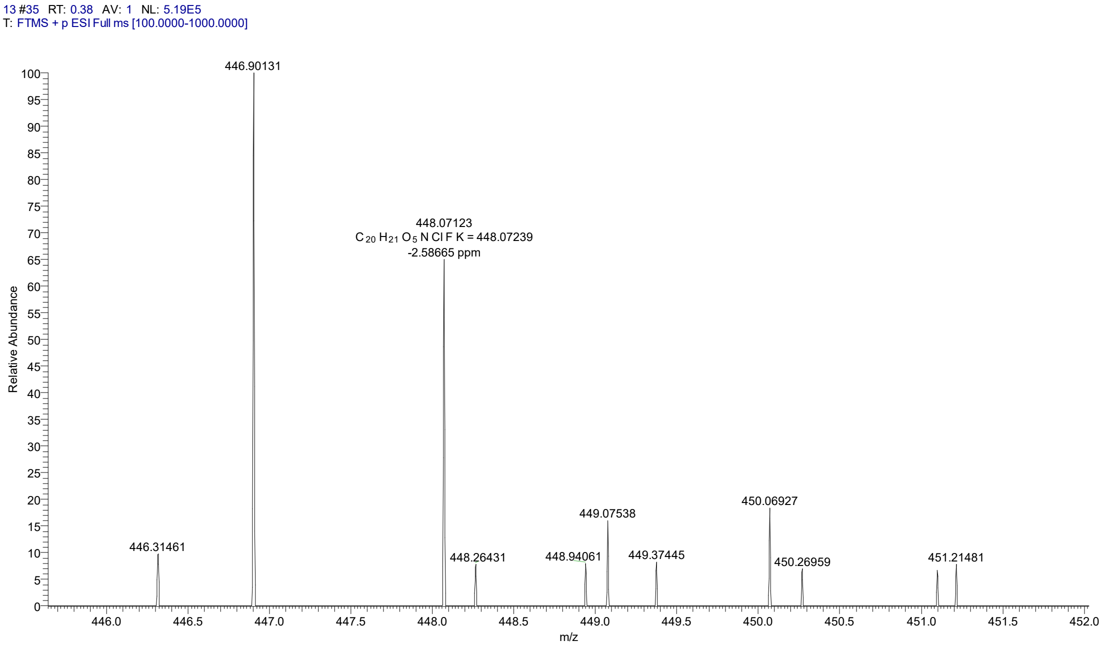

Figure S88 HRMS Spectrum of **D24**
